# Deep Learning Model Coupling Wearable Bioelectric and Mechanical Sensors for Refined Muscle Strength Assessment

**DOI:** 10.34133/research.0366

**Published:** 2024-05-23

**Authors:** Chengyu Li, Tingyu Wang, Siyu Zhou, Yanshuo Sun, Zijie Xu, Shuxing Xu, Sheng Shu, Yi Zhao, Bing Jiang, Shiwang Xie, Zhuoran Sun, Xiaowei Xu, Weishi Li, Baodong Chen, Wei Tang

**Affiliations:** ^1^ Beijing Institute of Nanoenergy and Nanosystems, Chinese Academy of Sciences, Beijing 101400, China.; ^2^ School of Nanoscience and Technology, University of Chinese Academy of Sciences, Beijing 100049, China.; ^3^ Department of Orthopaedics, Peking University Third Hospital, Beijing 100191, China.; ^4^ Engineering Research Center of Bone and Joint Precision Medicine, Ministry of Education, Beijing, China.; ^5^ Beijing Key Laboratory of Spinal Disease Research, Beijing, China.; ^6^ Center on Nanoenergy Research, School of Physical Science and Technology, Guangxi University, Nanning 530004, China.; ^7^ Guangdong Provincial People’s Hospital, Guangdong Academy of Medical Sciences, Guangzhou, China.

## Abstract

Muscle strength (MS) is related to our neural and muscle systems, essential for clinical diagnosis and rehabilitation evaluation. Although emerging wearable technology seems promising for MS assessment, problems still exist, including inaccuracy, spatiotemporal differences, and analyzing methods. In this study, we propose a wearable device consisting of myoelectric and strain sensors, synchronously acquiring surface electromyography and mechanical signals at the same spot during muscle activities, and then employ a deep learning model based on temporal convolutional network (TCN) + Transformer (Tcnformer), achieving accurate grading and prediction of MS. Moreover, by combining with deep clustering, named Tcnformer deep cluster (TDC), we further obtain a 25-level classification for MS assessment, refining the conventional 5 levels. Quantification and validation showcase a patient’s postoperative recovery from level 3.2 to level 3.6 in the first few days after surgery. We anticipate that this system will importantly advance precise MS assessment, potentially improving relevant clinical diagnosis and rehabilitation outcomes.

## Introduction

Muscle strength (MS), a fundamental representation of human motor function, is closely bound up with our neural and muscular systems [1,2]. Accurate assessment and grading of MS are critical for clinical operations, e.g., offering essential information for preoperative evaluation, therapeutic interventions, and rehabilitation assistance [3–6]. Currently, the Muscle Research Council scale has been developed in clinical practice based on the manual muscle testing (MMT) for assessing MS [7]. Due to its convenience and effectiveness, this approach has been widely applied in clinical examinations and diagnoses of neuromuscular, musculoskeletal, metabolic, and joint diseases, among others [8,9]. Efforts are also underway to explore the utilization of digital muscle testing devices [10–13], such as dynamometers or other electrical force-based devices. These innovative devices have greatly facilitated the digitalization and convenience of MS assessment. Nevertheless, the force-based device still presents several challenges: It is not universally applicable, as it faces limitations in assessing patients with weaker muscle function, e.g., level 1 or 2 [14,15]. Furthermore, performing strength measure by dynamometers, patients are required to do continuous force resistance during test [16,17], which are intolerable for most patients. Hence, considering the need for continuous, accessible assessments that do not exacerbate patient discomfort, there is an urgent demand for a novel approach to MS evaluation.

Recently, the emergence of wearable sensors has yielded a promising approach [18–22]. For example, surface electromyography (sEMG) devices or strain sensors are investigated and applied in hand/limb motion sense [23–25], human–machine interaction [26,27], prosthesis control [28,29], and MS assessment [30,31]. The sEMG captures bioelectrical activity within the muscle. However, it is a composite signal, involving not only the main muscle neuroelectric signals but also the peripheral ones [32]. It might be influenced by the spatiotemporal differences due to myoelectric triggers and mechanical responses as the myofiber shortening [33–35]. As for the physical deformations of muscle, it is reported that there is a strong correlation between muscle deformation and skin strain signals, encompassing amplitude, velocity, and pause time, allowing for a comprehensive representation of the overall mechanical activity of the muscle [31,36,37]. Thus, strain sensors are utilized for the assessment of muscle function and strength [10,31]. However, the strain signal is susceptible to motion artifacts, environmental and material factors that can affect accuracy [31,38]. Thus, some researchers tried to combine sEMG with strain sensors and applied it in gesture recognition [39]. However, the homogeneous sensing unit might induce cross-interference, lowering the signal-to-noise ratio (SNR). More importantly, the captured neural and strain signals, possessing distinct features, are still analyzed independently, lacking effective and comprehensive analysis approaches [40–42]. These challenges make precise MS assessment devices stay at the exploratory stage.

Nowadays, deep learning (DL) methods have been rapidly developed [43–45]. Nervously controlled muscle stretching and extraction contain bioelectric and strain signals, which separately originated from nerve conduction and muscle’s physical change, possessing both time and frequency domain information [46,47]. DL methods, e.g., convolutional neural networks (CNNs) and long short-term memory networks (LSTMs), provide means for classifying and predicting such signals [48–50]. However, CNN is weak in long-term patterns capture [48,51], while the compression of gating mechanisms in LSTMs can result in information loss, leading to inaccurate classification [50,52,53]. In comparison, the temporal convolutional network (TCN) model provides a superior pathway [54]. However, its limitation lies in the inability to link muscle activity with signals and establish associations among multiple signals. Fortunately, the attention mechanism in Transformer offers an effective approach. By incorporating this mechanism with TCN as the feature extraction layer, it enables parallel attention to the global information within each signal channel and the interaction among different channels [55–57], facilitating better capture of the long-term dependencies of multidimensional sequences and the association of muscle activity with signals. This approach contributes to the decoupling and correlation of multimodal data, achieving a high performance of DL models.

Here, we present a wearable myoelectric-strain-coupled system that combines sEMG and strain sensor (termed Coupsensor) for synchronized detection of muscle activities, and employs a modified Transformer method for accurate grading of MS. The 3-low-impedance-gel-electrode sEMG, showing a high SNR of about 39.7 dB, and the interdigital-layout piezoelectric strain sensing unit are designed in the same horizontal plane, minimizing the spatiotemporal difference. The modified Tcnformer model with TCN, i.e., TCN + Transformer (named Tcnformer), with integrated multi-head attention mechanism, apparently enhances the correlation between the neural and mechanical signals, which leads to precise MS grading and prediction results. Furthermore, by combining TCN and deep cluster, we developed the Tcnformer deep cluster (TDC) classification method, which can automatically generate a more precise classification of MS strength with 25 fined levels than the existing 5-level classification for accurate clinical MS assessment. Then, we further provided quantification and validation to showcase a patient’s postoperative recovery, with levels increasing from 3.2 to 3.6 in the first few days after surgery. We anticipate that this system will importantly advance precise MS assessment, potentially improving relevant clinical diagnosis and rehabilitation outcomes.

## Results

### Wearable MS grading evaluation system

**Fig. 1.**
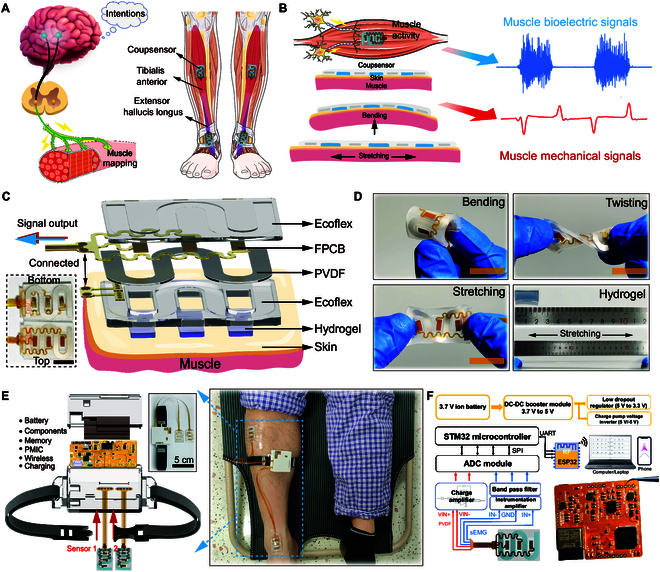
Coupsensor and the corresponding CMSAS. (A) Mechanism of sEMG signal generation and the Coupsensor attached to the belly of TA and EHL muscle for simultaneous retrieval of multiple muscle activities. (B) Coupsensor for synchronized retrieval of the sEMG signals generated during muscle activity and mechanical signals, i.e., muscle stretch, flexion, and contraction. (C) Exploded-view schematic illustrations of the Coupsensor (size of 32×24×2 mm), in which the serpentine piezoelectric film and 3 hydrogels are arranged in the same horizontal plane to ensure data synchronization. Scale bar, 1 cm. (D) Coupsensor enables stretching, bending, and twisting, with the stretchable hydrogel electrode shown in the lower right corner. Scale bar, 1 cm. (E) Exploded-view schematic diagram of the CMSAS (inset shows its optical image), clinical application scenarios, and (F) corresponding integrated circuit and system flow diagram.

The sEMG originates from the bioelectrical activity of spinal alpha motor neurons under the control of the brain’s motor cortex. It is activated by the summation of numerous muscle fibers unit potentials. The left side of Fig. 1A demonstrates the mechanism of sEMG signal generation under neural control, while the right image shows the non-invasive device Coupsensor attached to the belly of the tibialis anterior (TA) and extensor hallucis longus (EHL) muscle. During muscle excitation and contraction, the Coupsensor adhered to the skin epidermis can synchronously retrieve both sEMG signals and mechanical signals, such as stretching, bending, and contraction of muscles, as illustrated in Fig. 1B. To avoid asynchronism caused by spatial separation, we placed the piezoelectric sensor (polyvinylidene difluoride [PVDF], with a thicknesses of 100 μm) and 3 hydrogel electrodes in the same horizontal plane (Fig. S1), as depicted in the explosion schematic of the Coupsensor in Fig. 1C, in which the design of the electrodes on the PVDF film is detailed in Fig. S2. The serpentine FPCB and PVDF are encapsulated by Ecoflex in an inverted mold, enabling them to remain flexible. Optical photographs demonstrating the flexibility of the Coupsensor are shown in Fig. 1D. Furthermore, the tensile performance of the Coupsensor was examined (Fig. S3A), and the results revealed that its tensile strain exceeded 140%, fully meeting the stretchability requirements for our clinical testing. Meanwhile, stress simulation analyses of PVDF and Ecoflex materials demonstrated noticeable strain under a specified 4 N stress, including stretching and compressive force, as illustrated in Fig. S3B. The preparation process of the Coupsensor is depicted in Fig. S4 and described in Methods.

Due to the features of flexibility and stretchability of the Coupsensor, a wearable clinical MS assessment system (CMSAS) has been developed. The system, as depicted in the exploded schematic images shown in Fig. 1E, consists of 2 Coupsensors that are affixed to the skin using medical tape, enabling simultaneous, real-time detection of multiple muscles in parallel, i.e., TA and EHL muscles. Meanwhile, the system achieves a 4-channel, high sampling rate (1 K s^−1^) and integrated design by employing a high-speed analog-to-digital (AD) converter module, parallel Serial Peripheral Interface (SPI), and User Datagram Protocol wireless communication protocol (Fig. 1F). The system flow diagram and integrated circuit of the CMSAS are presented in Figs. S5 and S6.

### Gel electrode preparation and Coupsensor characterization

**Fig. 2.**
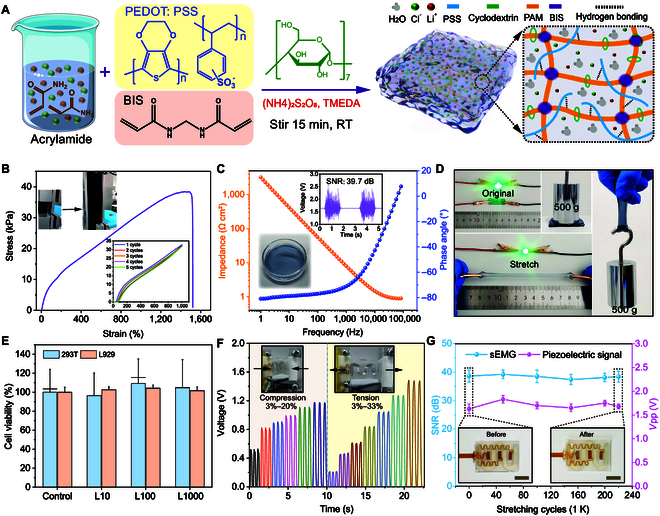
SIH preparation, characterization, and strain sensor simulation and characterization. (A) Schematic diagram of the SIH preparation process, highlighting the key steps involved in the synthesis and fabrication of the SIH. (B) Characterization of tensile strain and tensile cycling of the SIH. (C) Impedance curve of the SIH, with an inset photo image demonstrating its successful retrieval as an electrode for myoelectric signals, achieving an average SNR of 39.7 dB. (D) SIH as a conductive electrode, with no significant variation in the brightness of the LED light in the initial and stretched states, and able to withstand the weight of 500-g weights. (E) Cytocompatibility characterization of SIH with cell survival rates above 90% after 24-h incubation with the culture medium prepared from the hydrogel. (F) Characterization of the tensile properties of the serpentine piezoelectric sensor. (G) Stretchable durability characterization of the Coupsensor. After 220,000 continuous stretching cycles (at a speed of 1 m s^−1^ with a strain of ~31% and a length of ~1 cm), the Coupsensor exhibits outstanding stability and performance (each point represents the average of 10 values). The inset images illustrate the absence of noticeable damage to the device before and after the stretching; scale bar, 1 cm.

As illustrated in the preparation flow of Fig. 2A, we synthesized a stretchable ionic hydrogel (SIH) based on polypseudorotaxane architectures [58,59] to improve conductivity, reduce impedance, and achieve stretchability. The SIH was prepared by introducing dual crosslinkers, a host molecule, a conductive polymer, and an ionic solution. The detailed preparation process, the SEM cross-sectional structure, and the excellent adhesion and transparency of the SIH are provided in Methods and Figs. S7 and S8, respectively. Owing to the hydrogen bonding interactions and the polypseudorotaxane internal network, the SIH shows excellent stretchability. As demonstrated in Fig. 2B and Fig. S9, the SIH stretches up to 1,500%, remains stable under the repetitive strain of 1,000%, and is even able to withstand the 500-g weight. Furthermore, the AC impedance of the SIH was investigated (Fig. 2C), revealing an impedance of 57 Ω at 100 Hz, and the inset indicates that the average myoelectric SNR of 10 repeated measurements using the Coupsensor with the gel was 39.7 dB. Measurement of the SIH exhibited high conductivity compared to 5 commercial wet electrodes with a value of ~6 S m^−1^ (Fig. S10), and there was no significant variation in the brightness of a light-emitting diode (LED) between the initial and stretched states (Fig. 2D). Besides, we conducted a comprehensive characterization of 3 commercial gel electrodes, SIH electrodes, and the Coupsensor, as depicted in Fig. S11. The results indicate that under identical testing conditions with 5 healthy subjects, the Coupsensor outperforms commercial wet electrodes in sEMG signal quality.

Remarkably, even after enduring a 16-h attachment to the skin and being stored at low temperatures (−20 °C) for 2 days, the SIH maintained high conductivity (more than 2 S m^−1^ shown in Fig. S12). To assess the cytocompatibility of the SIH, L929 mouse fibroblast cells and 293T human embryonic kidney cells were cultured in the original solution diluted 10-fold, 100-fold, and 1,000-fold with Dulbecco’s Modified Eagle Medium (DMEM) high-glucose culture medium [60,61]. After 24 h (Fig. 2E) and 48 h of culture (Fig. S13), cell survival rates above 90% were observed, indicating excellent cytocompatibility of the SIH. Additionally, 2 different typical serpentine structures of PVDF were investigated, and the simulation results are shown in Fig. S14. It is found that the PVDF with structure 1 exhibited superior electrical output performance compared to that with structure 2 under the same stretch distance and the same polarization (the parameter of PVDF is shown in Table S1). Experimental validation demonstrated that structure 1 exhibits the capability to detect minute strains of 3% (tensile distance: ~1 mm) under both tension and compression (Fig. 2F), along with a precise press sensing of 0.02 N (Fig. S15). Therefore, PVDF of structure 1 was chosen for subsequent investigations, with its polarization direction along the thickness direction. It deserves attention that after 220,000 cycles of continuous stretching (1 cm stretch length, ~31% strain), the Coupsensor still exhibits excellent stability and outstanding performance (SNR > 30 dB, *V*pp > 1.5 V), with optical photographs showing no significant damage to the device before and after stretching.

### Bioelectric and mechanical signal retrieval

**Fig. 3.**
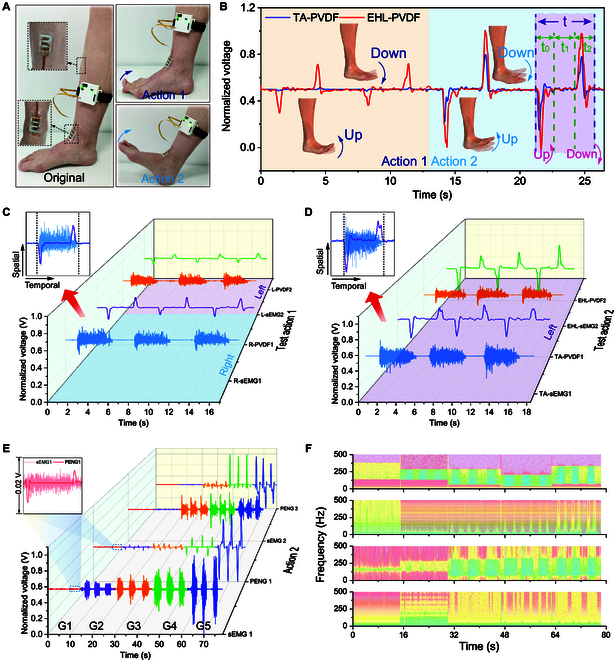
Coupsensor-based CMSAS for muscle activity retrieval. (A) Optical photographs of the Coupsensor attached to the EHL and TA muscles, depicting 2 sets of movements used to assess the MS grade of each muscle: action 1 (toe hook) and action 2 (ankle flexion). (B) Synchronous strain signals of the TA and EHL muscles during the 2 sets of movements, and the right schematic depicts the information about the association of strain signals with muscle mechanical activity. (C) Normalized sEMG and mechanically normalized signals of the subject’s right and left legs while performing action 1, with the inset showing the synchronization without phase difference between multiple channels for the right EHL muscle during a single movement. (D) Normalized curves of sEMG and mechanical signals obtained while performing action 2 on the left leg. The inset indicating the synchronization without phase difference between sEMG and strain signal. (E) Normalized sEMG and piezoelectric time domain curves (inserted magnified graph data represents Coupsensor testing of patients with MS grade 1) and (F) corresponding time–frequency domain information for 5 patients with different MS grades of TA muscle (grading from 1 to 5).

As depicted in Fig. 3A, a CMSAS was bound to the subject’s lower leg, with 2 sets of Coupsensors attached to the TA muscle belly and EHL muscle, respectively. To quantify the MS levels of these 2 muscles, the participant was instructed to perform 2 distinct actions. The first action involved toe flexion of the foot with maximum force, while the second action involved dorsiflexion and plantarflexion of the foot with maximum force; both actions do not require the patient to against resistance but should be sustained for 1 to 2 s before relaxing. The normalized mechanical signals associated with the 2 distinct movements are presented in Fig. 3B. The device adhered to the skin is subjected to a stretching force when the patient performs plantarflexion. Then, local stress concentration makes the PVDF equivalent to having a downward pressing force. Conversely, the PVDF shrinks when the patient performs dorsiflexion. Obviously, during action 1, the TA muscle experiences minimal mechanical strain (Movie S1), while both the TA and EHL muscles experience mechanical strain during action 2 (Movie S2). The mechanical signal is intricately linked to the onset, cessation, duration, and intensity of joint movement, thereby providing information regarding the velocity, dwell time, and amplitude of muscle mechanical activity. As shown in the right side of Fig. 3B, the time of the foot dorsiflexion (*t*_0_) and the foot plantarflexion (*t*_2_) describe how fast or slow the muscle activity rate is, while the amplitude responds to the intensity of the strain, and the pause time is determined by the time interval between the 2 peaks (*t*_1_). Additionally, the total duration of the signal (*t*) indicates the velocity of doing the whole set of movements. This characteristic information of strain signals can be effectively captured by DL algorithms and combined with myoelectric signals to analyze muscle function. More importantly, as depicted in Fig. S16, our developed sEMG and strain-coupled Coupsensor is flexible and stretchable, offering excellent comfort and skin adhesion. This property allows it to establish effective contact with the skin, making it superior to many small-sized commercial non-invasive sEMG sensors.

We investigated the impact of attachment locations on sEMG and strain sensors (Fig. S17). Results revealed that varying adhesion positions of myoelectric and strain sensors are easily susceptible to spatiotemporal differences and produce phase differences. In contrast, our coupled sEMG and strain sensor, placed in the same plane and space, mitigates this issue. The normalized sEMG and mechanical signals of the left and right legs during action 1 are presented in Fig. 3C (for unnormalized data, see Fig. S18), while the top left enlarged image describes the myoelectric and piezoelectric signals of the right EHL muscle during a single action. The Pearson correlation coefficient between the combined signals for the same action was ~0.981, indicating the high consistency of the actions during the test (more detailed information is provided in Table S3). In particular, the Coupsensor attached to the EHL muscle enabled synchronous collection of myoelectric and piezoelectric signals, mitigating potential discrepancies in data synchronization and detection that may arise from sensor separation. The approach facilitates the application of DL models to analyze signal feature parameters, temporal dependencies, and multichannel signal correlations. Figure 3D depicts the normalized test results of the subject’s sEMG and piezoelectric signals during action 2 with the left leg (for unnormalized data, see Fig. S14), where the correlation coefficients of both left and right legs are ~0.865 (test results of the right leg are presented in Fig. S19). Compared to action 1, the sEMG and piezoelectric signals measured during action 2 exhibited a larger amplitude, which was attributed to the greater muscle activity amplitude during action 2. Moreover, the top left corner of Fig. 3D indicates that the Coupsensor collected temporally and spatially consistent sEMG and piezoelectric signals of the TA muscle.

Furthermore, we conducted systematic testing on 33 patients with various MS function grades, ranging from grade 1 to 5, referring to the clinical 5-level MS grade criteria. These patients encompassed a diverse demographic profile, including different genders and ages spanning from 23 to 75 years. They were all recruited from the orthopedic ward and exhibited lower limb muscle weakness attributed to spine-related conditions. Detailed demographic and clinical information for all patients is provided in Table S2. It is noteworthy that during clinical testing, based on the clinical MMT standards, the final classification of patients’ MS function levels is determined collaboratively by 2 experienced clinical physicians. Only after a consensus is reached and the accuracy is ensured are the MS level results considered as the foundation for subsequent data training and prediction. Taking the MS test of the TA muscle as an example, Fig. 3E (time domain) and Fig. 3F (frequency domain) present the time–frequency domain data of the TA muscle for 5 patients with different MS levels (ranging from 1 to 5) via action 2 (similar results for the test of EHL muscle illustrated in Fig. S20). Significantly, as shown in Fig. S21, for patients with grade 1 MS, characterized by slight muscle contractions, Coupsensor demonstrates effective monitoring of these weak muscle activity. Accordingly, patients with different levels of MS exhibited significant differences in time–frequency domain features, training these multichannel signals during muscle activity by DL methods, providing a signification basis for MS grading or fine grading. Notably, the strong correlations exist between the 2 sets of signals generated by different patients with the same MS grade and by patients themselves when performing the same movements (see Tables S4 and S5). This contributes to enhancing the accuracy of MS grading.

### Tcnformer-based MS grading model

**Fig. 4.**
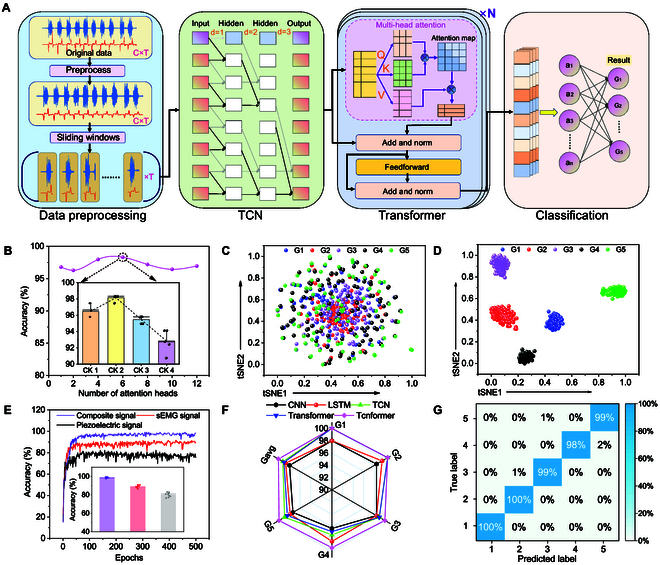
Tcnformer-based for MS classification. (A) Network architecture of the Tcnformer model, comprising data preprocessing, TCN, Transformer, and classification modules. (B) Investigation of the optimal Tcnformer configuration using the number of self-attentive heads (ranging from 1 to 12) and different dilation rate combinations, consisting of 1-1-1 (CK1), 1-2-3 (CK2), 1-2-5 (CK3), and 2-2-2 (CK4). Results demonstrate the highest accuracy achieved with 6 self-attentive heads and CK of 2. (C) t-SNE visualization plot of the original input, and (D) t-SNE visualization plot of the features after the Tcnformer module. (E) Exploration of the relationship between training epochs and accuracy of the Tcnformer-based model using myoelectric, piezoelectric, and coupled signals (myoelectric and piezoelectric coupling); the inset shows that the average accuracy of 10 times is ~98.3%. (F) Radar plot comparison of classification accuracy between Tcnformer and other DL models (CNN, LSTM, TCN, and Transformer) for each grade of MS. Tcnformer outperformed all other models, achieving an average accuracy of ~98.3%. (G) Tcnformer-based confusion probability matrix for patients with TA muscle grades ranging from 1 to 5, with a highest accuracy of about 99.2%.

Considering the distinctive features of sEMG and mechanical signals, we propose a DL network architecture, denoted as “Tcnformer”, which leverages TCNs and Transformers [62] for refined classification and prediction of MS grades. Figure 4A depicts the topology and data processing flow of the Tcnformer, which comprises 4 key modules, namely, the preprocessing module, the TCN module, the Transformer module, and the classification module. Taking the TA muscles’ multimodal signal data as an example, noise rejection and elimination of industrial frequency interference are performed on the raw muscle activity signal in the preprocessing module through using a band-pass filter from 10 to 450 Hz. The signal is then segmented using a sliding window approach with a window length of 200 samples and a step size of 100 samples (1K sample/s), followed by feeding the segmented data into the TCN module for hybrid null convolution and time–frequency domain feature extraction. Subsequently, the extracted feature parameters are assigned global attention and obtained feature information of dimension 560 by the multi-head attention mechanism of the Transformer. Finally, the Softmax-based classification module outputs the MS grades (ranging from 1 to 5) of the TA muscle.

We further investigated the impact of dilated convolutional kernel (CK) architectures of the TCN model with different dilation rate combinations, consisting of 1-1-1 (CK1), 1-2-3 (CK2), 1-2-5 (CK3), and 2-2-2 (CK4), and different numbers of attention heads in the Transformer model on the fitting ability of the end-to-end Tcnformer model. These findings are visually presented in Fig. S22 and summarized in Fig. 4B. It is worth noting that this analysis was conducted using a dataset of 32 patients for model training. Our results demonstrate that the accuracy of the Tcnformer can be optimized by increasing the number of self-attention heads up to 6 and using CK2 with different expansion rates (the detailed architecture of CK2 is depicted in Fig. S23). The number of attention heads and the architecture of CK are known to be crucial factors affecting the performance of the Tcnformer model, as demonstrated by the accuracy analysis. Similar results were observed in the data analysis of the EHL muscle, as presented in Fig. S24. The t-distributed stochastic neighbor embedding (t-SNE) has been widely adopted as a statistical downscaling and visualization technique. Therefore, we evaluated the effectiveness of the Tcnformer in feature separation by applying t-SNE. Figure 4C and D present the t-SNE visualization of the original input signal and the processed signal of TA muscle by the Tcnformer module, respectively (Fig. S25 provides a visualization of the EHL muscle data). The results demonstrate that the processed training data by the Tcnformer exhibits excellent interclass separability and intraclass compactness.

Besides, we investigated the impact of different signals (sEMG, piezoelectric, and muscle-piezoelectric composite signals) of the TA muscle and different DL networks on the model’s accuracy. As shown in Fig. 4E, owing to the incorporation of the multihead attention mechanism that enhances the correlation between single and multichannel signals, the average accuracy of 10 calculation results show that the coupled signals achieved higher predictive accuracy (~98.3%) compared to independent sEMG or piezoelectric signals. Figure 4F presents a comparison with different models, revealing that Tcnformer-based MS assessment at each grade and its average accuracy significantly surpass other DL network models (CNN, LSTM, TCN, and Transformer), with the confusion probability of the detailed 5-level MS assessment demonstrated in Fig. 4G. Similarly, we achieved outstanding outcomes for the training and MS grading of the EHL muscle data, with a grading accuracy of ~98.4%, as depicted in Fig. S26.

### Refinement of clinical MS grading

**Fig. 5.**
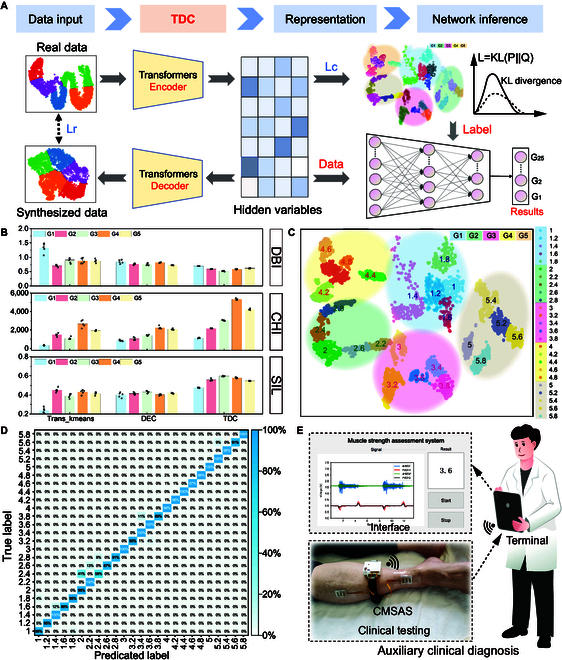
TDC-based MS refinement grading. (A) Schematic data process flow based on TDC architecture and MS refinement grading. (B) Evaluation of TDC performance using unsupervised clustering performance evaluation metrics (DBI, CHI, and SIL) based on patient data with TA muscle grades ranging from 1 to 5. (C) TDC model implementation of the characteristic discrete point distribution for 25 levels of TA refinement grading, and (D) corresponding detailed confusion matrix (range from 0% to 100%) with an accuracy rate of ~95%. (E) Conceptual schematic of the CMSAS demonstrating the results of first-day postoperative grade 3.2 in patients with TA muscle and a refined fourth-day postoperative grade 3.6 prediction.

To advance the current 5-level MS assessment system and draw inspiration from transfer learning [63,64] based on data from 32 patients, we propose an innovative unsupervised deep clustering method [65,66], termed TDC, for clinical MS refinement grading. The working principle of TDC is illustrated in Fig. 5A and involves the following steps: (a) constructing a self-encoder by employing the Tcnformer model and the fully connected layer as the encoder and decoder, respectively, to acquire an effective feature representation of the hidden layer; (b) fine-tuning the self-encoder using the reconstruction loss (Lr) function to obtain low-dimensional hidden layer features with strong differentiation ability; (c) discretizing the hidden variable *z* using the clustering loss (Kullback–Leibler [KL] divergence) to enable the hidden space data to achieve better interclass separability and intraclass compactness; (d) implementing unsupervised refinement classification of the original 5-level signal based on the trained TDC model and obtaining refinement 25-class labels; and (e) fixing the feature extraction layer of the Tcnformer model, replacing the classification layer with 25-class output, and fine-tuning the model using the refinement labels to finally obtain the muscle refinement 25 classification model. The evaluation of TDC can effectively reflect the performance of the model, and the Davies–Bouldin Index (DBI), the Calinski–Harabasz Index (CHI), and the Silhouette coefficient (SIL) are of interest as important metrics for unsupervised clustering performance evaluation [67–69]. Accordingly, we evaluate the performance of TDC under these 3 metrics using patient data with TA muscle grades ranging from 1 to 5. The results are presented in Fig. 5B. It is evident from the results that TDC outperforms Trans-kmeans (combination of Transformer and K-means) and Deep Embedded Cluster (DEC) methods. Similar tests on EHL muscles, as shown in Fig. S27, reflect the advantages of TDC. More details of the model’s sustainability, computational capability, and data processing are shown in Methods.

We further investigated the performance of TDC using sEMG and strain signal data from the TA muscle from 30 patients. Specifically, we analyzed the discrete points and confusion matrix of the 25-level TA muscle refinement grading features under the TDC model, as shown in Fig. 5C and D, respectively. The results demonstrate that TDC effectively separates each small grade of the 5 grades of MS, with minimal overlap between sub-grades. The confusion matrix of the 25 grades of TA muscle has an impressive accuracy of about 95%, further validating the efficacy of TDC. Similar results were obtained for the finely graded EHL muscle, as illustrated by the discrete points and confusion matrix shown in Figs. S28 and S29, respectively, with an accuracy rate of nearly 92%.

Building upon the strengths of TDC and flexibly coupled sensing, we developed a CMSAS to accurately predict preoperative and postoperative MS of patients with easy data access and input. Figure 5E depicts a demonstration case of the CMSAS. It is found that a patient on the first postoperative day has a TA muscle grading of 3.2, and on the fourth postoperative day, the grade turned to 3.6, quantifying his recovery (further details shown in Movie S3). Moreover, for 2 patients, A (17) and B (22), with similar MS levels but differing resistance capabilities, CMSAS effectively distinguishes the differences in their MS levels. Algorithmic assessment classifies both patients as grade 4 in the MS classification, but patient B exhibits stronger resistance capabilities (4.6 > 4.0). Their clinical presentations align with the MS classification assessment results, as detailed in Table S6 on MS refinement grading and depicted in Fig. S30. Overall, our results indicate that TDC and the CMSAS hold great promise for accurate and efficient MS assessment.

## Discussion

In this work, we introduce a wearable Coupsensor capable of capturing bioelectric and strain signals synchronously, along with a DL analysis model for MS detection and assessment. The Coupsensor’s SIH gel ensures excellent signal conductivity and low impedance, resulting in an average SNR of ~39.7 dB, and the same-plane layout ensures data synchronization, minimizing spatiotemporal differences. The novel developed Tcnformer-based DL model efficiently focuses on features and long-time dependencies in the signals, enhancing the correlation between multichannel signals and achieving higher MS grading and prediction. Further, we developed a data classification method based on the TDC model that led to a refined 25-level classification of MS. We tested the feasibility of our system by evaluating postoperative patients, demonstrating that our approach provided a more digital and precision assessment of MS compared to the traditional 5-level clinical MS grading system.

In short, the multimodal data acquisition and sensing approach we propose is applicable to various scenarios in healthcare data collection. Meanwhile, the developed DL-based multimodal data correlation and decoupling strategy enable more comprehensive analysis and processing of healthcare data, with potential applications in similar data analysis tasks, opening new avenues for precision healthcare and intelligent health assessment. Nevertheless, we acknowledge the necessity for further considerations, including the collection of more data samples, exploration of variations in MS decline caused by different diseases, and clinical validation across diverse populations to delve deeper into the system’s stability, applicability, and effectiveness. Subsequent research endeavors may entail deeper investigations to extend its applicability to broader clinical domains.

## Methods

### The preparation process of the SIH

The SIH was synthesized by introducing acrylamide (AM) and *N*,*N*′-methylene bisacrylamide (BIS) as dual crosslinkers, β-cyclodextrin (β-CD) as a host molecule, poly(3,4-ethylenedioxythiophene):poly(styrene sulfonate) (PEDOT:PSS) conductive polymer, and LiCl ionic solution, with tetramethylethylenediamine (TMEDA) as a catalyst for polymerization. The β-CD molecules were threaded onto the formed polyacrylamide (PAM) chains, forming a polypseudorotaxane architecture internal to the SIH. The detailed steps are as follows: 6 g of AM solid particles and 5 g of LiCl particles were dissolved in 19.5 ml of deionized water with continuous magnetic stirring at a rotational speed of 300 r min^−1^, until a homogeneous aqueous solution was obtained. Subsequently, 0.5 g of β-CD, 0.005 g of BIS, and 5 ml of PEDOT:PSS solution (conductivity 700 to 800 S cm^−1^) were added to the solution, followed by 0.05 g of ammonium persulfate (APS). The reaction solution was stirred for 15 min at room temperature to ensure complete dissolution of the added materials. The resulting solution was introduced into a prefabricated glass mold, and 20 μl of the TMEDA catalyst was added dropwise under homogeneous stirring. This led to a self-polymerization chemical exothermic reaction, forming a polypseudorotaxane architecture and producing the stretchable viscous ionic hydrogel.

### The preparation process of the Coupsensor

The Coupsensor was fabricated as follows. Initially, 2 distinct serpentine patterns, FPCB1 and FPCB2, were designed using the Altium Designer 18 software. The PVDF film was then laser-cut to obtain a matching-sized serpentine piezoelectric film. Subsequently, a uniform application of conductive silver paste onto the upper and lower surfaces of the serpentine PVDF film was achieved using a mask plate, creating the stretchable strain sensor. The serpentine FPCB1 and PVDF film were then securely bonded and fixed together using a conductive copper foil. To establish interconnection, FPCB1 was connected to FPCB2 through conductive hole mapping. The composite materials, including the aforementioned components and FPCB2, were then placed within a specially designed 3D encapsulation mold. Subsequently, silica gel was poured into the mold, fully encapsulating the materials. Following complete curing of the silica gel, the mold was removed, resulting in the production of the Coupsensor. For more detailed information regarding the specific sensor preparation process and the interconnection of the 2 sets of the Coupsensor, please refer to Fig. S4.

### Integrated circuit design and fabrication

The myoelectric and piezoelectric coupled acquisition circuit boards are integrated and modularized to ensure stable sensing performance (circuit size of 4.5 cm × 5 cm). Four-layer design of the multi-channel acquisition circuit board comprises charge amplification module, myoelectricity acquisition module, power management integrated circuit (PMIC), wireless communication module, main control module, and AD conversion module. To minimize mutual crosstalk between signals, the 2 sets of charge amplification modules, instrument amplification module and myoelectric acquisition module, are analog ground isolated. The piezoelectric outputs of the 2 Coupsensor sensors are connected to the 2 independent inputs of the charge amplifier module, while the electrodes of the myoelectric sensing unit are connected to the instrumentation amplifier and band-pass filter for synchronous myoelectric acquisition and signal filtering. In addition, the detailed amplification gain of the myoelectric module is shown in Fig. S31. To prevent distortion in the acquisition of sEMG signals, we implemented voltage division by employing 2 10-KΩ resistors at the signal input terminals of the OPA333 operational amplifier. This action effectively maintained the baseline voltage of the sEMG signal at approximately half of the power supply voltage (~1.65 V). Simultaneously, the acquisition hardware for piezoelectric signals ensured that their reference voltage remained around 0 V. The acquired piezoelectric and sEMG signals were input to the high-speed AD conversion module (AD7606) for digitization during the SPI clock, with a sampling rate of 1 K s^−1^. Subsequently, under the control of the master control unit (STM32L431), the 4 sets of digitized sensing signals after AD conversion were sent to the ESP32 module via the Universal Asynchronous Receiver/Transmitter, and the data were wirelessly transmitted to the user terminal simultaneously (LabVIEW-based upper computer acquisition system). The principal flow of the integrated circuit board and LabVIEW acquisition waveform interface is presented in Figs. S4 to S6. The circuit board software development was carried out using Keil vision and the board was debugged using an oscilloscope (MSO8204, RIGOL Technologies, Co. Ltd.).

### Cytotoxicity tests

L929 mouse fibroblasts and human embryonic kidney 293T cells were cultured in DMEM with high sugar, supplemented with 10% fetal calf serum and 1% penicillin/streptomycin, at 37 °C with 5% CO_2_ until logarithmic growth phase was reached. Prior to the cytotoxicity tests, 0.971 g of polyacrylamide hydrogel containing LiCl was soaked in 30 ml of DMEM for 72 h. Then, the medium was diluted 10, 100, and 1,000 times. After washing with phosphate-buffered saline (PBS), trypsin digestion, centrifugation, DMEM resuspension, and cell counting plate counting, L929 and 293T cells were seeded into 96-well plates in sequential order and were cultured at 37 °C with 5% CO_2_ for 24 and 48 h, respectively. The density of seeded plates was 10^4^ and 6.7×10^3^ cells per 100 μl of culture medium, respectively. The culture medium was then washed twice with PBS, and 110 μl of the CCK8 mixture (10 μl of CCK8 and 100 μl of culture medium) was added. After incubation for 1 to 4 h, the absorbance at 450 nm was measured using enzyme-linked immunosorbent assay, and the results were calculated.

### Characterization of the SIH and the Coupsensor

The mechanical properties of the SIH were evaluated by conducting tensile strength and tensile cycling experiments using the Echolink YL-S70. The hydrogels were analyzed by SEM and energy-dispersive spectrometry (EDS) after freeze-drying in liquid nitrogen, using FEI/Nova NanoSEM 450. The conductivity and impedance analysis of the SIH were performed using the electrochemical workstation CHI760E. Transparency of the hydrogels was characterized using Agilent’s 3500 equipment. The PVDF-based piezoelectric sensor was engraved in a snake shape using a laser cutter machine (PLS6.75). The tensile and force testing of serpentine PVDF piezoelectric sensors was conducted using a linear motor (Linmot E1200) and a commercial tensiometer (Edberg SH-10 N).

### Simulation analysis and numerical calculations

For simulation analysis and numerical calculations, the structures of the 2 serpentine piezoelectric sensors were drawn by CAD 2020 and imported into COMSOL 5.4 software (the parameters of piezoelectric films are shown in Table S1). The electrostatic module of COMSOL 5.4 was selected to study the piezoelectric potential output at its steady state with a specified tensile displacement. To investigate the SNR of sEMG signals, the average voltage effective amplitude (*V*_S_) of myoelectricity during muscle activity (including excitation and contraction) and the average voltage effective amplitude (*V*_n_) of myoelectricity signals at rest were obtained using MATLAB 2020. The SNR of myoelectricity signals was then calculated using the following formula:

$$\text{SNR}=20\text{log}\frac{V_{s}}{V_{n}}$$ (1)

### Clinical MS data acquisition

Two professional clinicians diagnosed and graded the clinical MS of the patient by the MMT. Thereafter, the patient wore a DL evaluation system and performed the toe and ankle flexion movements with maximum effort to collect the sEMG and strain signals of the TA and EHL muscles simultaneously under the 2 movements mentioned above, respectively. The statistical tables of patients with different MS grades of TA and EHL muscles are shown in Table S2, and these data served as the basis for training and grading the DL network model. Moreover, the same patients with preoperative and postoperative changes in MS grade were followed up to verify the feasibility of the refined model.

### Preprocessing of data

To preprocess the data, a 10- to 450-Hz band-pass filter was applied to the TA muscle data (EHL muscle data was processed similarly) to reduce noise. The result values were then min–max normalized to map each channel to the interval range of [0,1]. This normalization process not only accelerates the learning speed of gradient descent but also improves the accuracy of recognition. Furthermore, for each patient-collected dataset, the corresponding dataset was normalized using the minimum and maximum values. All patient datasets’ minimum and maximum values were used in real time user testing scenarios to normalize the user signal inputs. The normalization calculation was performed as follows: for each patient, the original data *R* = {*r_i_*, *i*∈*N*}, and corresponding normalized data as *S* = {*s_i_*∈[0, 1], *i*∈*N*}.

$$s_{i}=\frac{r_{i}-\text{min}\left(R\right)}{\text{max}\left(R\right)-\text{min}\left(R\right)},\forall r_{i}\in R$$ (2)

### Tcnformer-based data processing

(a) Data allocation: there are overall 33 patients enrolled in our experiments. Data from 30 patients are used for training and validation (excluding patients 17, 22, and 33). Before that, these data were further split to about 600 samples, ranging from 1 to 5 levels, to facilitate data augmentation. Afterwards, a 4:1 ratio was used to perform 10-fold cross-validation on the training and validation data for model training. (b) Data processing: The TCN module employs a one-dimensional convolutional network architecture to process temporal data. To improve its temporal modeling capability and capture longer dependencies, we adopt a stacked convolutional architecture, comprising 3 convolutional layers, each with expansion factors of 1, 2, and 3, and with 40 convolutional filters of size 5. Furthermore, a further pooling layer of size 40 is linked to smooth the temporal features. The obtained feature representation *X* is then input to the Transformer framework.

In the Transformer framework, we use 3 fully connected layers to map the output X of the TCN module into 3 vectors of the same shape: query (*Q*), key (*K*), and value (*V*). The matrix multiplication operation allows for parallel computation, significantly increasing the encoding speed of continuous signals.

$$V=W_{V}X,\;Q=W_{Q}X,\;K=W_{K}X$$ (3)

We compute the dot product of each query vector *Q* with all key vectors *K* and perform softmax normalization to obtain a final representation that contains information about the entire input sequence. For each query vector *Q*, we compute its dot product with all key vectors *K* and perform softmax normalization. The final representation contains information about the entire input sequence by computing the weighted sum of query vector *Q* and overall value vector *V*. Finally, 2 fully connected feedforward layers are linked at the output to enhance the fitting ability. The expression of the computational procedure of this step is as follows:

$$\text{Attention}\left(Q,K,V\right)=\text{Softmax}\left(\frac{QK^{T}}{\sqrt{k}}\right)V$$ (4)

where *k* denotes the signal length (200). To improve the model’s ability to encode temporal signals, we repeat the above attention mechanism 6 times by stacking attention blocks, allowing the model to learn different patterns. After encoding the signals, the vectors obtained from each head are concatenated to generate a potential vector representing the entire signal sequence.

$$\begin{array}{c} \text{MultiHead}\left(Q,K,V\right)=\\ \text{Concat}\left(\text{Attention}\left(QW_{1}^{Q}KW_{1}^{K}VW_{1}^{V}\right),\ldots ,\text{Attention}\left(QW_{6}^{Q},KW_{6}^{K},VW_{6}^{V}\right)\right)W^{O} \end{array}$$ (5)

Finally, the obtained sequence potential vectors are fed into the classification module, which comprises a 256-unit fully connected layer, a 64-unit fully connected layer, and a 5-unit output layer (representing levels 1 to 5). The Tcnformer model employs the categorical cross-entropy function as the loss function, with a batch size of 72, 500 epochs, and an adaptive moment estimation (Adam) optimizer with a learning rate of 0.0001. To ensure regularization, we apply batch normalization and a dropout rate of 0.5.

### Unsupervised deep clustering based on transfer learning

We employed a combination of the model’s reconstruction loss and clustering loss to optimize the clustering results. The reconstruction loss was used to measure the difference between the original data and the self-encoder reconstructed data, whereas the clustering loss was applied to measure the difference between the distribution of clustering results and the target distribution. We devised a self-learning clustering method that leveraged the potential embedding representation of the Tcnformer Encoder to achieve superior clustering performance. The loss function took the form of KL divergence.

$$L=\text{KL}\left(P∥Q\right)=\sum_{i}\;\sum_{j}\;p_{\mathrm{ij}}\text{log}\frac{p_{\mathrm{ij}}}{q_{\mathrm{ij}}}$$ (6)

Specifically, *q_ij_* represents the soft label of the intermediate hidden variable *z*, while *z* denotes the representation space of the original feature distribution after the encoder. This variable could be interpreted as the probability that sample *i* belonged to cluster *j*. We calculated the similarity between *z_i_* and the cluster center *μ_j_* using the student distribution, which was described using the following equation:

$$q_{\mathrm{ij}}=\frac{{\left(1+{∥z_{i}-\mu_{j}∥}^{2}/\alpha \right)}^{-\frac{\alpha +1}{2}}}{\sum_{j^{\prime }}\;{\left(1+{∥z_{i}-\mu_{j^{\prime }}∥}^{2}/\alpha \right)}^{-\frac{\alpha +1}{2}}}$$ (7)

To obtain the initial clustering centers *μ_j_*, we performed K-means clustering based on the data distribution and selected the optimal results for training. Additionally, *p_ij_* was an auxiliary target distribution that was used to enhance the confidence level of *q_ij_*, which was expressed using the following equation:

$$p_{\mathrm{ij}}=\frac{q_{\mathrm{ij}}^{2}/\sum_{i}\;q_{\mathrm{ij}}}{\sum_{k}\;\left(q_{\mathrm{ik}}^{2}/\sum_{i}\;q_{\mathrm{ik}}\right)}$$ (8)

The reconstruction error of the model is obtained using the following formula to represent the average of the reconstruction loss of all samples. Minimizing this loss function allows the Encoder and Decoder to learn a better representation of the data and improves the clustering performance.

$$L_{\mathrm{rec}}=\frac{1}{N}\sum_{i=1}\;N{∥x_{i}-{\hat{x}}_{i}∥}^{2}$$ (9)

In the above equation, *x_i_* denotes the *i*th sample in the input data, ${\hat{x}}_{i}$ denotes the reconstruction result of that sample, and *N* denotes the total number of samples. The final model consisted of the self-encoder and the clustering part based on the fine-tuning of the Tcnformer model, respectively. Therefore, the objective function of TDC was expressed as:

$$L=\sum_{x}\;L_{\text{rec}}\left(x\right)+\lambda L_{\text{clust}}\left(x\right)$$ (10)

where *L*_rec_ and *L*_clust_ represent the KL loss functions based on the reconstruction error and clustering loss of the Tcnformer self-encoder, respectively, and λ > 0 was the coefficient controlling the relative weights of the 2 loss functions. In this study, we set λ to 0.1.

### Unsupervised clustering metrics

All clustering results are measured by DBI, CHI, and SIL. The DBI measures the mean of the maximum similarity of each cluster and can be expressed as:

$$\mathrm{DBI}=\frac{1}{k}\sum_{i=1}^{k}\;\underset{i\neq j}{\text{max}}\;\left[\frac{s_{i}+s_{j}}{d\left(c_{i},c_{j}\right)}\right]$$ (11)

where *k* is the number of clusters, *s_i_* is the average distance from the sample within the *i*th cluster to the cluster center, *c_i_* is the centroid of the *i*th cluster, and *d* (*c_i_*, *c_j_*) is the Euclidean distance between the cluster centers. A smaller DBI value indicates a better clustering effect as the clustering result is closer to the interior of the cluster. CHI considers both the tightness within clusters and the separation between different clusters, and a higher CHI value indicates better model performance. For a dataset *E* of size *n_E_* with *K* classes, the CHI index is defined as:

CHI=tBktWk×nE−kk−1Wk=∑q=1k∑x∈Cqx−cqx−cqTBk=∑q=1knqcq−cEcq−cqT (12)

where *t* (*B_k_*) is the trace of the inter-cluster discrete matrix, *t* (*W_k_*) is the trace of the intra-cluster discrete matrix, *C_q_* denotes the class *q* in which the current point is located, *C_q_* is the cluster centroid of the current class *q*, *C_E_* denotes the center of class *E*, *n_q_* is the number of points in *q* class, and *T* represents the matrix transformation. The SIL is a measure of how similar an object is to its own cluster compared to other clusters, and ranges from 0 to 1, with a higher value indicating a better clustering result. The formula for calculating the SIL is as follows:

$$\text{SIL}=\frac{b-a}{\text{max}\left(a,b\right)}$$ (13)

where *a* is the average intra-cluster distance and *b* is the average nearest-cluster distance of each sample.

### Model sustainability

The training dataset employed in this study comprises 597 activity segments, with each signal containing 4 channels and 200 data points per segment. This data scale adequately fulfills the requirements for model training while ensuring the reliability of results. The Tcnformer model employed in this study has 167,781 trainable parameters, while the TDC model has 299,601 trainable parameters. Given the relatively small size of the training data and model parameters, their energy consumption and impact on power networks and carbon emissions are limited. To address potential increases in parameter size in the future, techniques such as model pruning and quantization can be appropriately utilized to further reduce resource consumption.

### Code availability

The code for the Tcnformer and TDC models mentioned in the article is publicly available on Zenodo (https://doi.org/10.5281/zenodo.8361754). The firmware for the electronic hardware, custom-developed and written in C, and the source code for the computer software, custom-developed in LabVIEW, are available from the authors for research purposes on reasonable request.

## Data Availability

The main data supporting the results of this study are available within the paper and its Supplementary Materials. The raw and analyzed datasets generated during the study are too large to be publicly shared, yet they are available for research purposes from the corresponding authors at reasonable request. Source data are provided in this paper.
