# Supplementary material for: Deep Learning Model Coupling Wearable Bioelectric and Mechanical Sensors for Refined Muscle Strength Assessment
Source: Research (Wash D C). 2024 May 23;7:0366. doi: 10.34133/research.0366 (PMC11112600; doi:10.34133/research.0366)

a

Gel Electrodes List	Model	Manufacturer	Size
Vitrode	L-150X	Nihon Kohden	35 mm dia
3M 1	Red Dot™ 2228	3M Company	33 mm x 40 mm
3M 2	Red Dot™ 2223	3M Company	43.1 mm dia
Lepu 1	V0014A	LEPU Company	60 mm dia
Lepu 2	HW03	LEPU Company	60 mm dia
**This work**	---	---	**Customizable**

b

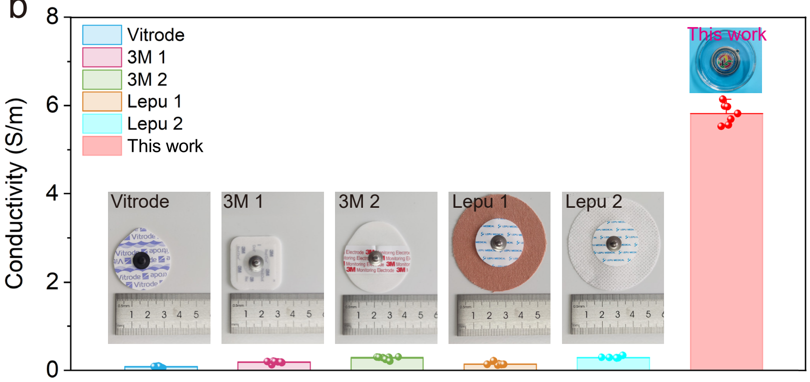

Supplement: Supplementary 1 — Figs. S1 to S31 Movies S1 to S3 Tables S1 to S6 [file research.0366.f1.zip › SI Figure/Fig. S10.pdf]
