# Supplementary material for: Deep Learning Model Coupling Wearable Bioelectric and Mechanical Sensors for Refined Muscle Strength Assessment
Source: Research (Wash D C). 2024 May 23;7:0366. doi: 10.34133/research.0366 (PMC11112600; doi:10.34133/research.0366)
Supplement: Supplementary 1 — Figs. S1 to S31 Movies S1 to S3 Tables S1 to S6 [file research.0366.f1.zip › SI Figure/Fig. S16.pdf]

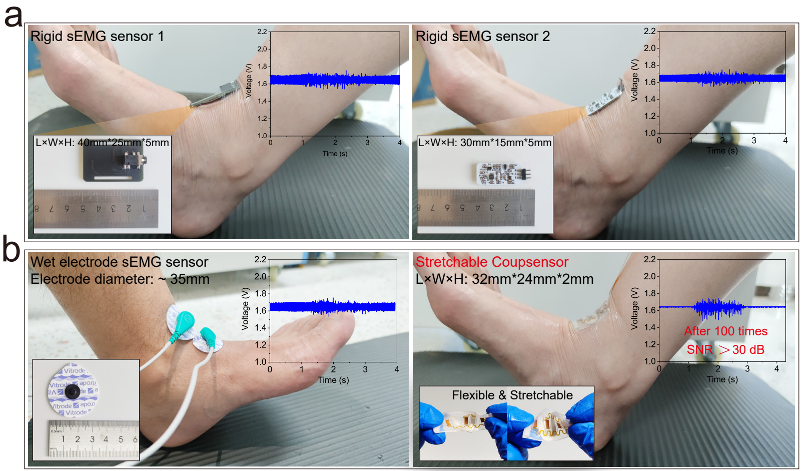
a
Rigid sEMG sensor 1
L×W×H: 40mm*25mm*5mm
Rigid sEMG sensor 2
L×W×H: 30mm*15mm*5mm
Voltage (V)
Time (s)
b
Wet electrode sEMG sensor
Electrode diameter: ~ 35mm
Vitrode
Stretchable Coupsensor
L×W×H: 32mm*24mm*2mm
After 100 times
SNR ＞30 dB
Flexible & Stretchable
