# Supplementary material for: Deep Learning Model Coupling Wearable Bioelectric and Mechanical Sensors for Refined Muscle Strength Assessment
Source: Research (Wash D C). 2024 May 23;7:0366. doi: 10.34133/research.0366 (PMC11112600; doi:10.34133/research.0366)
Supplement: Supplementary 1 — Figs. S1 to S31 Movies S1 to S3 Tables S1 to S6 [file research.0366.f1.zip › SI Figure/Fig. S22.pdf]

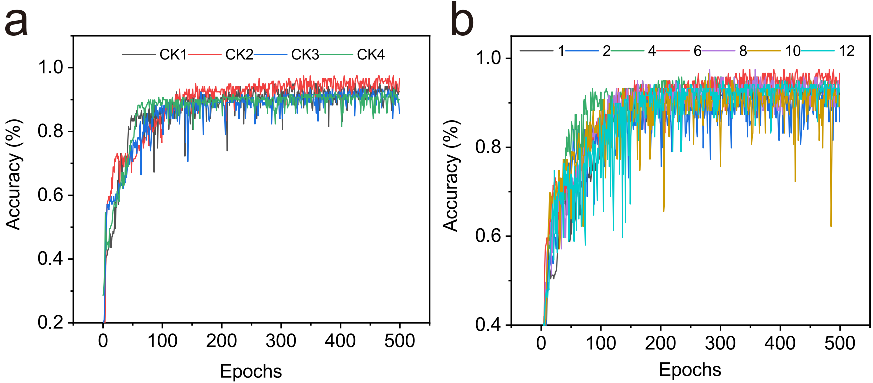

a
CK1
CK2
CK3
CK4
Accuracy (%)
Epochs
0.2
0.4
0.6
0.8
1.0
0
100
200
300
400
500
b
1
2
4
6
8
10
12
Accuracy (%)
Epochs
0.4
0.6
0.8
1.0
0
100
200
300
400
500
