# Supplementary material for: Deep Learning Model Coupling Wearable Bioelectric and Mechanical Sensors for Refined Muscle Strength Assessment
Source: Research (Wash D C). 2024 May 23;7:0366. doi: 10.34133/research.0366 (PMC11112600; doi:10.34133/research.0366)
Supplement: Supplementary 1 — Figs. S1 to S31 Movies S1 to S3 Tables S1 to S6 [file research.0366.f1.zip › SI Figure/Fig. S30.pdf]

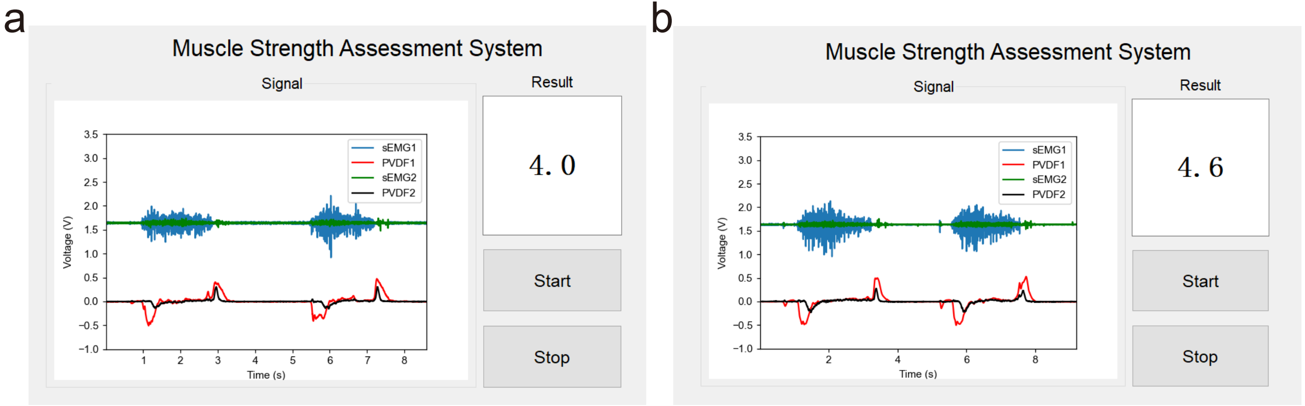
a
Muscle Strength Assessment System
Signal
Result
4. 0
Start
Stop
sEMG1
PVDF1
sEMG2
PVDF2
Voltage (V)
Time (s)
b
Muscle Strength Assessment System
Signal
Result
4. 6
Start
Stop
sEMG1
PVDF1
sEMG2
PVDF2
Voltage (V)
Time (s)
