# Supplementary material for: Deep Learning Model Coupling Wearable Bioelectric and Mechanical Sensors for Refined Muscle Strength Assessment
Source: Research (Wash D C). 2024 May 23;7:0366. doi: 10.34133/research.0366 (PMC11112600; doi:10.34133/research.0366)
Supplement: Supplementary 1 — Figs. S1 to S31 Movies S1 to S3 Tables S1 to S6 [file research.0366.f1.zip › SI Figure/Fig. S5.pdf]

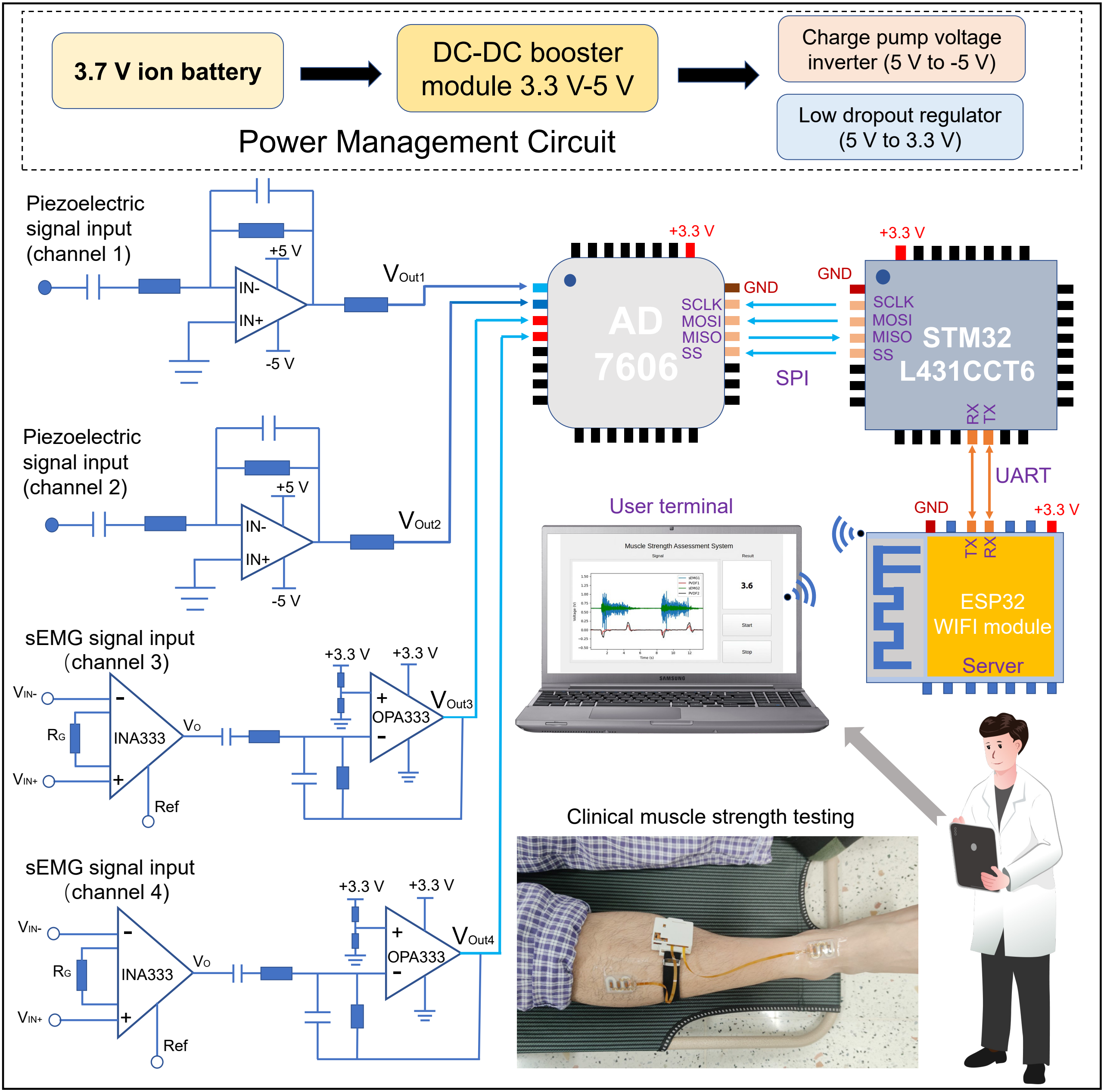

3.7 V ion battery
DC-DC booster module 3.3 V-5 V
Charge pump voltage inverter (5 V to -5 V)
Low dropout regulator (5 V to 3.3 V)
Power Management Circuit
Piezoelectric signal input (channel 1)
+5 V
IN-
IN+
-5 V
VOut1
Piezoelectric signal input (channel 2)
+5 V
IN-
IN+
-5 V
VOut2
sEMG signal input (channel 3)
VIN-
RG
VIN+
INA333
VO
Ref
+3.3 V
+3.3 V
OPA333
VOut3
sEMG signal input (channel 4)
VIN-
RG
VIN+
INA333
VO
Ref
+3.3 V
+3.3 V
OPA333
VOut4
+3.3 V
AD 7606
GND
SCLK
MOSI
MISO
SS
SPI
+3.3 V
GND
STM32 L431CCT6
RX
TX
UART
GND
+3.3 V
TX
RX
ESP32 WIFI module
Server
User terminal
Muscle Strength Assessment System
Signal
Result
3.6
Start
Stop
SAMSUNG
Clinical muscle strength testing
