# Supplementary material for: Deep Learning Model Coupling Wearable Bioelectric and Mechanical Sensors for Refined Muscle Strength Assessment
Source: Research (Wash D C). 2024 May 23;7:0366. doi: 10.34133/research.0366 (PMC11112600; doi:10.34133/research.0366)
Supplement: Supplementary 1 — Figs. S1 to S31 Movies S1 to S3 Tables S1 to S6 [file research.0366.f1.zip › Supporting information-0402.docx]

**Supplementary Materials for**

**Deep-learning model coupling wearable bioelectric and mechanical sensors for refined muscle strength assessment**

Chengyu Li^1,2,8^, Tingyu Wang^1,2,8^, Siyu Zhou^3,4,5,8^, Yanshuo Sun^1,2^, Zijie Xu^1,2^, Shuxing Xu^1,2^, Sheng Shu^1,2^, Yi Zhao^3,4,5^, Bing Jiang^1,7^, Shiwang Xie^1,2^, Zhuoran Sun^3,4,5^, Xiaowei Xu^6^, Weishi Li^3,4,5,*^, Baodong Chen^1,2^ and Wei Tang^1,2,7,*^

1 Beijing Institute of Nanoenergy and Nanosystems, Chinese Academy of Sciences, Beijing 101400, China

2 School of Nanoscience and Technology, University of Chinese Academy of Sciences, Beijing 100049, China

3 Department of Orthopaedics, Peking University Third Hospital, Beijing 100191, China

4 Engineering Research Center of Bone and Joint Precision Medicine Ministry of Education

5 Beijing Key Laboratory of Spinal Disease Research

6 Guangdong Provincial People’s Hospital, Guangdong Academy of Medical Sciences, Guangzhou, China

7 Center on Nanoenergy Research, School of Physical Science and Technology, Guangxi University, Nanning 530004, China

8 Chengyu Li, Tingyu Wang and Siyu Zhou contributed equally to this work.

*e-mail: [puh3liweishi@bjmu.edu.cn](mailto:puh3liweishi@bjmu.edu.cn); tangwei@binn.cas.cn


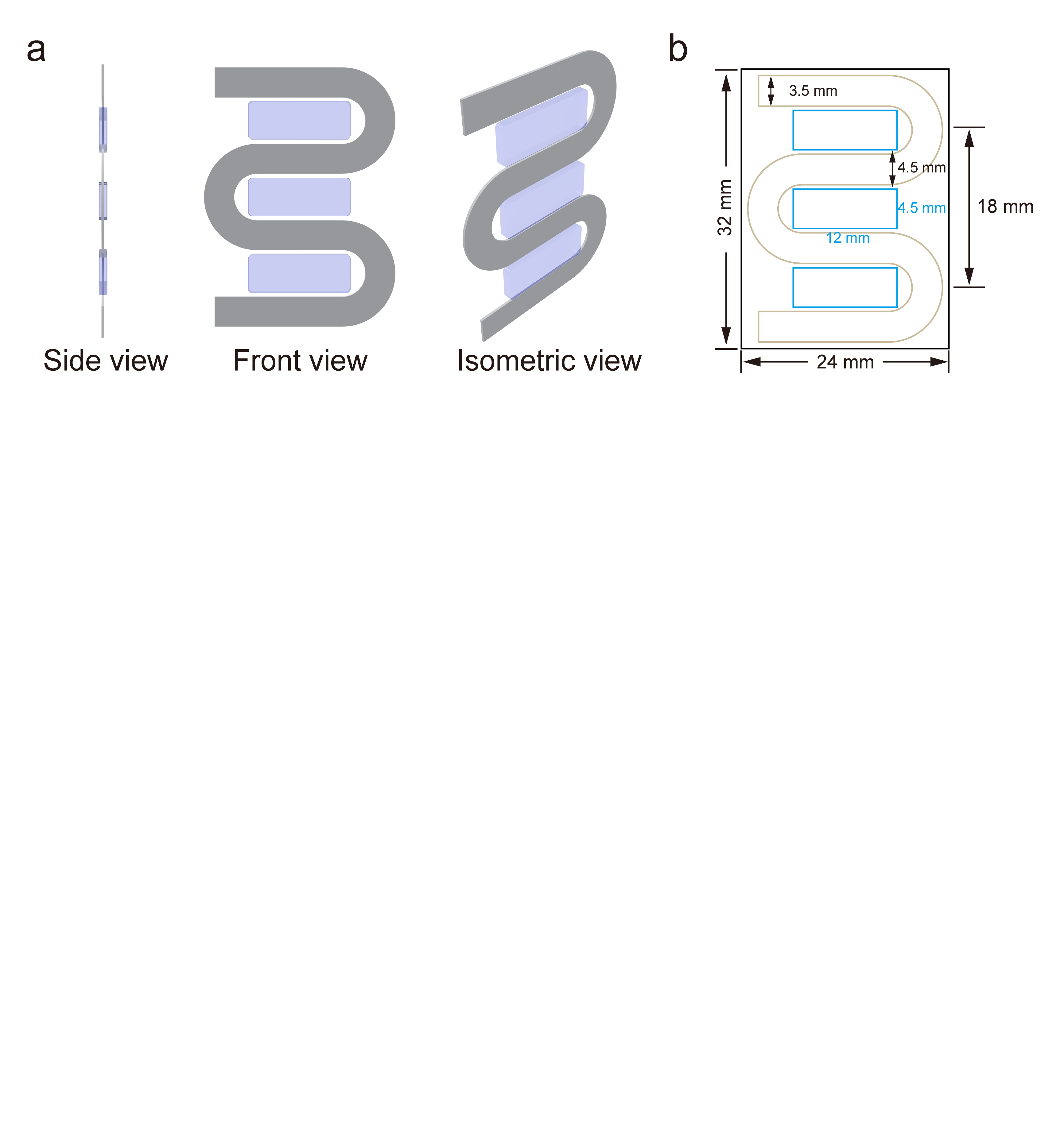


**Supplementary Fig. 1.** Layout and dimensions of the Coupsensor. **a**, Schematic representation of the Coupsensor, providing multiple views to illustrate the arrangement of the strain film and gel electrodes in the horizontal plane. **b**, Detailed dimensions and structural characteristics of the Coupsensor.


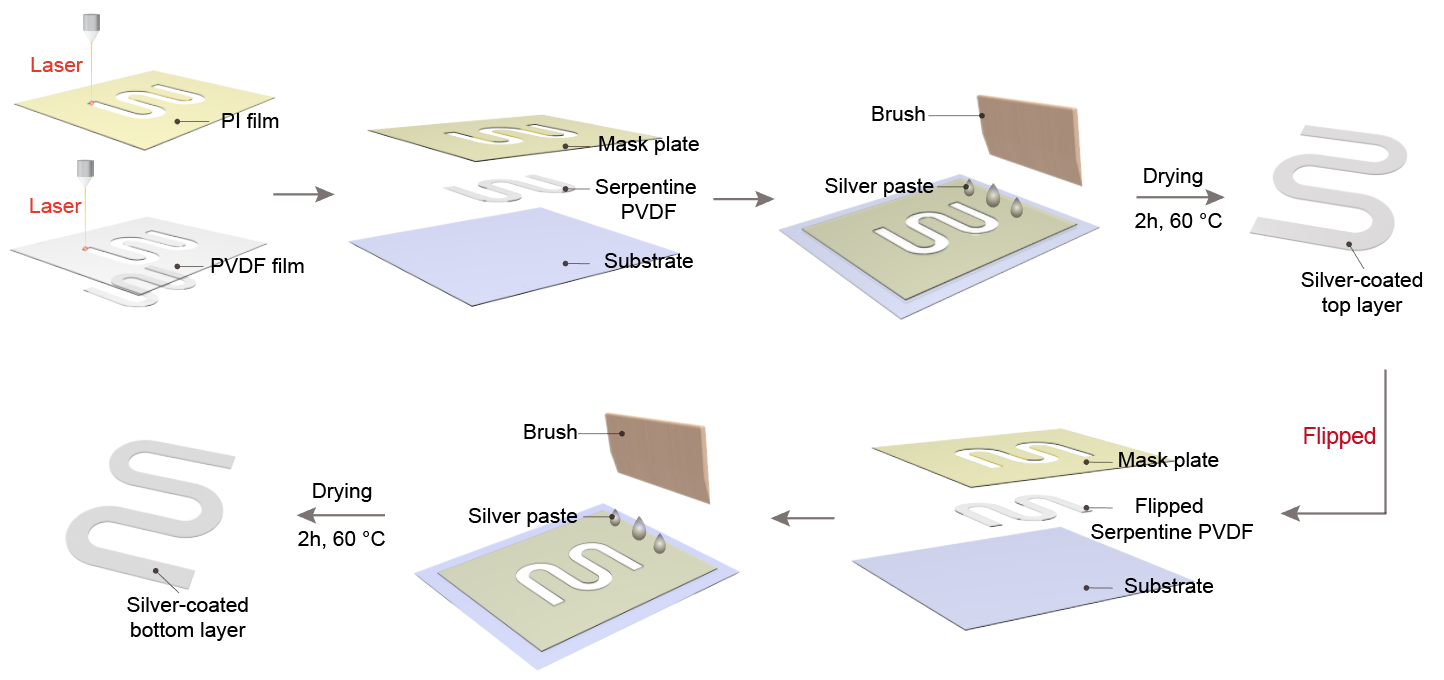


**Supplementary Fig. 2.** Schematic of the fabrication process for the serpentine PVDF electrode. Initially, a laser cutting machine is used to cut a serpentine PVDF film and a mask plate, which is placed beneath the PVDF and securely fixed. Subsequently, we printed the conductive silver paste with a brush via a mask and placed it in a 60 °C dryer for 2 hours, resulting in an even coating of conductive silver paste on one side of the PVDF film. Similarly, the other side of the serpentine PVDF film undergoes the same silver coating process, yielding a double-sided conductive PVDF piezoelectric sensor. Specifically, in order to avoid shorting the top and bottom electrodes, we wipe the edges with alcohol until the edge of top and bottom electrodes do not conduct.


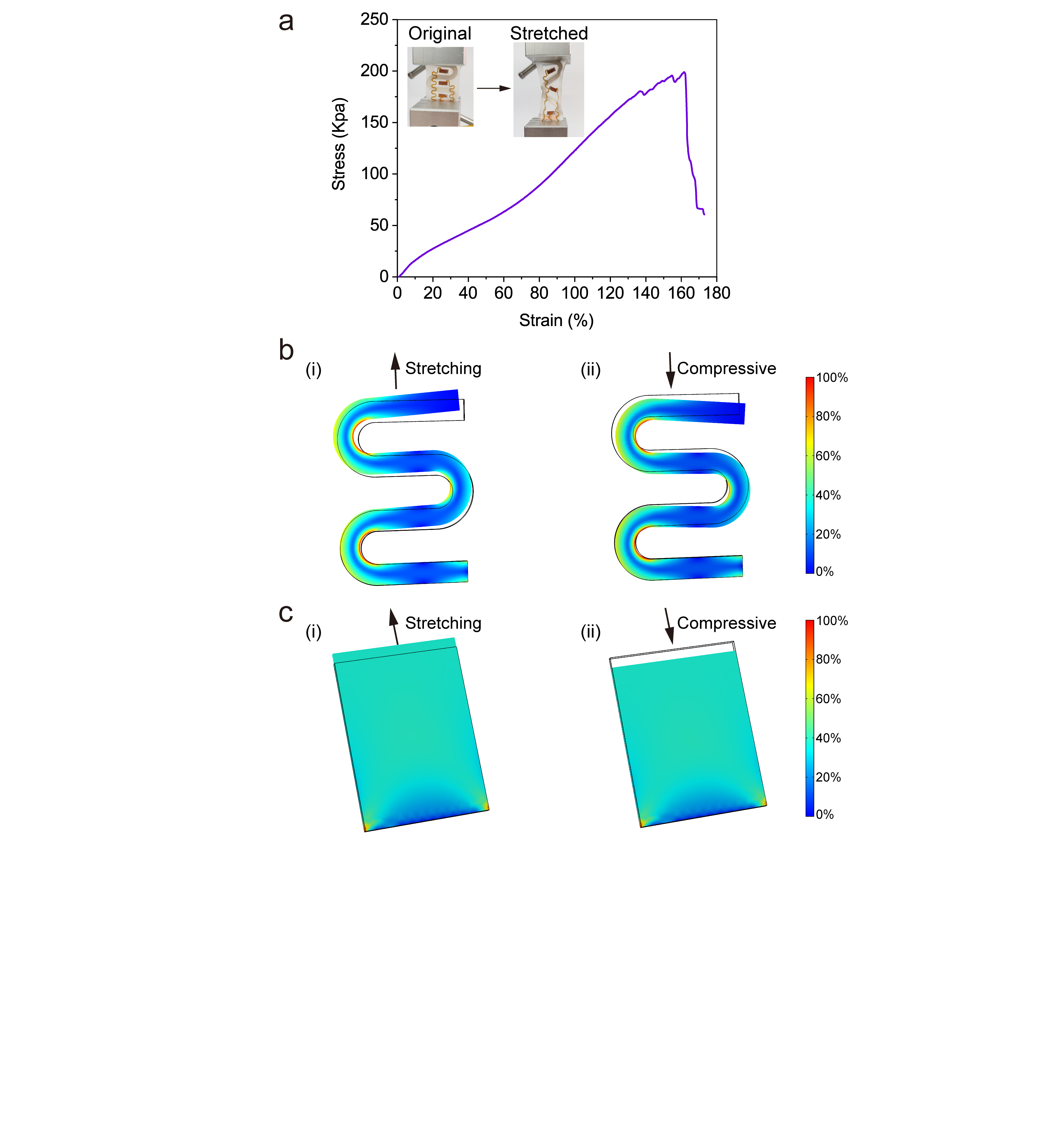


**Supplementary Fig. 3.** Stretchability and simulation analysis of PVDF and Ecoflex. **a**, The stretchability of Coupsensor. **b**, The strain distribution of serpentine PVDF and (**c**) Ecoflex under a specified 4 N stress, including (**i**) stretching and (**ii**) compressive force. The device adhered to the skin is subjected to a stretching force when the patient performs plantarflexion. And then, local stress concentration makes the PVDF equivalent to having a downward pressing force. Conversely, the PVDF shrinks when the patient performs dorsiflexion.


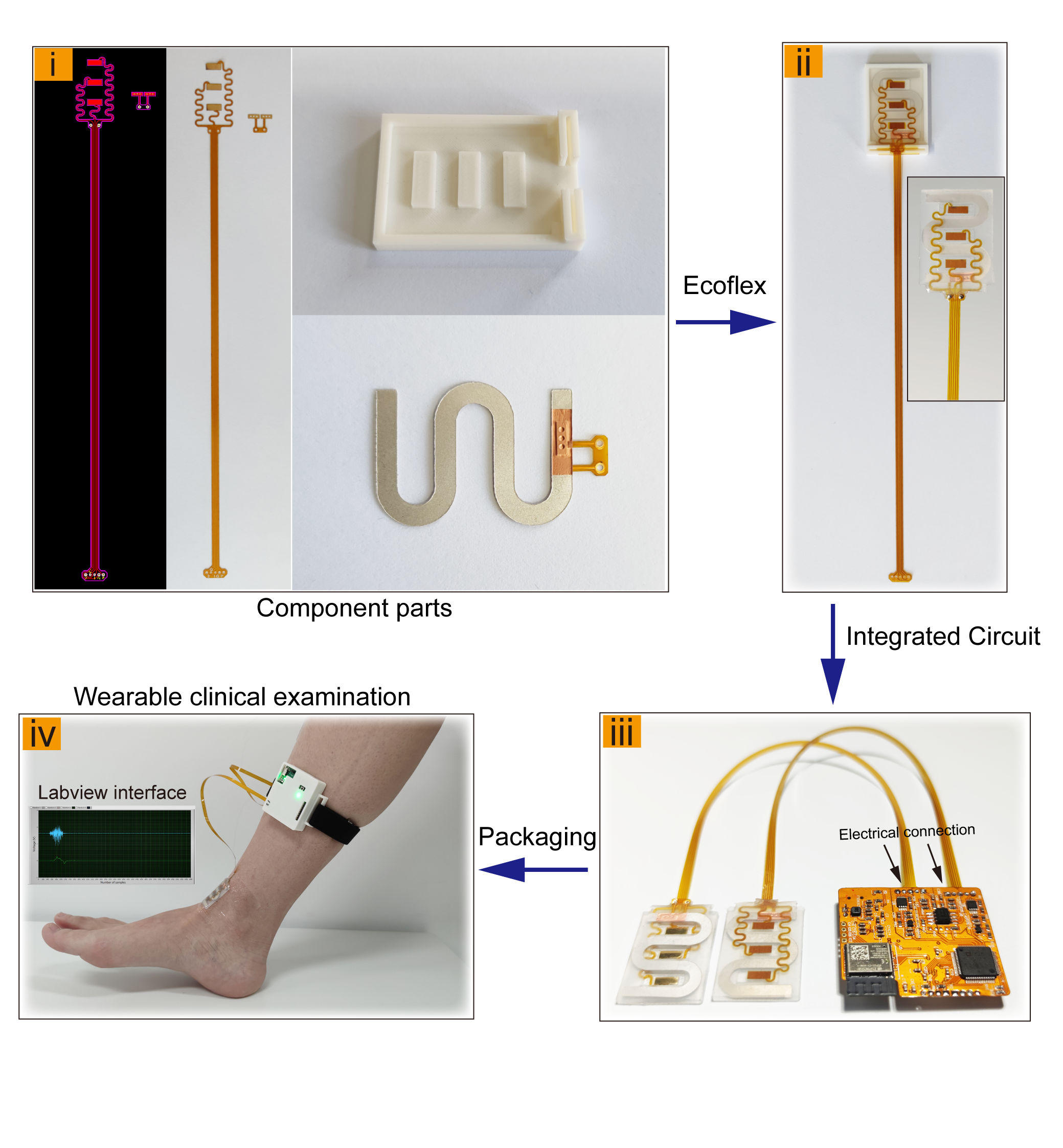


**Supplementary Fig. 4.** Schematic diagram of the fabrication process of Coupsensor. Iintialy, FPCB1, FPCB2, 3D mold and the serpentine piezoelectric film (**i**) were prepared, and the Coupsensor (**ii**) was prepared by mixing parts A and B of Ecoflex™ 30 FAST in an equal ratio of 1:1 and filling the 3D mold in reverse. Subsequently, (**iii**) the Coupsensor is electrically connected through-hole to the acquisition circuit, forming the unpackaged integrated system combing myoelectric and piezoelectric sensors. Finally, (**iv**) the integrated circuit was encapsulated in a housing, while an adjustable strap was installed to enable wearable clinical muscle strength detection. Please refer to the Methods section for a comprehensive description of the detailed preparation process.


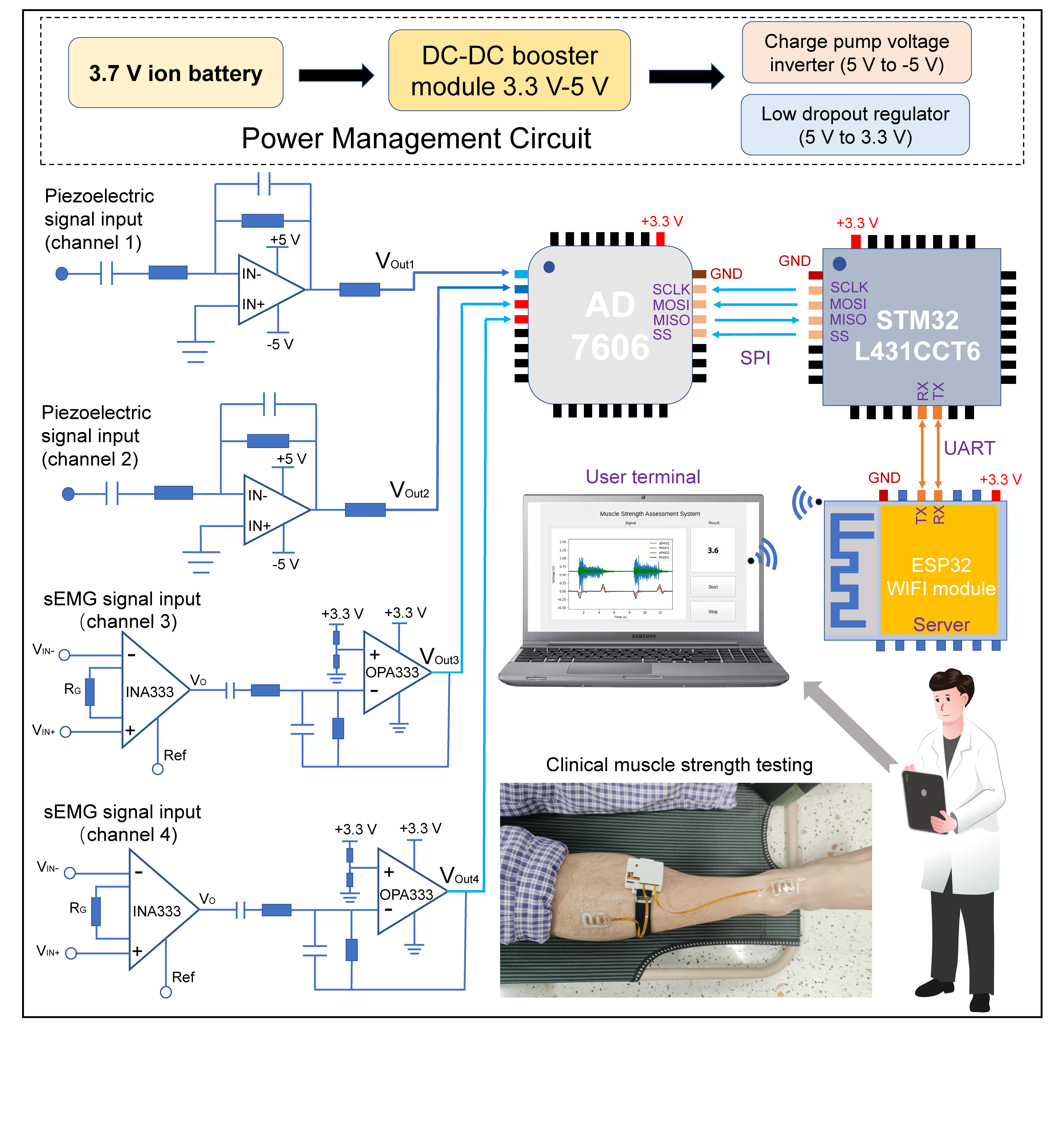


**Supplementary Fig. 5.** Schematic diagram of the signal processing flow of CMSAS. The piezoelectric and myoelectric signals are amplified by their respective amplification circuits and then digitized for real-time wireless transmission via ESP32 module under the control of the master control unit. The received data is further processed and displayed at the terminal via WiFi protocol.


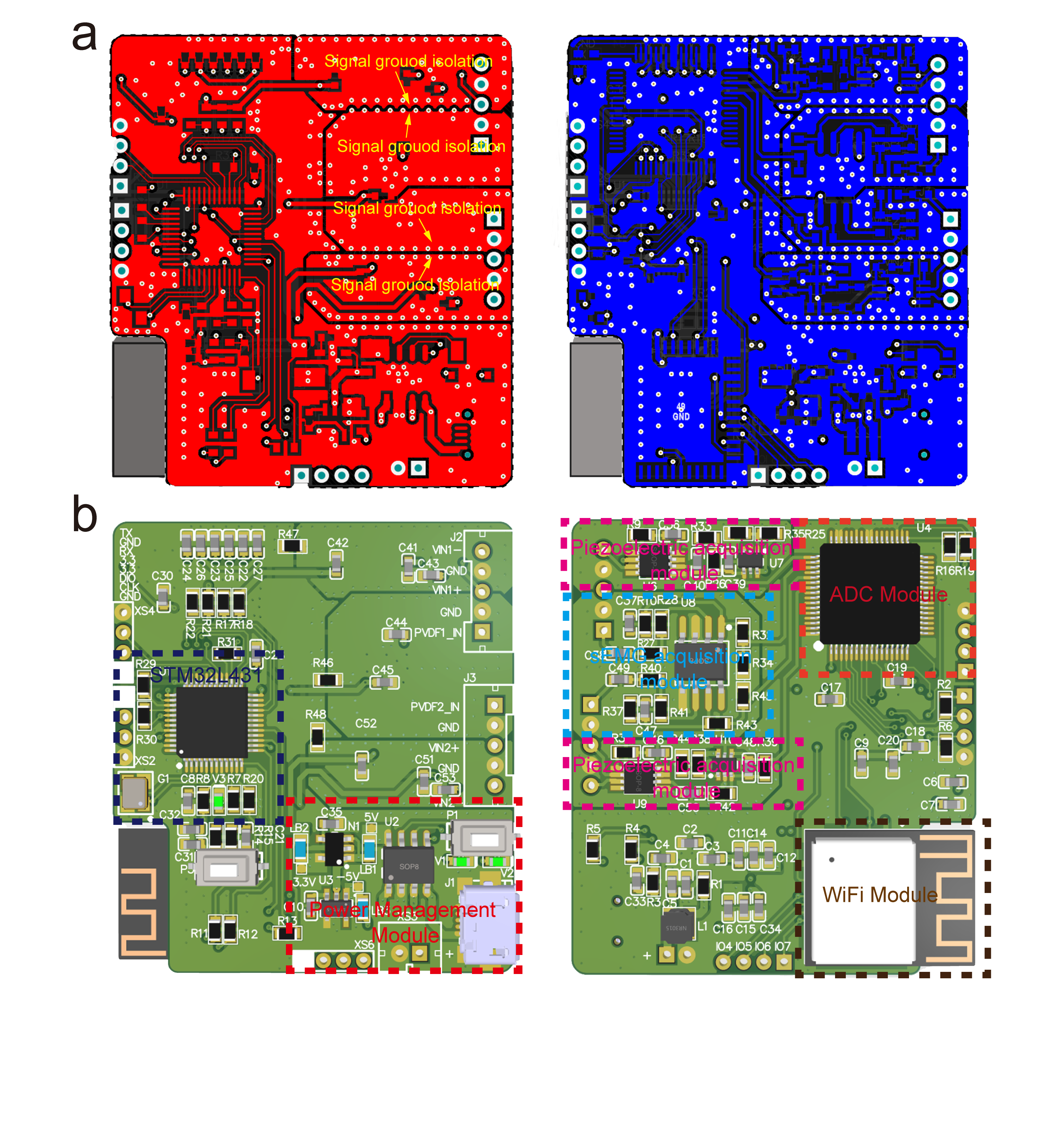


**Supplementary Fig. 6.** Integrated hardware schematic diagram. **a**, 2D Schematic Diagram: The figure presents a comprehensive view of the four-layer integrated circuit, designed using Altium Designer 18 software. It showcases the various components of the circuit, including the multichannel charge amplification module, sEMG acquisition module, PMIC, wireless communication module, AD conversion module, and main control module. Notably, the signal amplification module is carefully designed with ground isolation to mitigate signal interference. **b**, 3D Visualization Diagram: This corresponding diagram provides a three-dimensional visualization of the integrated circuit, offering a more detailed representation of its structure and arrangement of components.


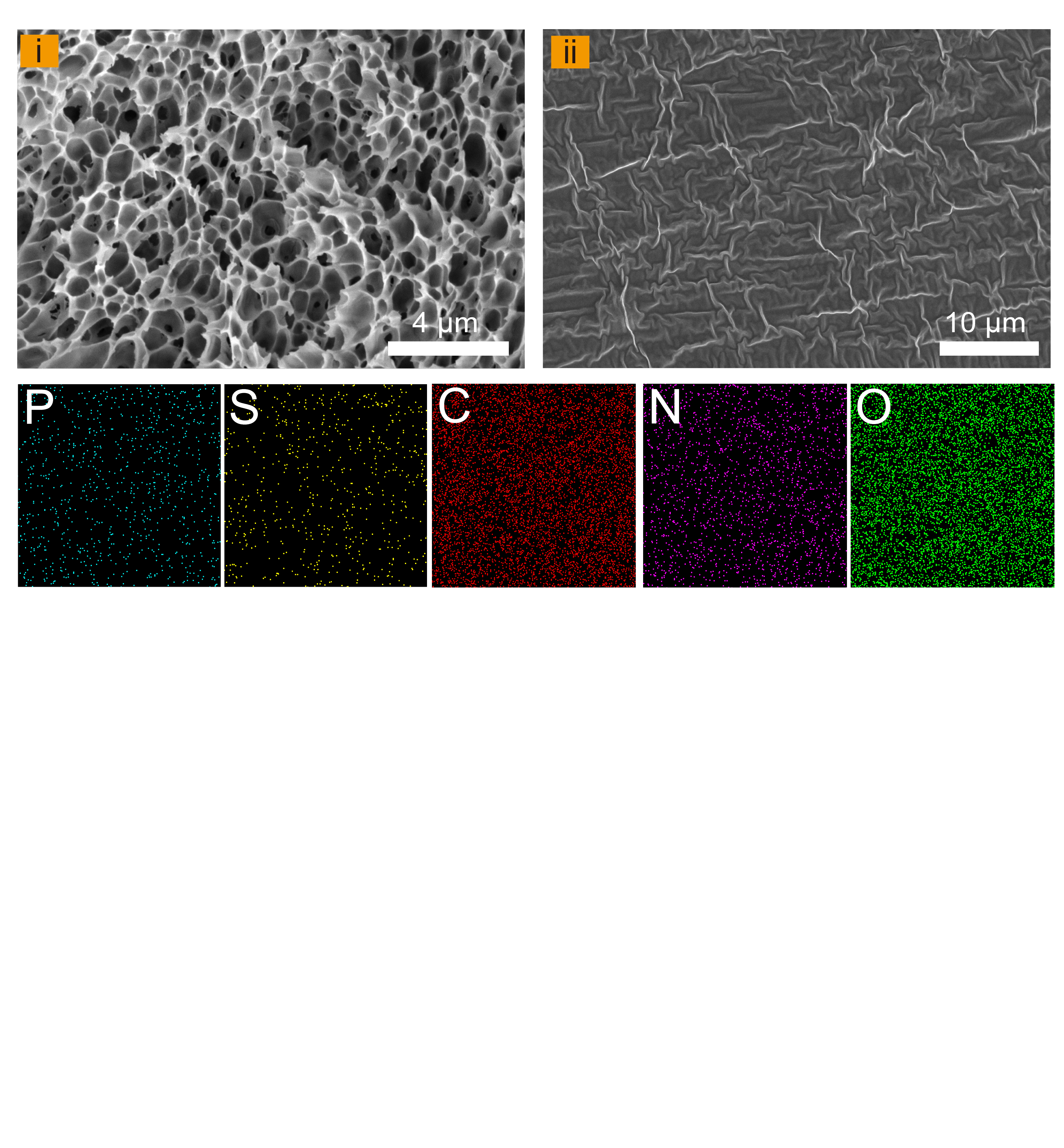
**Supplementary Fig. 7.** Cross-sectional SEM images and elemental analysis of SIH. **a**, SEM images of SIH without the addition of LiCl particles reveal a uniform and dense three-dimensional (3D) network structure that enhances the hydrogel's mechanical properties and provides ample space for ion diffusion in the 3D network channels. **b**, SEM photographs of SIH with the addition of LiCl. Since the addition of a certain concentration of LiCl significantly lowers the freezing point of pure water in the hydrogel electrolyte and inhibits the crystallization behavior of water molecules. Therefore, during the quenching and freeze-drying process using liquid nitrogen, the presence of LiCl prevents water evaporation, rendering the 3D network structure of SIH surface invisible. These experiments demonstrate that SIH exhibits excellent anti-freezing properties^1,2^. **c**, Energy-dispersive X-ray spectroscopy (EDS) analysis reveals the elemental composition of SIH, mainly including P, S, C, N, and O.


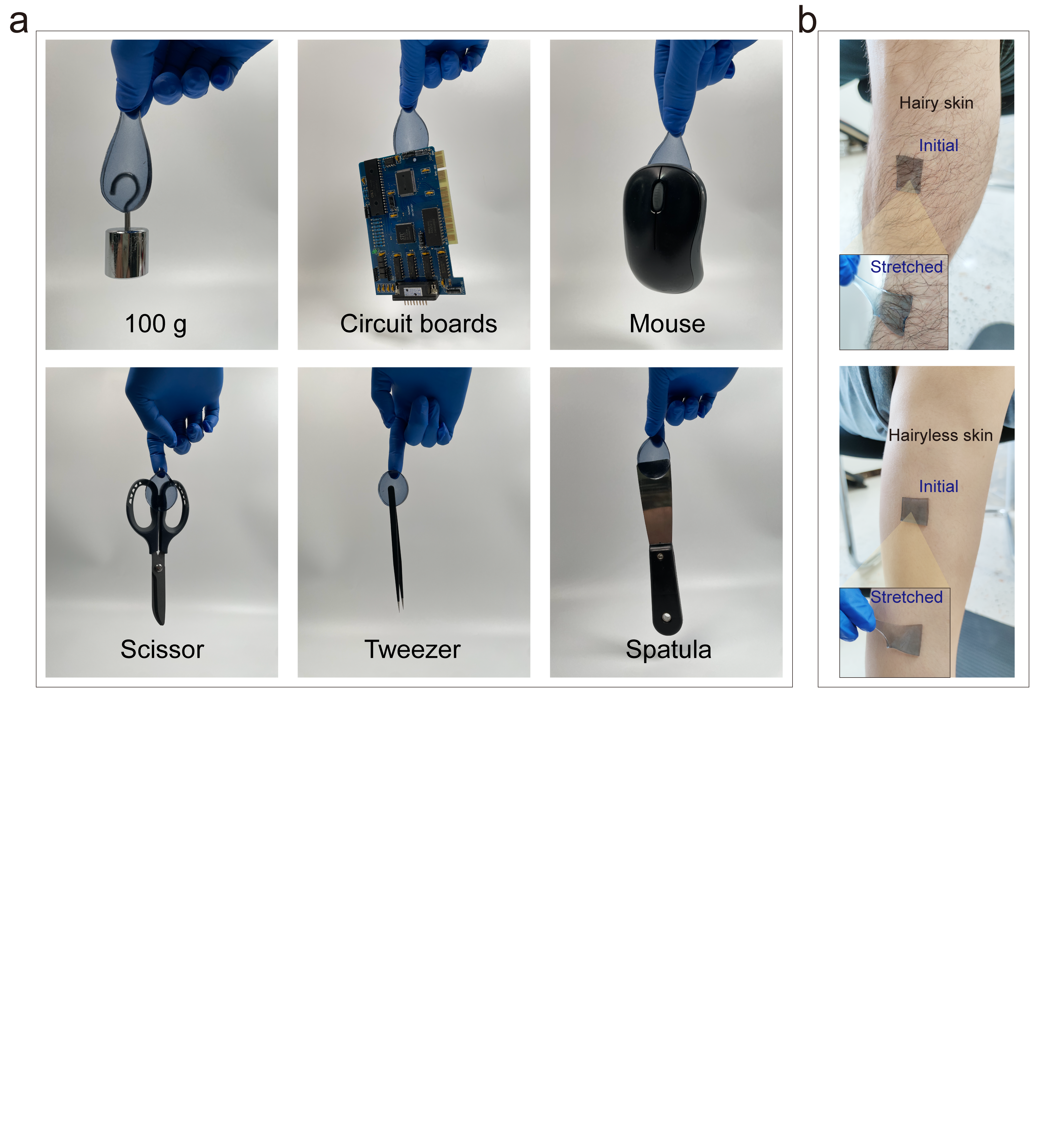


**Supplementary Fig. 8.** Adhesion experiment of SIH. **a**, The SIH demonstrates exceptional adhesive properties, displaying strong adhesion to various objects of different weights and shapes. The objects tested include weights, circuit boards, a computer mouse, scissors, a tweezer, and a spatula. The hydrogel's adhesive capabilities allow for secure attachment to these items, highlighting its potential for practical applications. **b**, The SIH gel exhibits excellent adhesion to the skin surface in hairy or hairless regions, facilitating the reduction of contact impedance between the skin and the electrode.


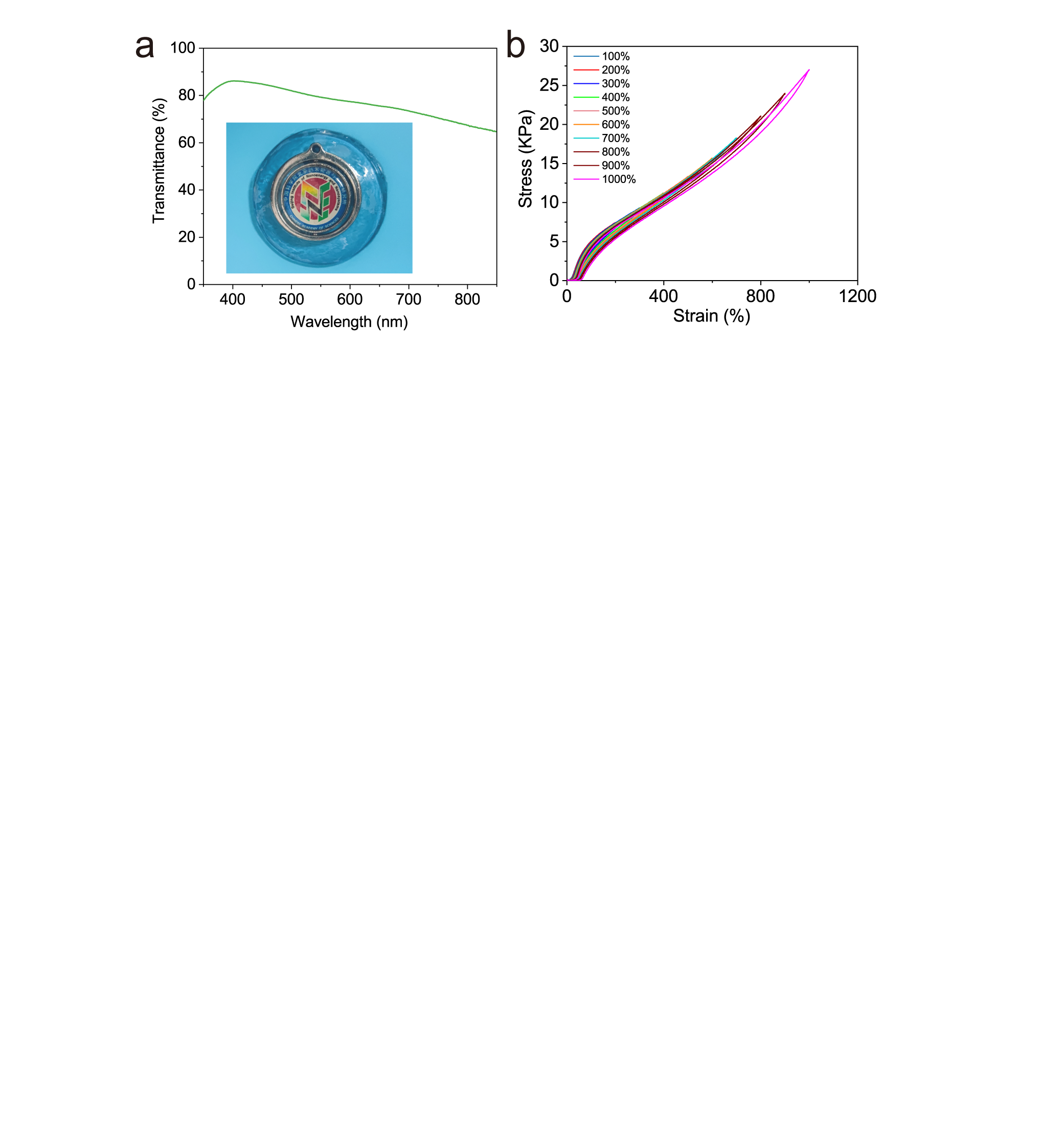


**Supplementary Fig. 9.** Characterization of transparency and tensile cycling of the SIH. **a**, Optical transparency: the SIH demonstrates outstanding light transmission properties within the visible wavelength range (390-760 nm), exhibiting a transmittance exceeding 70%. **b**, Tensile cycling behavior: tensile stress-strain curves of SIH with varying proportions strain ranging from 100% to 1000%.


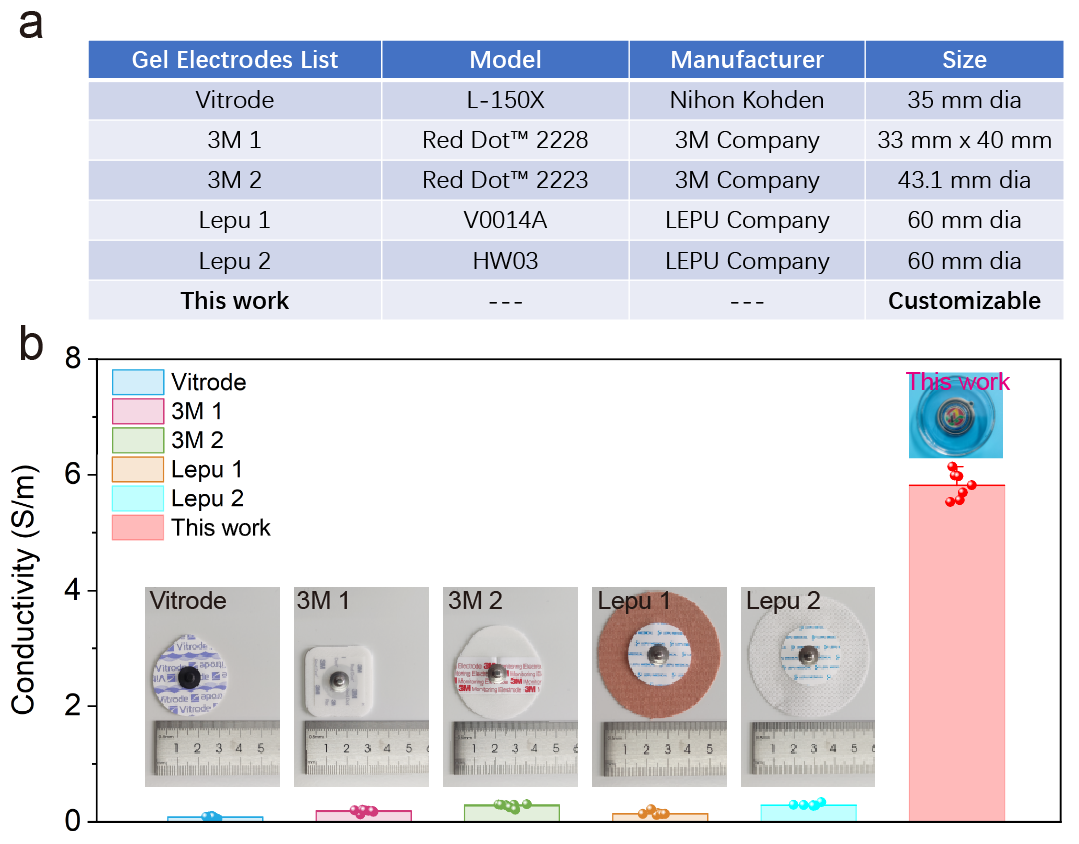


**Supplementary Fig. 10.** Comparison of 5 commercial wet electrodes and SIH gel. **a**, Details of 5 commercial wet electrodes and SIH gel. **b**, Conductivity of SIH gels compared to commercial wet electrodes. The electrical conductivity of five different commercial wet electrode gels and SIH gel was measured by electrochemical workstation under uniform test conditions (test electrode area 60 mm² and thickness 2 mm). The measurement results demonstrated that the electrical conductivity of our hydrogel electrodes (~6 S m^-1^) was significantly higher than that of the commercial electrodes.


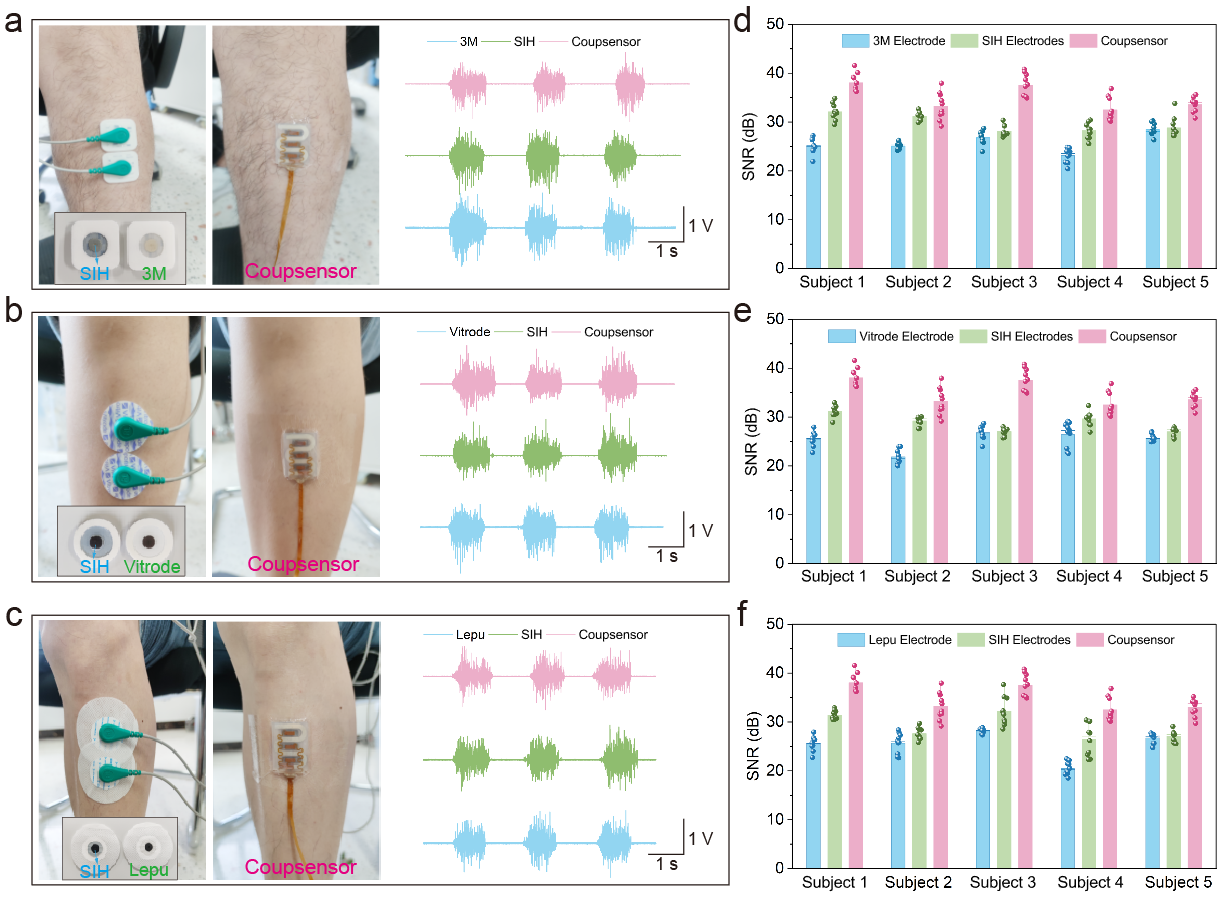


**Supplementary Fig. 11.** EMG signals and their corresponding SNR for five healthy subjects using three types of commercial electrodes, SIH electrodes, and the Coupsensor. In the experiment, tests with each set of electrodes were conducted in the same environment using identical EMG acquisition hardware. Prior to testing, the muscle belly of the TA muscle of each subject was first marked to control the equal spacing of the two electrodes at each attachment, the reference electrode was uniformly affixed to the knee joint, and the Coupsensor was attached with the center of the two marked points. Additionally, the subjects' skin was cleaned with alcohol before each test to ensure uniform skin conditions between tests. Furthermore, commercial electrode gels were removed and replaced with SIH electrodes of the same size and thickness, repeating the EMG signal acquisition for comparative testing with control variables. Subjects were required to have non-fatigued muscles during testing and were instructed to perform ten dorsiflexion movements with maximum effort while ensuring the stability of the EMG signals in each acquisition. **a-c**, Photos of testing for the TA muscle of three subjects using the 3M rectangular electrode, the Vitrode electrode, and the Lepu electrode, respectively, along with their corresponding EMG raw data segments are presented. **d-f**, The SNR comparisons for five healthy subjects using the 3M, Vitrode, and Lepu electrodes, respectively. The result indicates that under identical testing conditions, SIH hydrogel electrodes, in comparison to commercial electrodes, consistently exhibit comparable or superior EMG signal quality and SNR. Furthermore, the Coupsensor outperforms both commercial wet electrodes and SIH-replaced commercial electrodes in terms of signal quality and SNR due to the possible effects of electrode, structural, dimensional, and size.


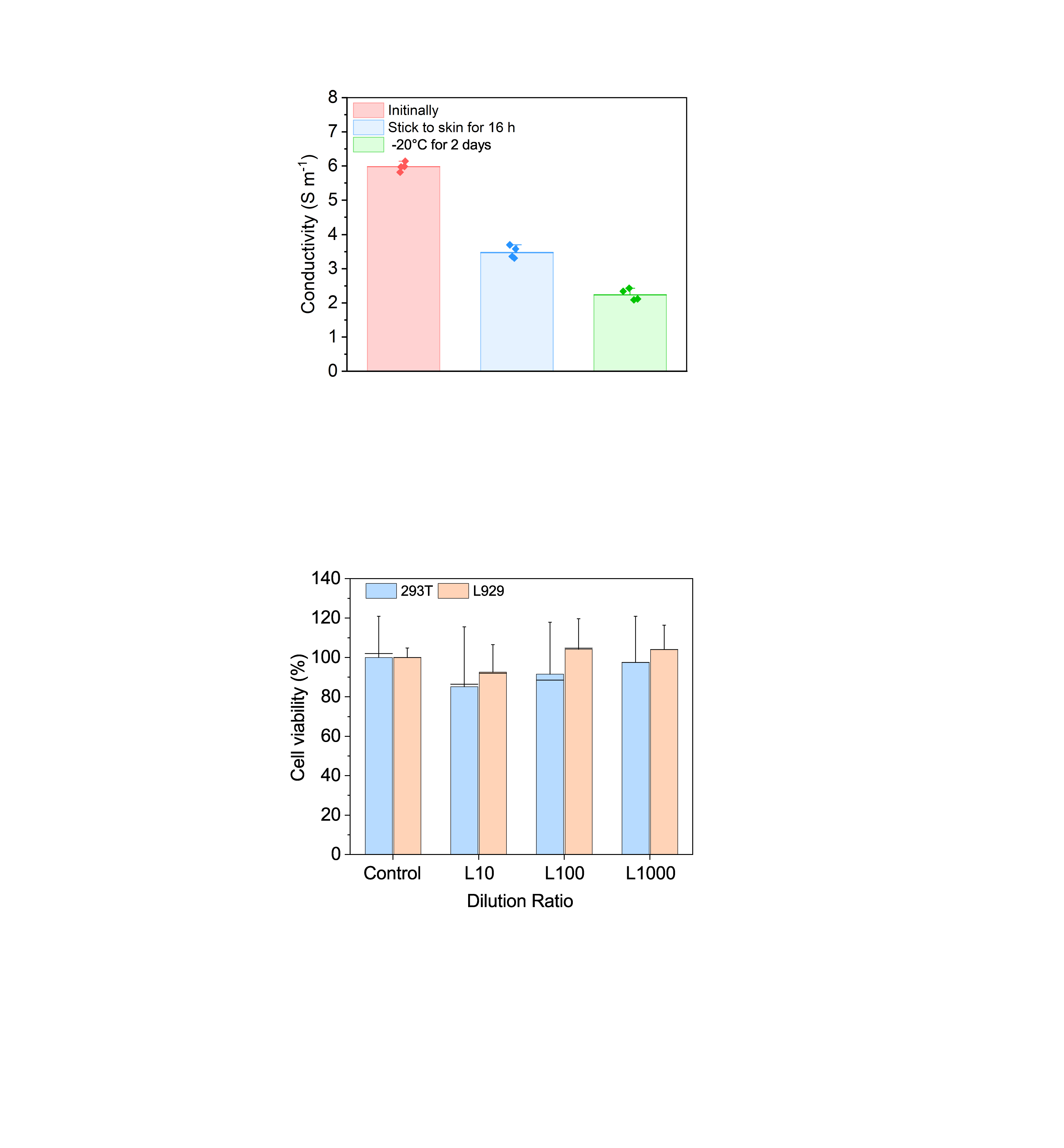


**Supplementary Fig. 12.** Conductivity measurements of the SIH under different conditions. (a) Initial conductivity of the hydrogel (conductivity: ~ 6 S m^-1^), and the conductivity after being applied to the skin for 16 hours and exposure to a freezing temperature of -20 ℃ (conductivity more than 2 S m^-1^). The results demonstrate the SIH's stable and reliable conductivity performance under challenging conditions, making it suitable for various applications in the field of wearable electronics and biosensors^3,4^.


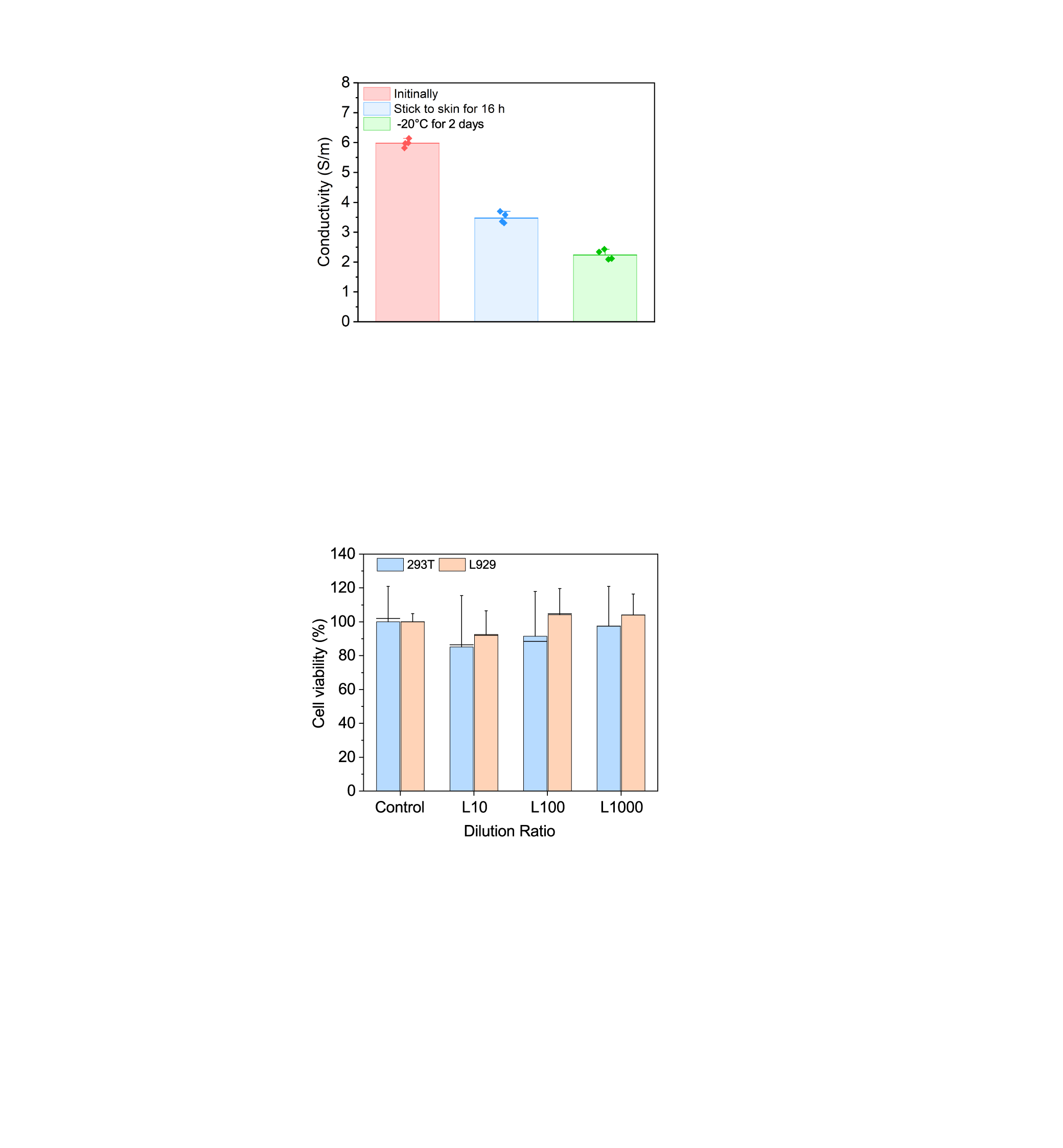


**Supplementary Fig. 13.** Cytotoxicity tests assay after 48-hour cell culture. Cytotoxicity curves of L929 and 293T cells cultured for 48 hours in diluted 10x, 100x, and 1000x stock solutions of SIH. The survival rates of both cell types were above 85%, confirming the cytocompatibility and non-toxicity of SIH^5,6^.


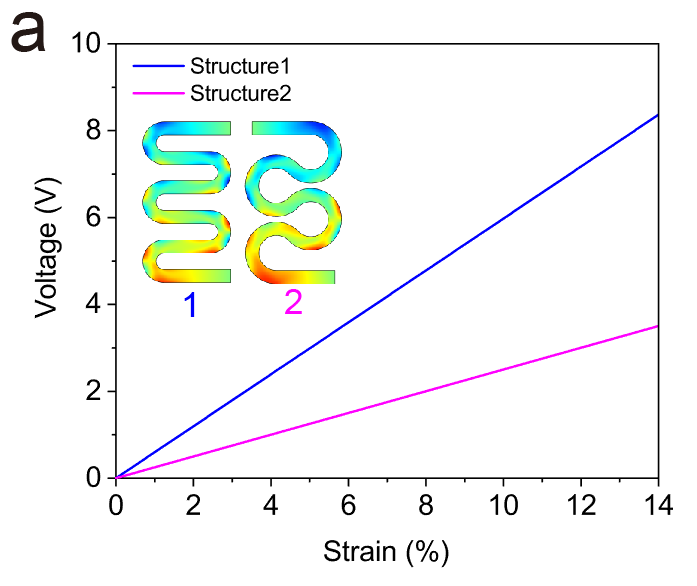


**Supplementary Fig. 14.** Simulated potential analysis of piezoelectric films with different serpentine structures 1 and structures 2.


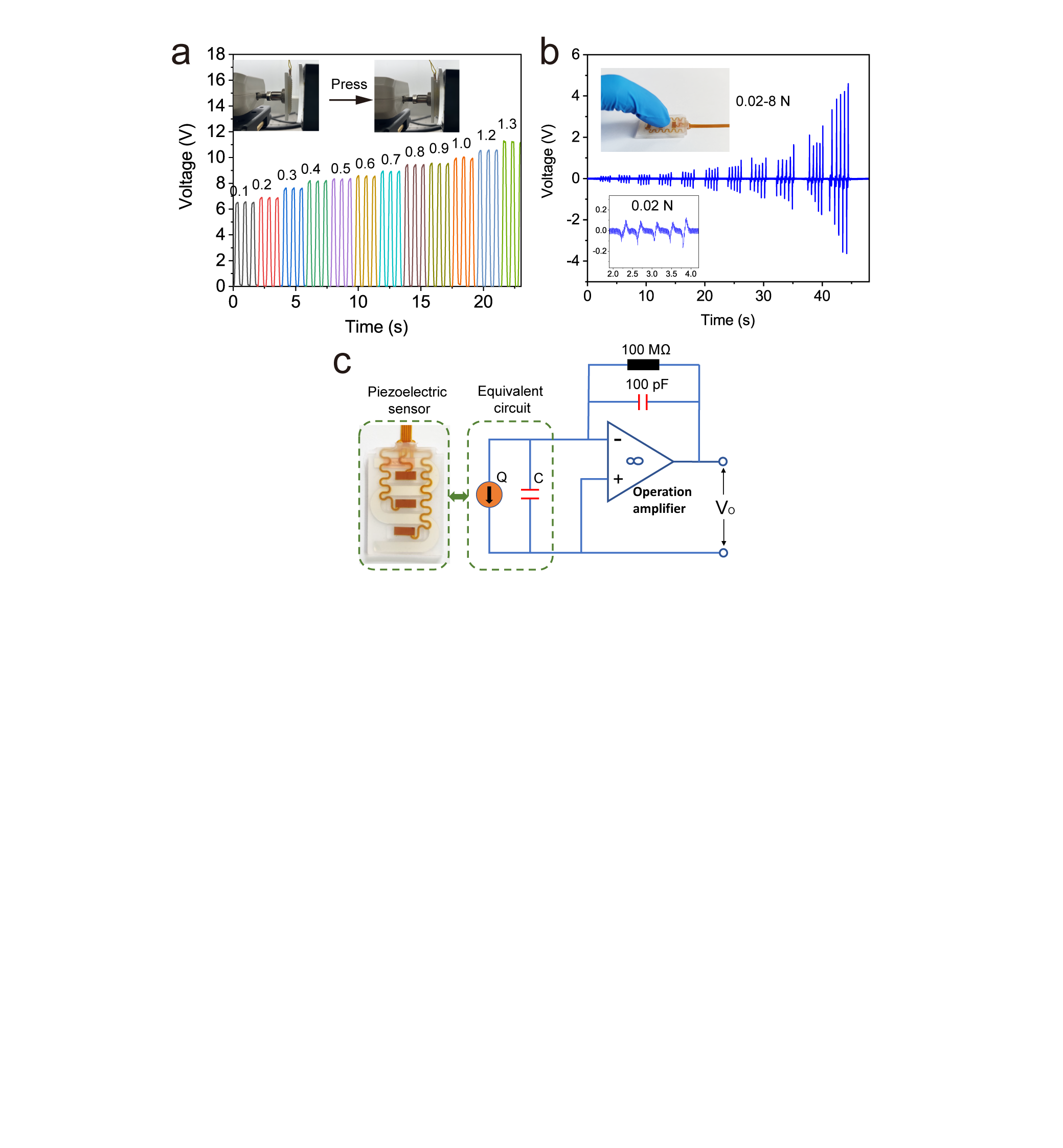


**Supplementary Fig. 15.** Pressure characterization of the Coupsensor and its equivalent circuit. **a**, Output curves of Coupsensor under various pressures (0.1-1.3 N), the inset shows the experimental setup with a Keysight 6514 electrostatic meter applied for data acquisition. **b**, Output curves of the Coupsensor under different applied press forces (0.02-8 N), and (**c**) accompanied by the corresponding charge acquisition equivalent circuit^7^.


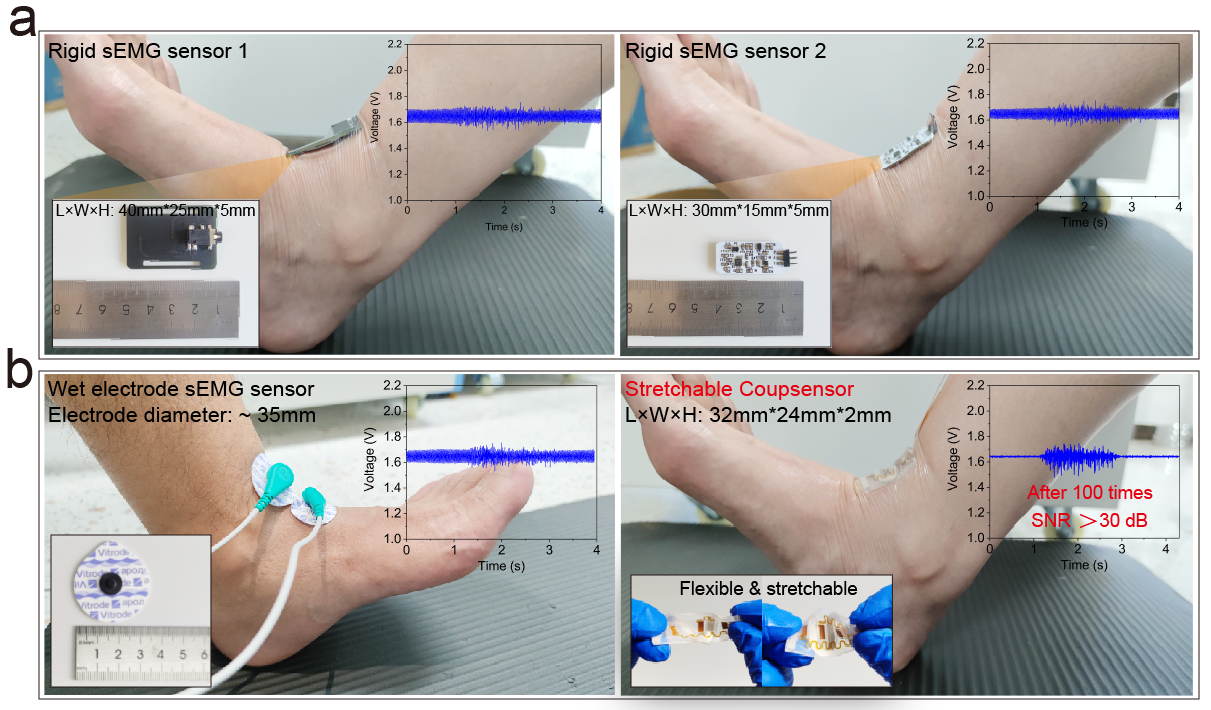


**Supplementary Fig. 16.** Detection ability of the flexible Coupsensor compared to commercially available wet and dry electrode EMG sensors on EHL muscles. **a**, Rigid dry commercial sensors 1 and 2 from DFRobot (Sensor 1 each electrode size: 20 mm × 10 mm; Sensor 2 each electrode size of 10 mm×3.5 mm) for testing the EHL muscle. During forceful dorsiflexion, rigid EMG sensors failed to effectively collect muscle electrical signals due to poor electrode-skin contact, and all three wearers reported discomfort or pain. The upper right inset shows the first acquisition of EMG signals by the two rigid sensors when the dorsum of the foot was not fully flexed. **b**, Testing with commercial wet EMG electrodes compare to the Coupsensor. The left image represents testing the EHL muscle using the Vitrode electrode (diameter ~3.5 cm). During forceful dorsiflexion, after repeating the motion five times, the Vitrode electrode significantly detached, reducing its contact with the skin and rendering it incapable of continuous and effective EMG signal acquisition. The upper right inset data displays the EMG signal acquired during the first completion of the movement. The right image shows EHL muscle testing using the Coupsensor. The flexible and stretchable of the Coupsensor and SIH electrodes allows for excellent skin contact. Even after 100 repetitions action, it continues to maintain high-quality EMG signal acquisition (SNR greater than 30 dB), as shown in the upper right inset data.


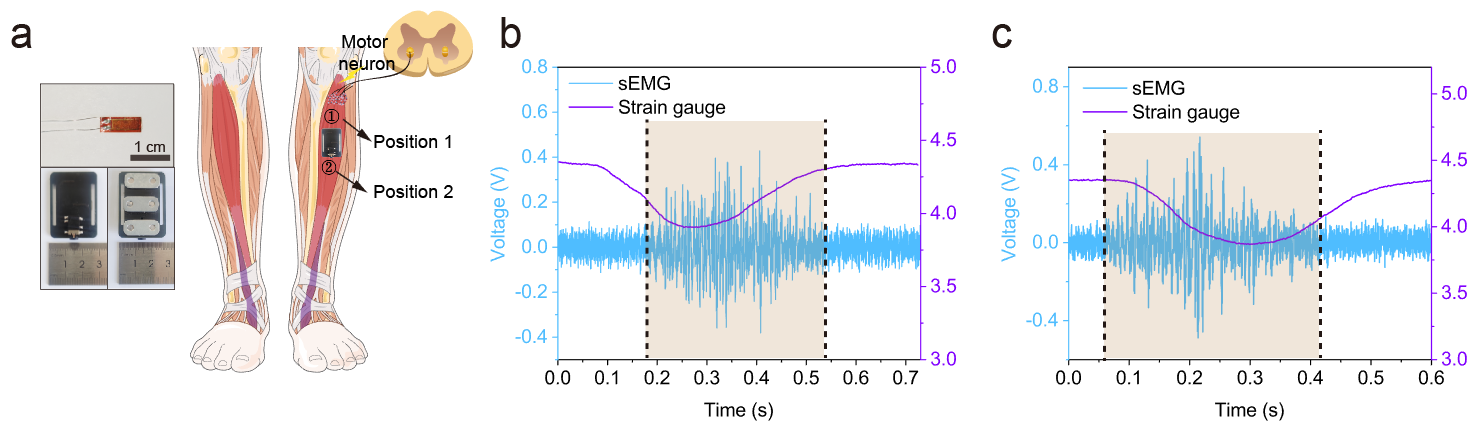


**Supplementary Fig. 17.** Effect of spatial location of myoelectric and strain sensors on multimodal signals. **a**, Investigating spatiotemporal differences in EMG signal and strain signal during self-contraction of the TA muscle using dry-electrode commercial myoelectric sensor and strain sensor. Strain sensors were placed at positions 1 and 2, yielding the EMG and strain signals shown in (**b**) and (**c**) respectively. The results show that during muscle excitation–contraction, the separate locations of electromyography and strain sensors may induce phase differences between EMG and strain signals, influencing the assessment of muscle activity.


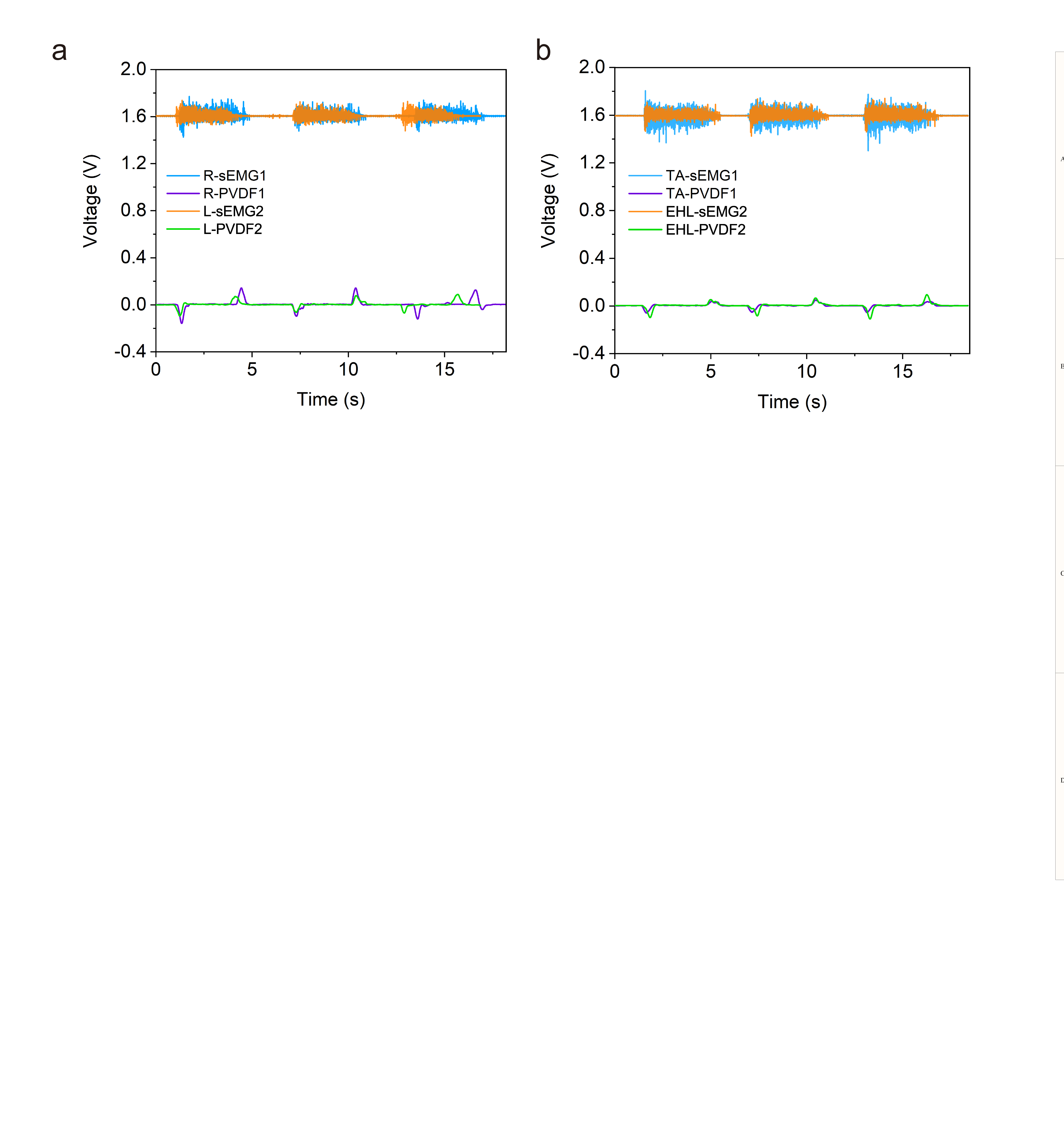


**Supplementary Fig. 18.** Unnormalized data for the corresponding two actions. **a**, Unnormalized data in Fig. 3c and (**b**) Fig. 3d.


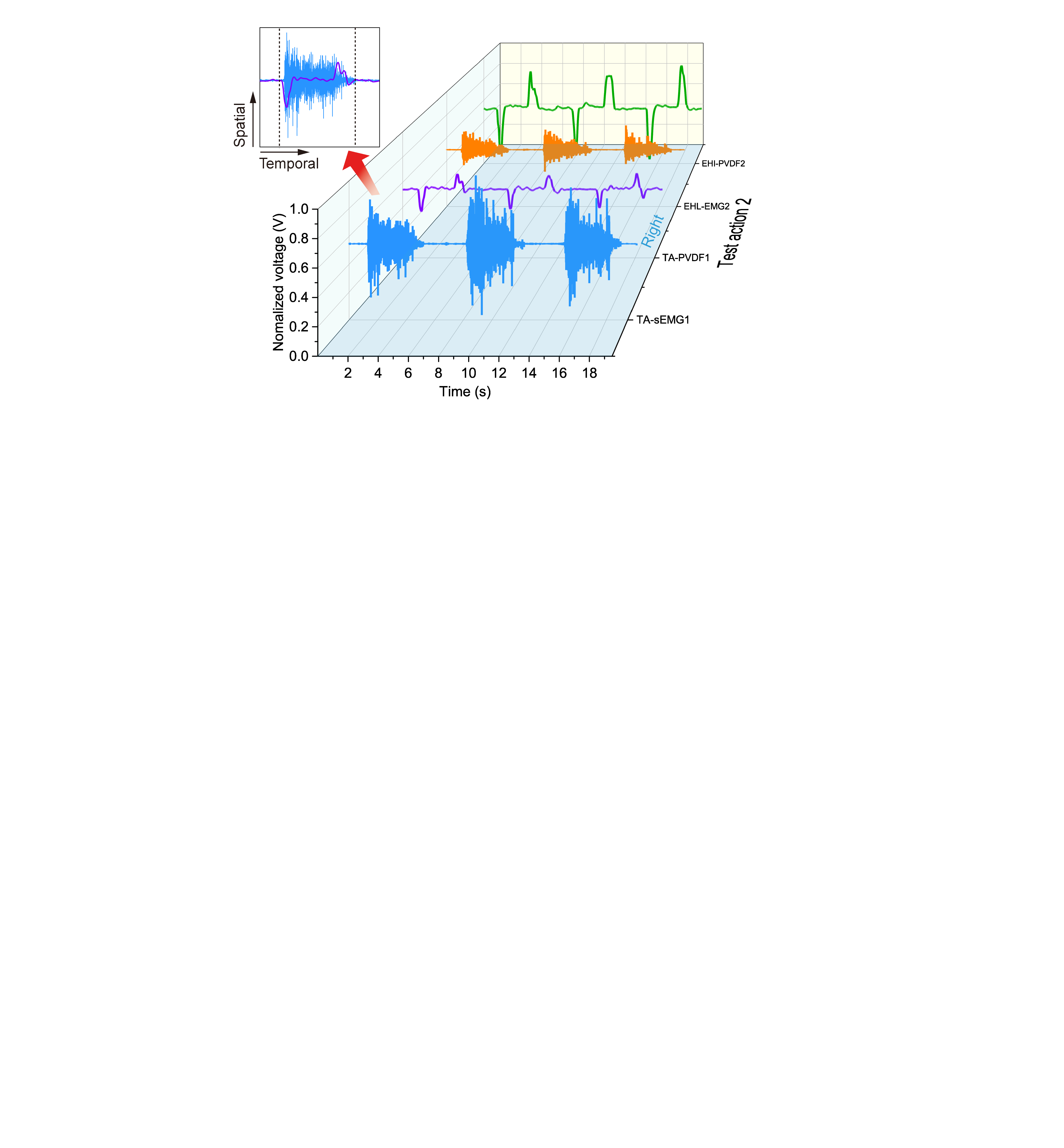


**Supplementary Fig. 19.** Synchronized sEMG and piezoelectric signals of action 2 in one subject's right leg. The enlarged image in the top left corner showcases the normalized sEMG and piezoelectric signals recorded from the right EHL muscle during a single occurrence of action 2. The enlarged data indicating the synchronization without phase difference between sEMG and strain signal.


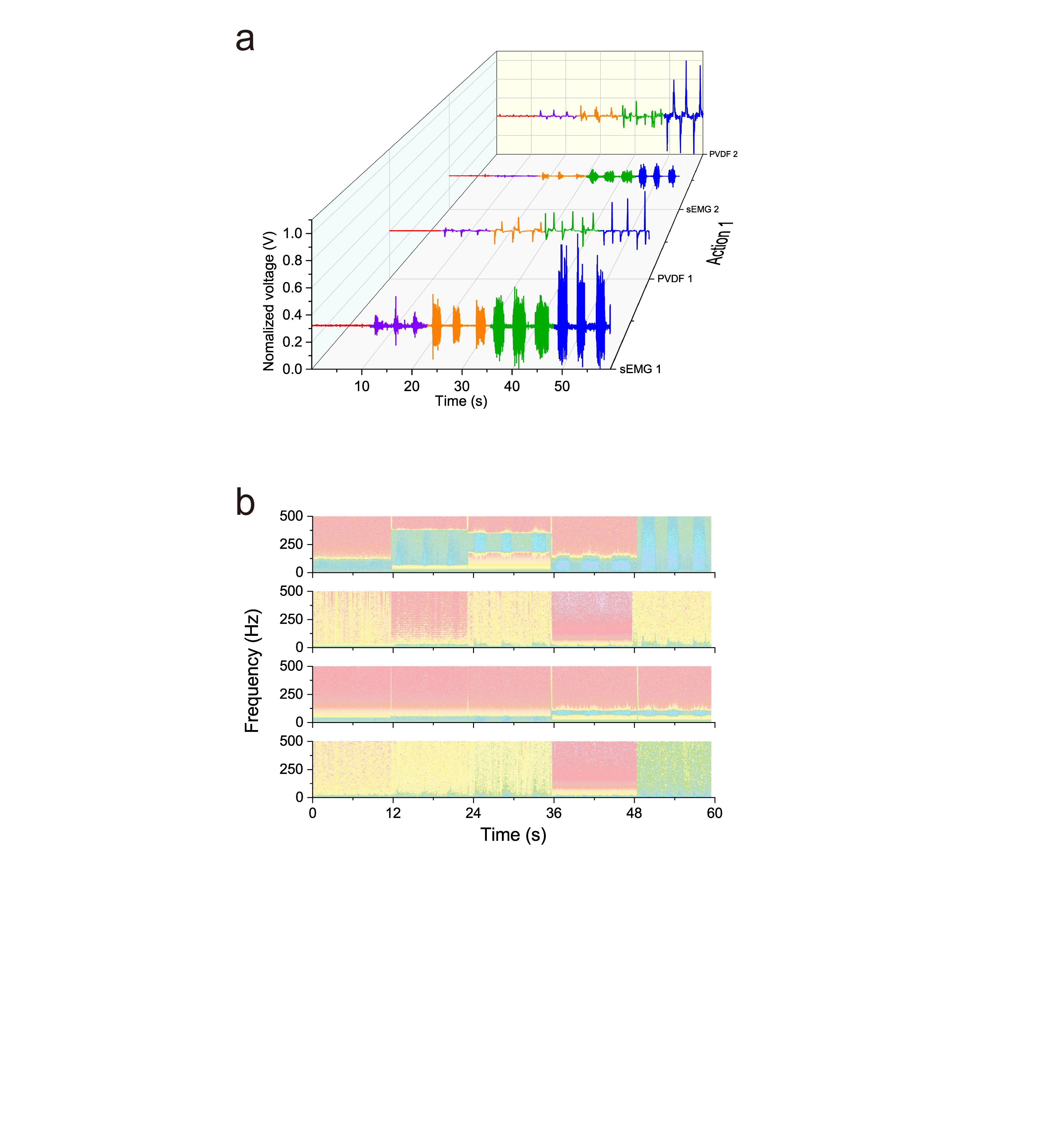


**Supplementary Fig. 20.** Test results of the EHL muscle during action 1 based on 5 patients. **a**, Time domain plots: the curve presents the time domain plots of the EHL muscle for subjects exhibiting muscle strength levels ranging from 1 to 5. Each plot provides a visual representation of the muscle activity during action 1, showcasing variations in signal characteristics across different strength levels. **b**, Frequency domain plots: corresponding frequency domain plots are displayed, providing insights into the spectral composition of the muscle signals.


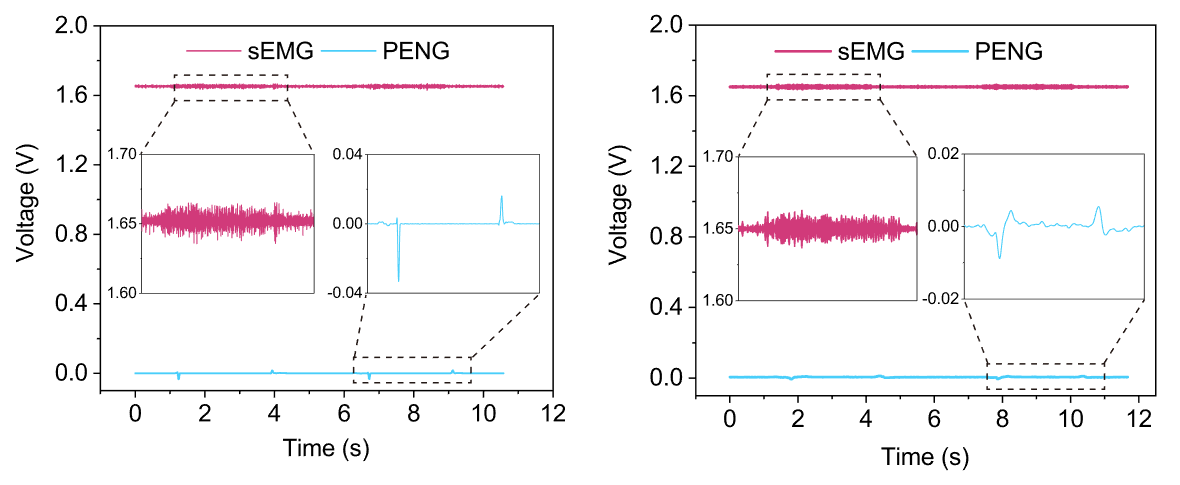


**Supplementary Fig. 21.** sEMG and muscle strain original data of patients with muscle strength function of grade 1. **a**, A patient 27 with muscle strength function grade 1 in the EHL muscle who is unable to produce any joint movement but still has muscle activity. **b**, A patient 29 with a muscle strength function grade 1 in the TA muscle who is unable to produce any joint movement but still has muscle activity.


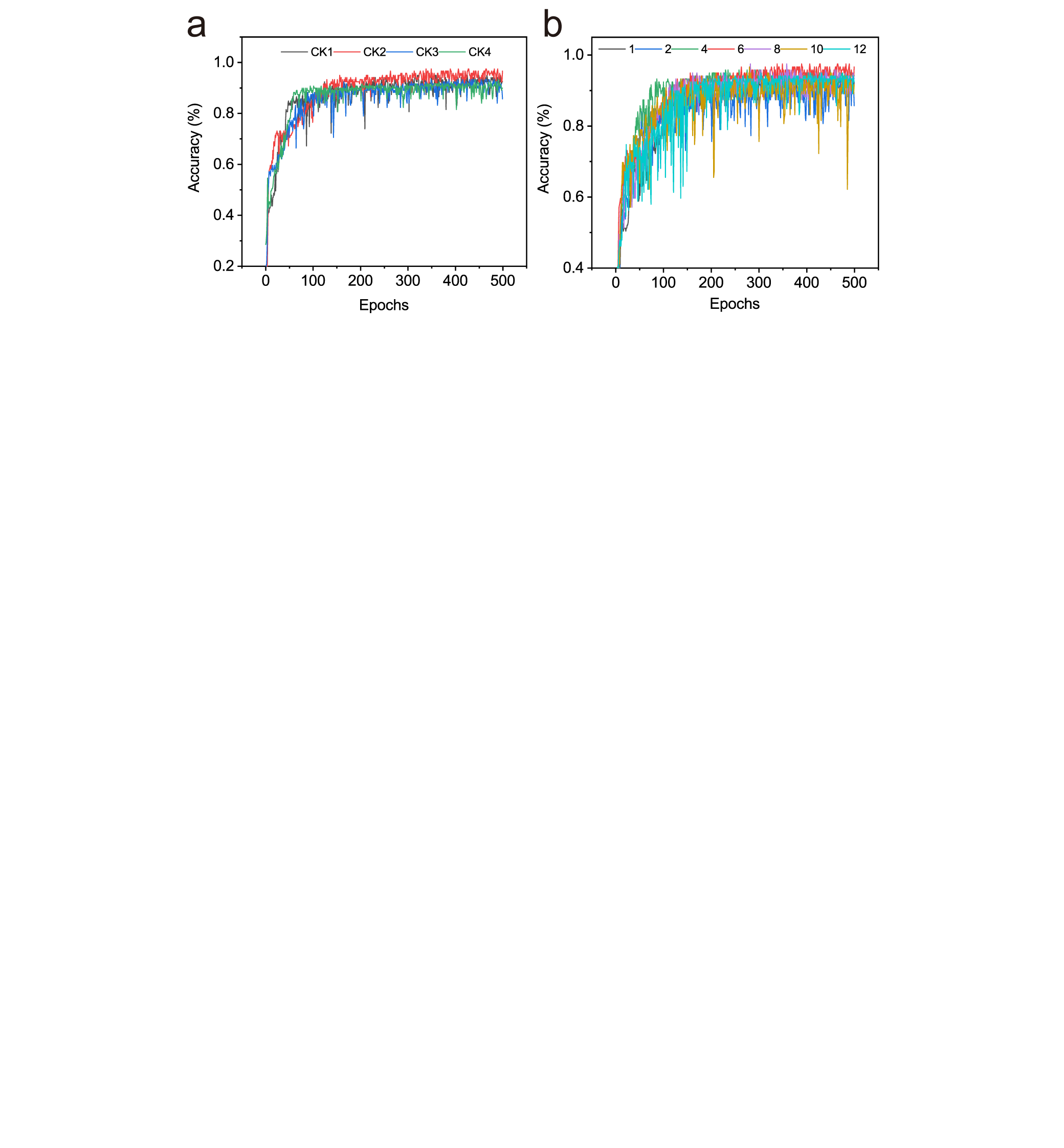


**Supplementary Fig. 22.** Hyper-parameter analysis of TCN models. **a**, The accuracy curve based on dilated CK architectures of TCN model with different dilation rate combinations. **b**, The accuracy curve under different numbers of attention heads in the Transformer model.


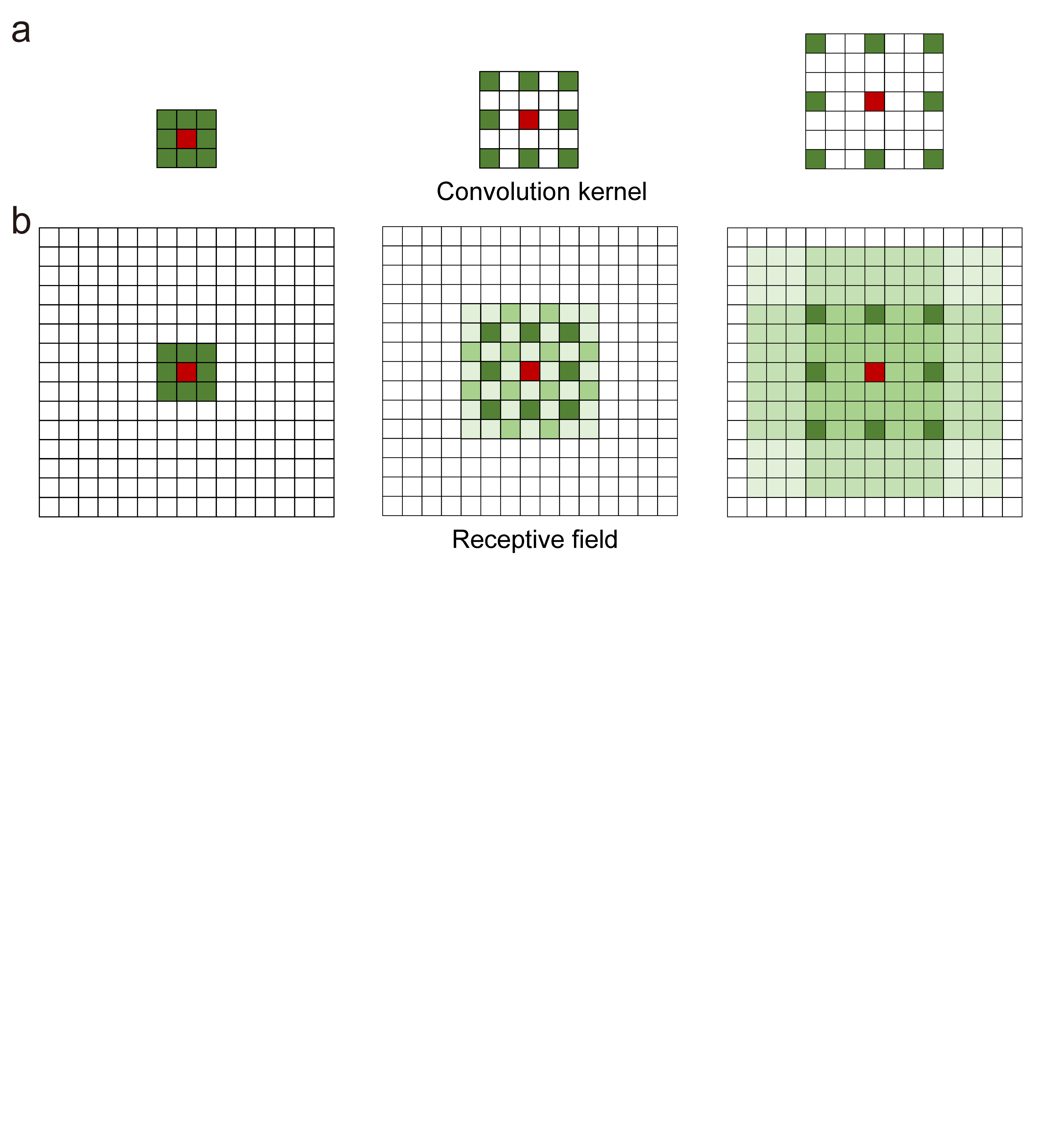


**Supplementary Fig. 23.** The architectures of CK2 in the Tcnformer model. **a**, Architecture of CK2 and (**b**) the corresponding receptive field^8^.


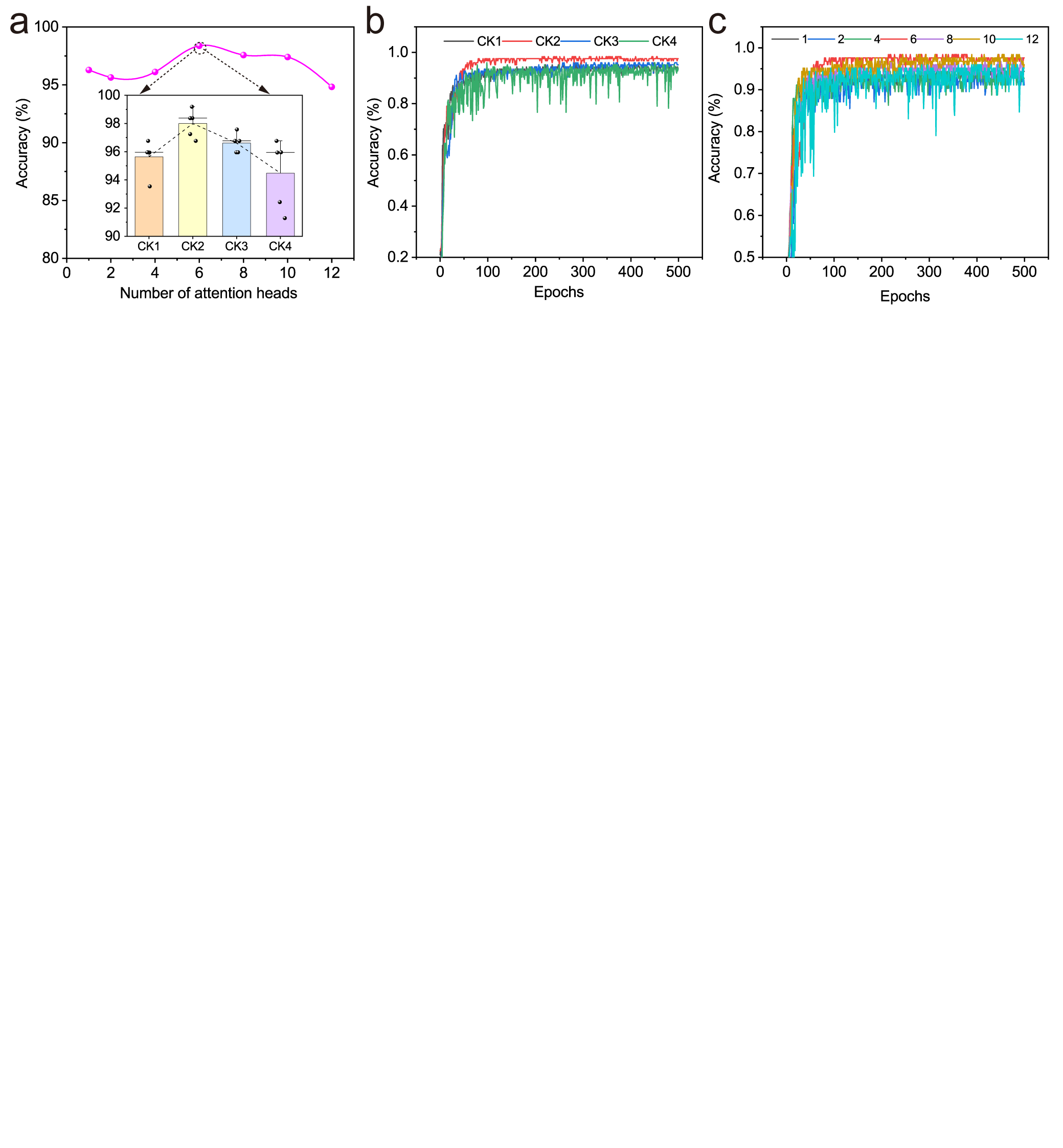
 **Supplementary Fig. 24.** Tcnformer-based hyper-parameters conditioning using EHL muscle data. **a**, the impact of dilated CK architectures of TCN model with different dilation rate combinations and different numbers of attention heads in the Transformer model on the fitting ability of the end-to-end Tcnformer model. **b**, The accuracy curve based on dilated CK architectures of TCN model with different dilation rate combinations. **c**, The accuracy curve under different numbers of heads in the Transformer model. The number of epochs for all models is 500.


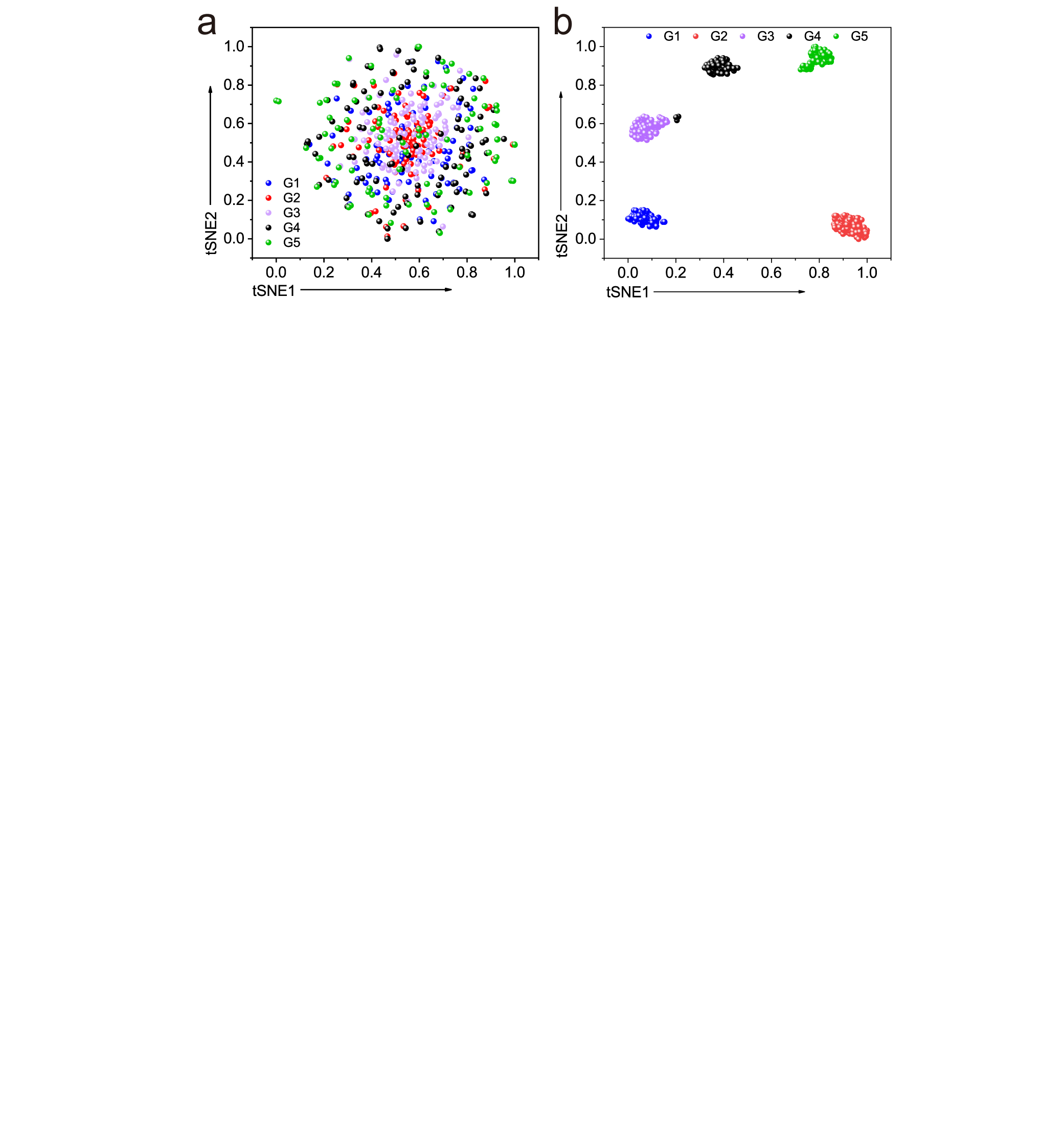


**Supplementary Fig. 25.** t-SNE-based visualization of sEMG signals plots using EHL muscle data. **a**, Original input signal of EHL muscle data and (**b**) visualization map of t-SNE after processing by Tcnformer module.


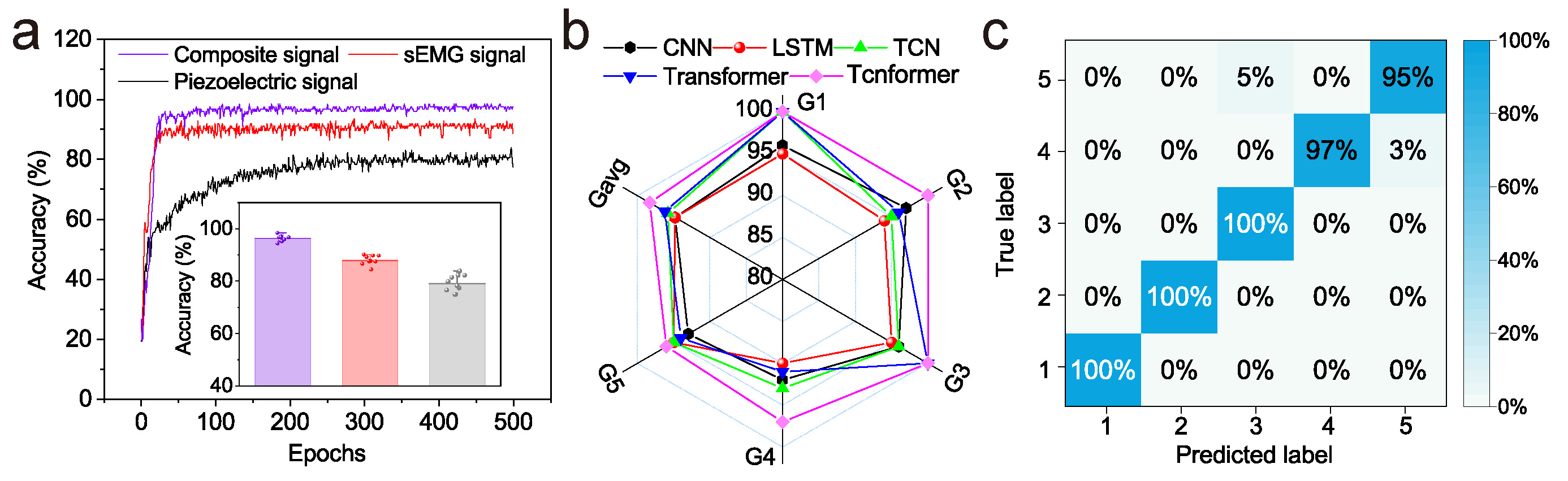


**Supplementary Fig. 26.** Training and grading of EHL muscle data for MS assessment. **a**, Effect of different types of signals (sEMG, piezoelectric and composite signals) from EHL muscles on model accuracy, the inset represent the average of 10 times is ~96.2%. **b**, Accuracy of models based on EHL muscle data under different DL models (including CNN, LSTM, TCN and transformer) compared to Tcnformer models. Tcnformer outperformed all other models, achieving an average accuracy of ~96.2%. **c**, Detailed 5-level confusion matrix probability for EHL MS grade assessment with a highest accuracy of 98.4%.


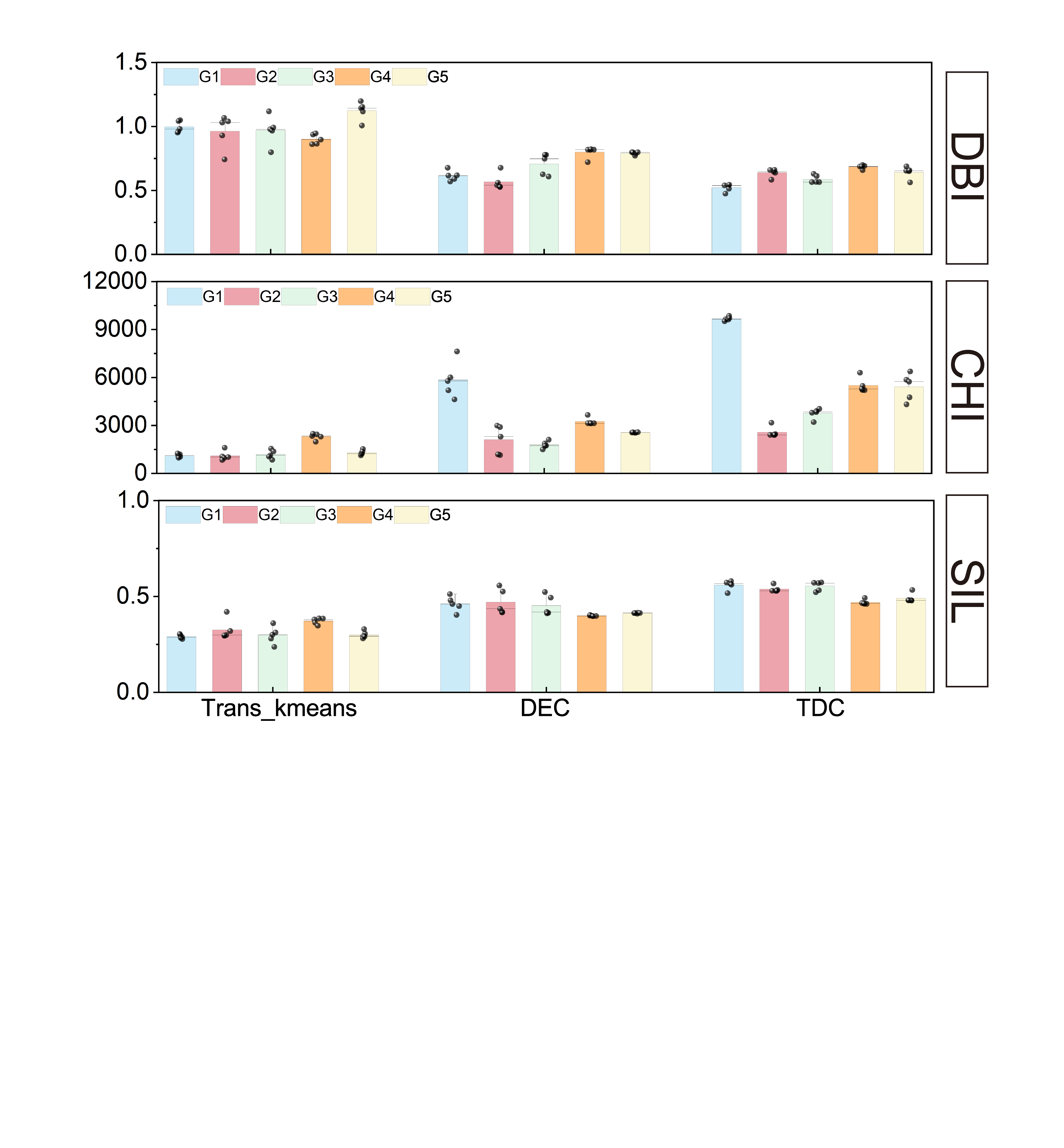


**Supplementary Fig. 27.** Performance evaluation of the TDC model based on EHL muscle data. The TDC models were evaluated using DBI, CHI, and SIL for MS classification ranging from G1 to G5, respectively. Compared to Trans-Kmeans (a combination of Transformer and K-means) and Deep Embedded Cluster (DEC) methods, the TDC model demonstrated superior performance in all grades of MS classification.


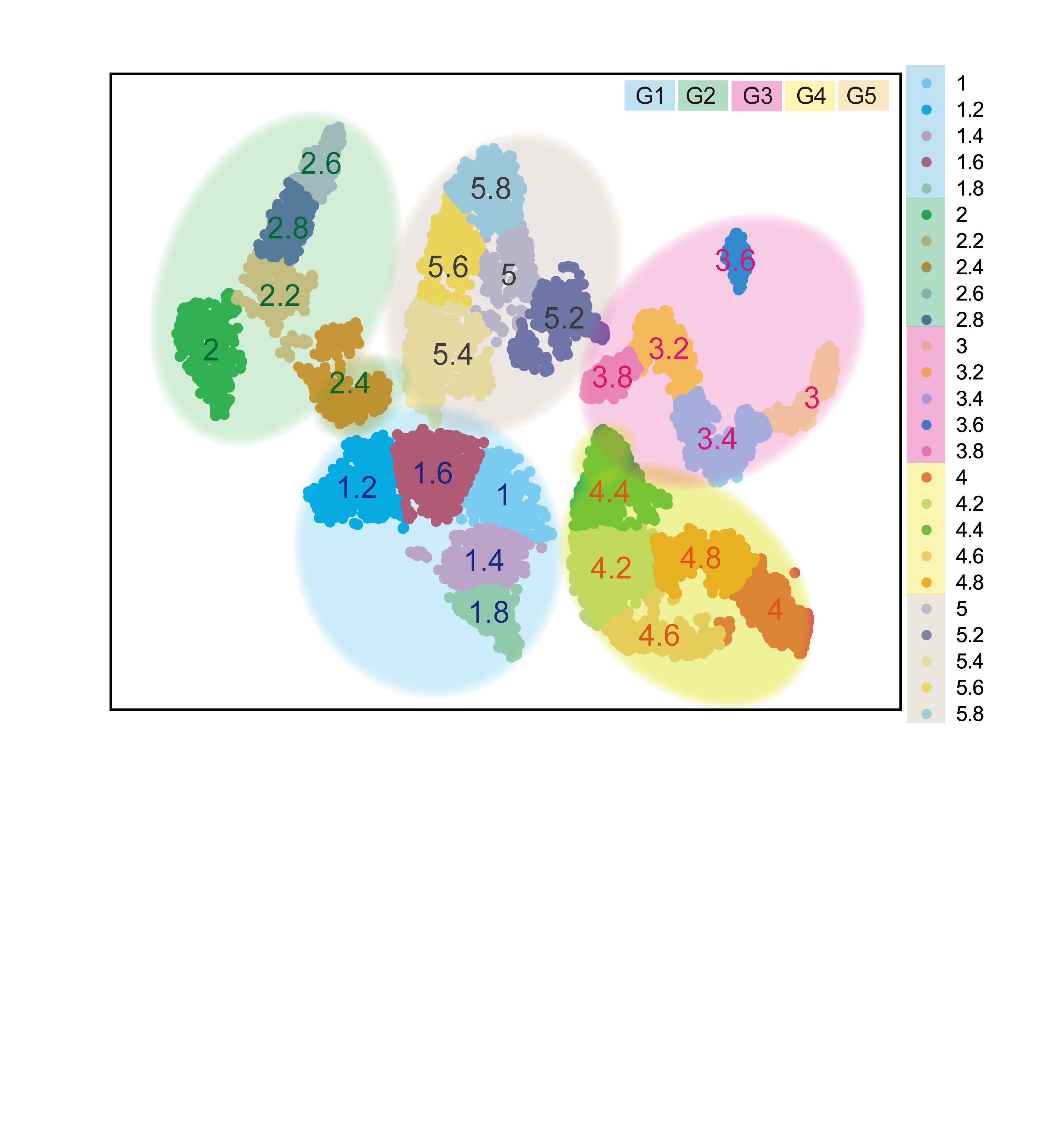
**Supplementary Fig. 28.** Refined grading characteristics and discrete point distribution of 25 MS levels of EHL based on the TDC model. The results showed that TDC effectively separated the five MS grades of EHL muscles, achieving a depth grading of 25 levels with minimal overlap between subclasses.


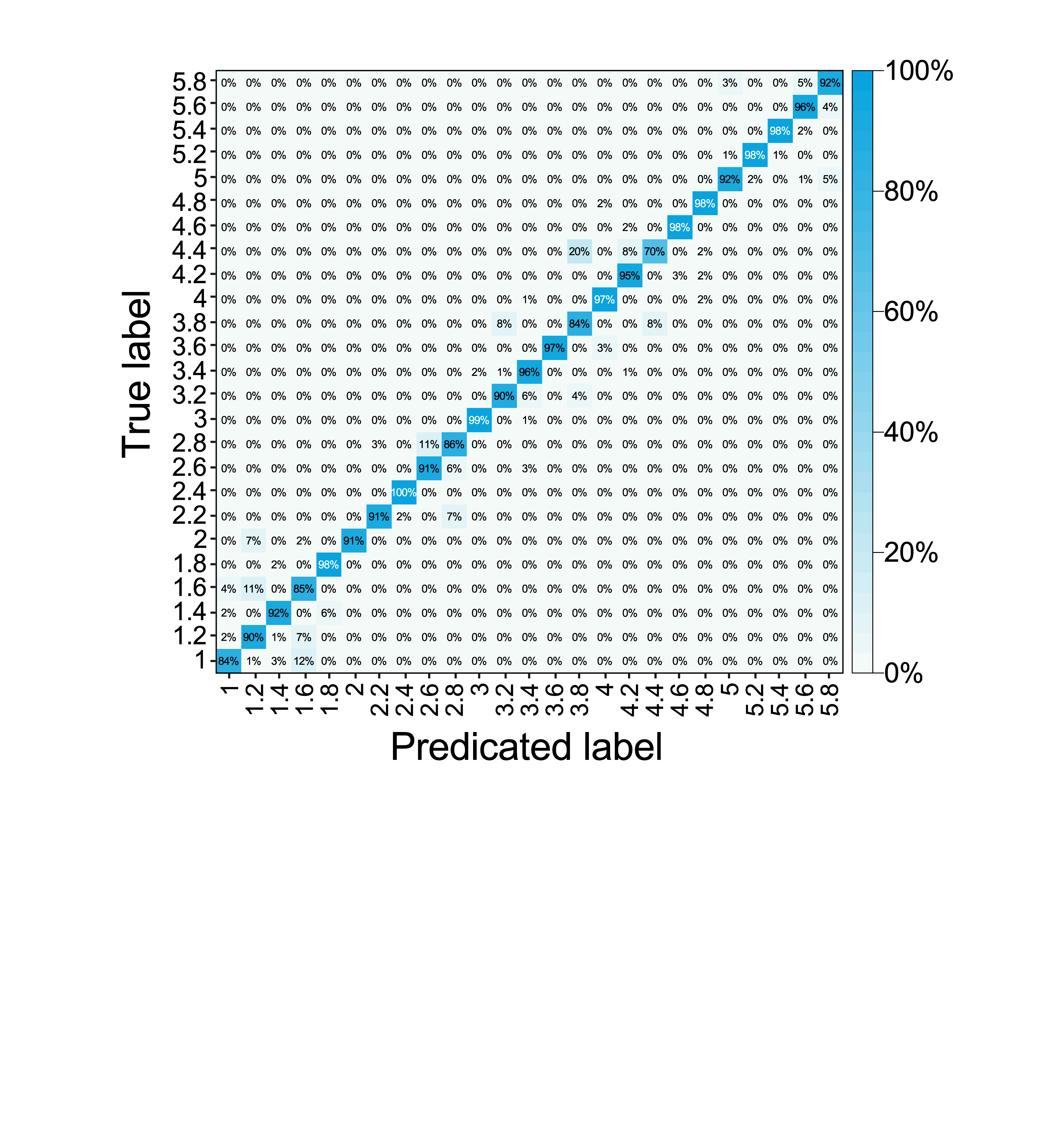


**Supplementary Fig. 29.** Confusion matrix classification of 25 grade of MS of the EHL muscle. The TDC model achieved a refined classification accuracy of ~ 92.3% for MS of the EHL muscle based on 30 patients.


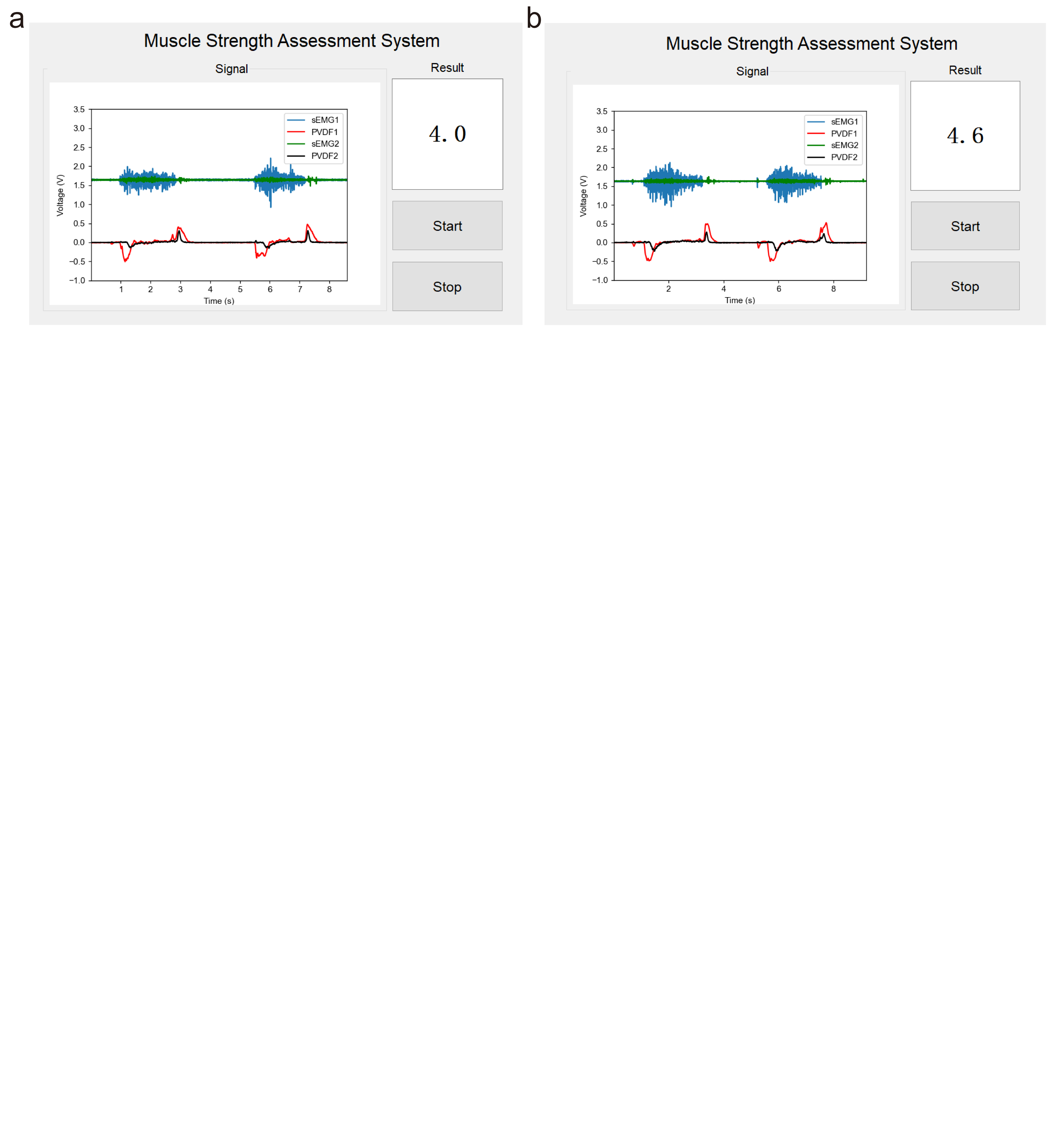
**Supplementary Fig. 30.** Clinical validation in patients with the same muscle grade. **a**, Based on the Tcnformer model, patients A (17) and B (22) were categorized into the 4^th^ level of MS. Subsequently, a finer-grained classification revealed that patient A had an MS level of 4.0, (**b**) while patient B had a level of 4.6, indicating that patient A exhibited weaker MS. Clinical validation further demonstrated that both patients A and B achieved a 4^th^ level of resistance against gravitational force, with patient B exhibiting superior resistance capabilities. This substantiates the effectiveness of our refined grading algorithm for patients with the same MS level.

**
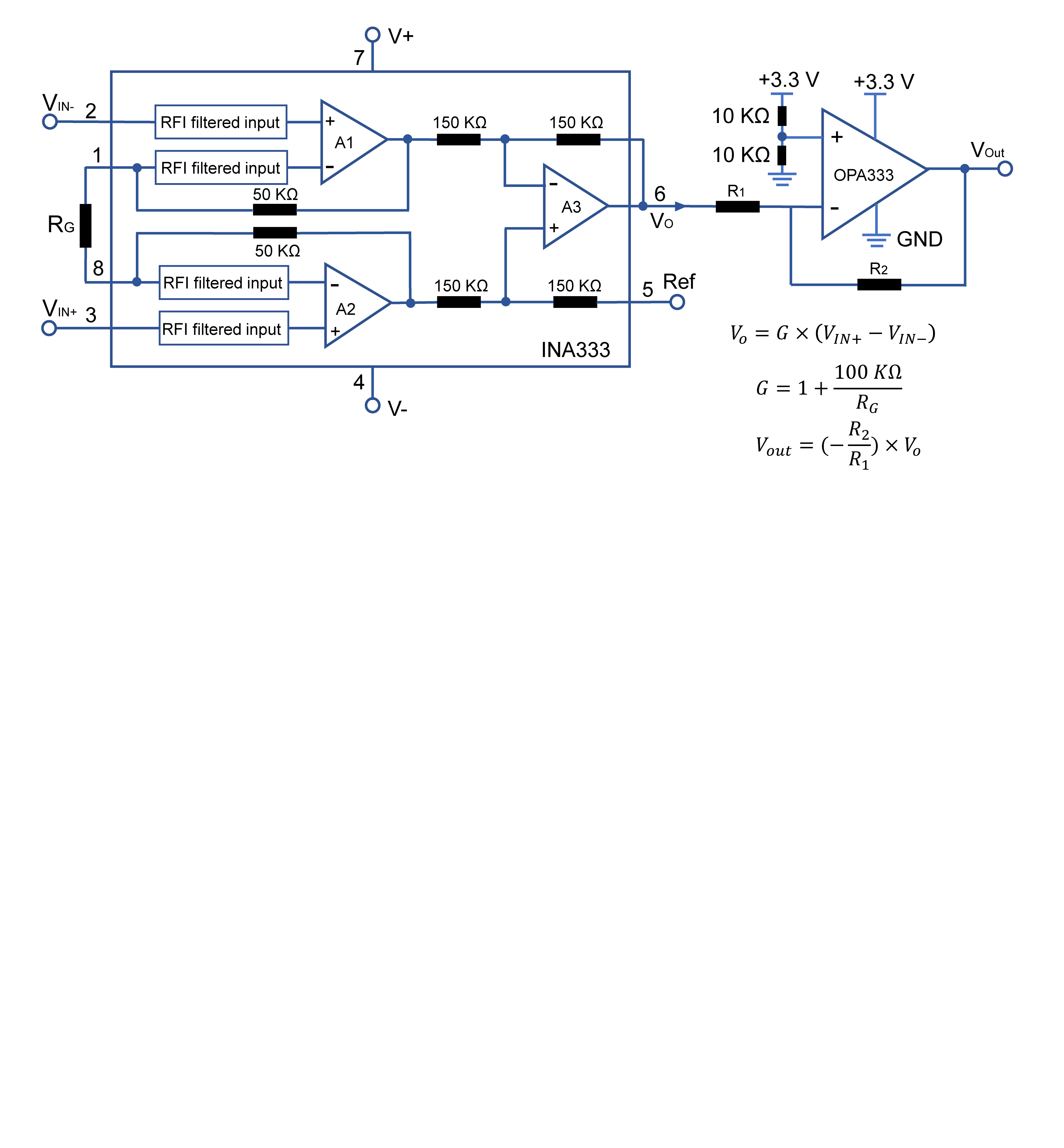
Supplementary Fig. 31.** Amplification gains of the sEMG circuit module. The amplification gain of the sEMG signal is determined by the reference resistor (R_G_) of the INA333 pre-stage op-amp and the ratio of the input and feedback resistors of the OPA333 post-stage op-amp, but limited by the op-amp supply voltage (3.3 V). The baseline of the sEMG signal is divided through two 10 KΩ divider resistors, held at 1/2 of the supply voltage (around 1.65 V).

**Supplementary Table 1.** The parameters of PVDF films

| Parameters | Structure 1 | Structure 2 | Unit |
| --- | --- | --- | --- |
| Dimensions of serpentine PVDF | 35×20 | 35×20 | mm |
| Film thickness | 100 | 100 | um |
| Piezo Strain Constant | 23 | 23 | d31 (10^-12^ C N^-1^) |
| Piezo Strain Constant | -33 | -33 | d33 (10^-12^ C N^-1^) |
| Piezo Stress Constant | 216 | 216 | g31 (Vm N^-1^) |
| Piezo Stress Constant | -330 | -330 | g33 (Vm N^-1^) |
| Dielectric Constant | 106-113 | 106-113 | 10^-12^ F m^-1^ |
| Relative Dielectric Constant | 12-13 | 12-13 |  |
| Young’ modulus | 2-4 | 2-4 | 10^9^ N m^-2^ |

**Supplementary Table 2.** The statistics of patients with different MS grades.

The abbreviations *LSS*, *LDH*, *DLS*, *F/M*, and *B* in the table represent the following:

LSS: lumbar spinal stenosis; LDH: lumbar disc herniation; DLS: degenerative lumbar spondylolisthesis; F: female; M: male; B: before surgery.

| Test date | Sample | Patients | Sex (F/M) | Age | TA(L) | EHL(L) | TA(R) | EHL(R) | Symptom | Diagnose | Before/After Surgery |
| --- | --- | --- | --- | --- | --- | --- | --- | --- | --- | --- | --- |
| 20220426 | 1 | Bed 31 of Bone 1 | M | 63 | 5 | 3 | 5 | 4、5 | Pain and numbness for fifteen years | LSS, L45 | B |
|  | 2 | Bed 22 of Bone 2 | F | 52 | 4 | 1 | 5 | 5 | Pain and numbness for three months | LSS, LDH | B |
|  | 3 | Bed 21 of Bone 2 | F | 61 | 5 | 5 | 4 | 4 | Right lower pain and numbness | LSS, LDH | B |
|  | 4 | Bed 5 of Bone 2 | M | 46 | 3 | 3 | 5 | 5 | Lumbar herniated disc | LSS, LDH | B |
| 20220602 | 5 | Bed 32 of Bone 1 | M | 64 | 5 | 4 | 5 | 5 | Low back pain for two years | LSS | One day after surgery |
|  | 6 | Bed 2 of Bone 2 | F | 62 | 4 | 4 | 3 | 3 | Low back pain for twenty years | LSS, L34 | One day after surgery |
|  | 7 | Bed 4 of Bone 2 | F | 73 | 5 | 5 | 1 | 4 | Low back pain for ten years | LSS | On the day of surgery |
|  | 8 | Bed 39 of Bone 2 | F | 71 | 5 | 4 | 4 | 4 | Low back pain for a year | DLS | On the day of surgery |
| 20220705 | 9 | Bed 2 of Bone 2 | F | 67 | 4 | 3 | 5 | 4 | Low back pain for six years | LSS, LDH | B |
|  | 10 | Bed 13 of Bone 2 | M | 72 | 5 | 4 | 5 | 4 | Numbness in both feet for a year | LSS | B |
|  | 11 | Bed 8 of Bone 2 | F | 50 | 4 | 5 | 5 | 5 | Low back pain for two years, limp for six months | LSS, LDH | B |
|  | 12 | Bed 17 of Bone 2 | F | 54 | 5 | 4 | 5 | 5 | Low back pain for four years | LSS (L45), LDH (L3-L5) | B |
|  | 13 | Bed 30 of Bone 1 | F | 59 | 5 | 5 | 5 | 3 | Numbness and pain in right lower limb for a week | LDH, L3-L5 | B |
| Test date | Sample | Patients | Sex (F/M) | Age | TA(L) | EHL(L) | TA(R) | EHL(R) | Symptom | Diagnose | Before/After Surgery |
| 20220712 | 14 | Bed 4 of Bone 2 | F | 62 | 5 | 3 | 5 | 5 | Low back pain for twenty years | LSS, LDH, L45 | B |
|  | 15 | Bed 7 of Bone 2 | M | 35 | 5 | 5 | 5 | 4 | Low back pain for ten years | LDH | B |
|  | 16 | Bed 21 of Bone 1 | F | 44 | 5 | 5 | 5 | 3 | Lumbosacral pain for a year | LSS, LDH, L45 | B |
| 20220719 | 17 | Bed 3 of Bone 2 | F | 61 | 5 | 4 | 5 | 5 | Low back pain with left lower limb pain for a year | LSS, LDH, L45 | B |
|  | 18 | Bed 5 of Bone 2 | M | 63 | 5 | 5 | 1 | 0 | Low back pain with right hip and right lower limb pain for two years | LSS | B |
| 20220726 | 19 | Bed 4 of Bone 1 | F | 47 | 5 | 4 | 5 | 5 | Low back pain and left lower limb pain for ten days | LDH | B |
|  | 20 | Bed 40 of Bone 1 | F | 66 | 5 | 5 | 4 | 4 | Numbness in both calves for over ten years | LSS, L4 | B |
|  | 21 | Bed 13 of Bone 2 | F | 74 | 4 | 4 | 3 | 4 | Low back pain and bilateral lower limb pain for eight years | LSS, L3-5, L4 | B |
|  | 22 | Bed 20 of Bone 2 | M | 59 | 5 | 4 | 5 | 5 | Low back pain for two years, lameness for a year | LSS, LDH (L12-L51) | B |
| 20220805 | 23 | Bed 2 of Bone 2 | F | 52 | 5 | 5 | 1 | 2 | Low back pain and numbness | LSS, L2-4 | One day after surgery |
|  | 24 | Bed 20 of Bone 2 | M | 27 | 5 | 3 | 5 | 5 | Low back pain, numbness and weakness | LDH | B |
| Test date | Sample | Patients | Sex (F/M) | Age | TA(L) | EHL(L) | TA(R) | EHL(R) | Symptom | Diagnose | Before/After Surgery |
| 20220816 | 25 | Bed 35 of Bone 2 | M | 36 | 5 | 5 | 3 | 3 | Left lower limb pain for five years | LDH, L45 | Five days after surgery |
| 20220913 | 26 | Bed 40 of Bone 1 | M | 44 | 5 | 5 | 3 | 1 | Soreness of the right lower limb, numbness and weakness of the right foot for a month | LDH, L3-L5 | Two days after surgery |
| 20220916 | 27 | Bed 5 of Bone 4 | M | 36 | 2 | 1 | 2 | 1 | Low back pain for six months with bilateral lower limb pain and numbness for five months | LDH | One day after surgery |
| 20220920 | 28 | Bed 27 of Bone 1 | F | 66 | 5 | 5 | 5 | 2 | Low back pain for a year, right lower limb limp for two months | LSS, LDH | B |
| 20220929 | 29 | Bed 39 of Bone 2 | M | 68 | 1 | 1 | 1 | 1 | Low back pain with right lower limb pain for two months | LSS | B |
| 20221013 | 30 | Bed 35 of Bone 1 | M | 49 | 5 | 5 | 5 | 2 | Low back pain with numbness in the right lower limb for ten years | LDH | B |
| 20221024 | 31 | Bed 14 of Bone 1 | M | 23 | 5 | 5 | 2 | 2 | Low back pain with numbness in the right lower limb for nine days | LDH, L5-S1 | B |
| 20221026 | 32 | Bed 26 of Bone 2 | F | 71 | 2 | 1 | 5 | 5 | Low back pain with numbness in both lower limbs and lameness for ten years | LSS, LDH | B |
| 20221108 | 33 | Bed 1 of Bone 2 | F | 75 | 3 | 2 | 5 | 5 | Low back pain for twenty years | LDH, LSS | B |

**Supplementary Table 3.** The Pearson correlation coefficients of coupled signals in Fig.3c and Fig. 3d when subjects performed the same repetitive actions. (2 patients were collected, both P＜0.001)

By obtaining the root mean square (RMS) of the sEMG signal under each set of movements, combining it with the corresponding piezoelectric signal, and then analyzing the Pearson correlation coefficient of the two group signals under the same movement^9, 10^.

|  | Pearson CorrCoef | |
| --- | --- | --- |
| Fig. 3c | 0.981±0.03 | |
| Fig. 3d | 0.871±0.02 (TA) | 0.865±0.03 (EHL) |

**Supplementary Table 4.** Pearson correlation coefficients of coupled signals when performing the same movement in patients with different MS grades. (1 patient per level, 5 patients in total, P<0.001)

| MS Levels | Pearson CorrCoef |
| --- | --- |
| 1 | 0.913±0.10 |
| 2 | 0.868±0.06 |
| 3 | 0.884±0.03 |
| 4 | 0.893±0.09 |
| 5 | 0.877±0.03 |

**Supplementary Table 5.** Pearson correlation coefficients of the coupled signals when performing the same movement in patients with the same MS grades. (3 patients per level, 15 patients in total, P＜0.001)

| MS levels | Pearson CorrCoef (EHL) | Pearson CorrCoef (TA) |
| --- | --- | --- |
| 1 | 0.856±0.11 | 0.768±0.16 |
| 2 | 0.737±0.10 | 0.724±0.14 |
| 3 | 0.745±0.16 | 0.850±0.11 |
| 4 | 0.780±0.15 | 0.735±0.18 |
| 5 | 0.836±0.12 | 0.784±0.11 |

**Supplementary Table 6.** Clinical MS Refined Grading Scale^11, 12^.

| Grade | Descriptions |
| --- | --- |
| 0 | No contractions felt in the muscle |
| 1 | Tendon becomes prominent or feeble contraction felt in the muscle, but no visible movement of the part |
| 2- | Moves through partial range of motion |
| 2 | Moves through complete range of motion |
| 2+ | Moves through partial range of motion against gravity or moves through complete range of motion gravity eliminated and holds against pressure |
| 3- | Gradual release from test position |
| 3 | Holds test position against gravity (no added resistance) |
| 3+ | Holds test position against slight resistance |
| 4- | Holds test position against slight to moderate resistance |
| 4 | Holds test position against moderate resistance |
| 4+ | Holds test position against moderate to strong  resistance |
| 5 | Holds test position against strong resistance |

**References**

1. Ge, W., et al. Nanocellulose/LiCl systems enable conductive and stretchable electrolyte hydrogels with tolerance to dehydration and extreme cold conditions. *Chem. Eng. J.* **408**, 127306 (2021).

2. Bao, D. et al. An anti-freezing hydrogel based stretchable triboelectric nanogenerator for biomechanical energy harvesting at sub-zero temperature. *J. Mater. Chem. A* **8**, 13787-13794 (2020).

3. Wang, S. et al. Strong, tough, ionic conductive, and freezing-tolerant all-natural hydrogel enabled by cellulose-bentonite coordination interactions. *Nat. Commun.* **13**, 3408 (2022).

4. Sui, X. et al. Ionic conductive hydrogels with long-lasting antifreezing, water retention and self-regeneration abilities. *Chem. Eng. J.* **419**, 129478 (2021).

5. Tang, X. et al. Exploiting synergistic effect of CO/NO gases for soft tissue transplantation using a hydrogel patch. *Nat. Commun.* **14**, 2417 (2023).

6. Jia, Y. et al. Hydrogel dressing integrating FAK inhibition and ROS scavenging for mechano-chemical treatment of atopic dermatitis. *Nat. Commun.* **14**, 2478 (2023).

7. Liu, D. et al. Active-Matrix Sensing Array Assisted with Machine-Learning Approach for Lumbar Degenerative Disease Diagnosis and Postoperative Assessment. *Adv. Funct. Mater.* **32**, 2113008 (2022).

8. Wang, P. et al. Understanding Convolution for Semantic Segmentation. *2018 IEEE Winter Conf. Appl. Comput. (WACV)*, 1451-1460 (2017).

9. Zhang, S., et al. Muscle Strength Assessment System Using sEMG-Based Force Prediction Method for Wrist Joint. *J. Med. Biol. Eng.* **36**, 121-131 (2016).

10. Watanabe, K., et al. Relationships between muscle strength and multi-channel surface EMG parameters in eighty-eight elderly. *Eur. Rev. Aging and Phy. Act.* **15**, 1-10 (2018).

11. Bohannon, R. W., et al Considerations and Practical Options for Measuring Muscle Strength: A Narrative Review. *BioMed Res. Int.* 8194537 (2019).

12. Vanhoutte, E. K., et al. Modifying the Medical Research Council grading system through Rasch analyses. *Brain*, **135**, 1639-1649 (2012).
