# Supplementary figures and images for: Deep Learning Model Coupling Wearable Bioelectric and Mechanical Sensors for Refined Muscle Strength Assessment
Source: Research (Wash D C). 2024 May 23;7:0366. doi: 10.34133/research.0366 (PMC11112600; doi:10.34133/research.0366)

**a**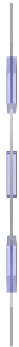

Side view

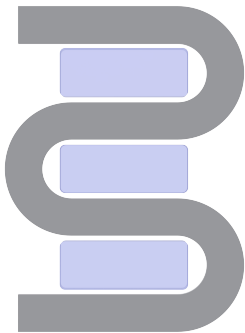

Front view

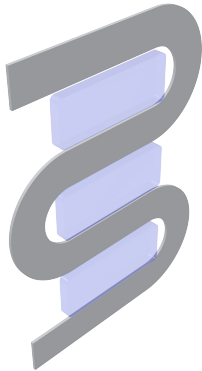

Isometric view

**b**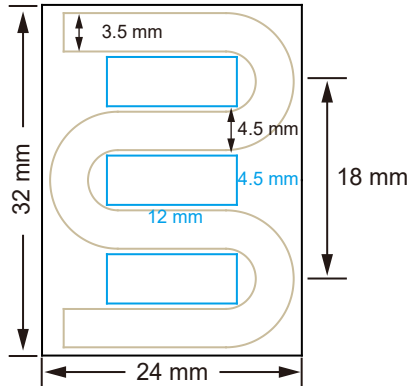

Supplement: Supplementary 1 — Figs. S1 to S31 Movies S1 to S3 Tables S1 to S6 [file research.0366.f1.zip › SI Figure/Fig. S1.pdf]

a

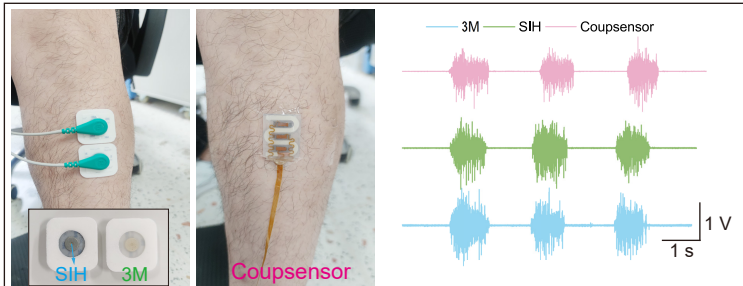

d

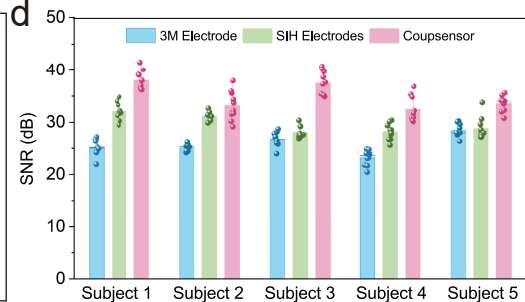

b

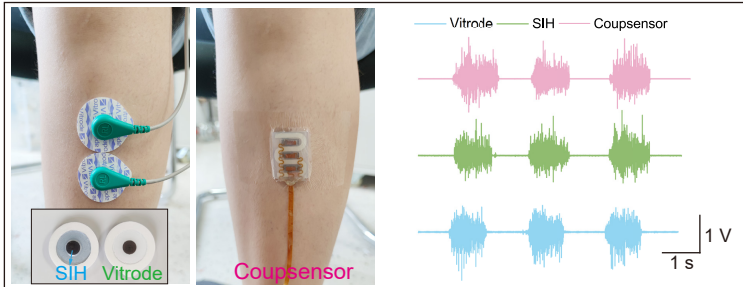

e

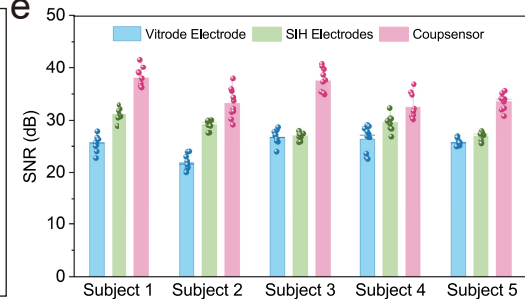

c

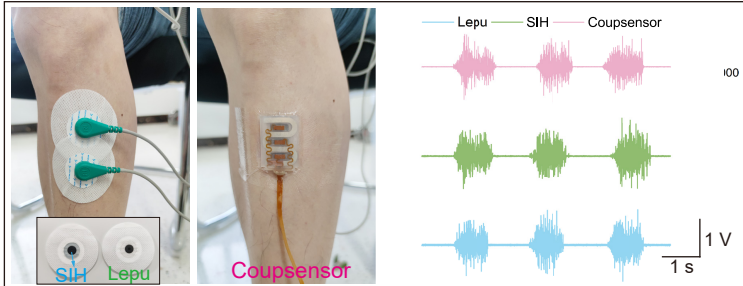

f

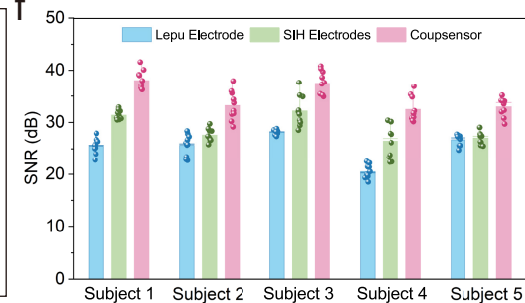

Supplement: Supplementary 1 — Figs. S1 to S31 Movies S1 to S3 Tables S1 to S6 [file research.0366.f1.zip › SI Figure/Fig. S11.pdf]

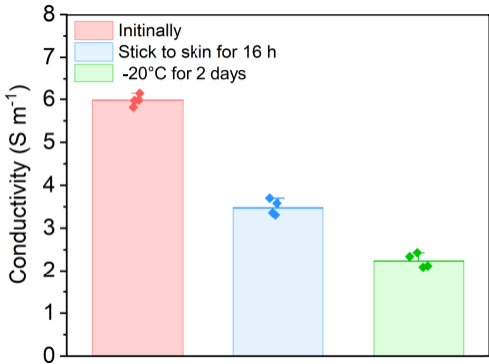

Supplement: Supplementary 1 — Figs. S1 to S31 Movies S1 to S3 Tables S1 to S6 [file research.0366.f1.zip › SI Figure/Fig. S12.pdf]

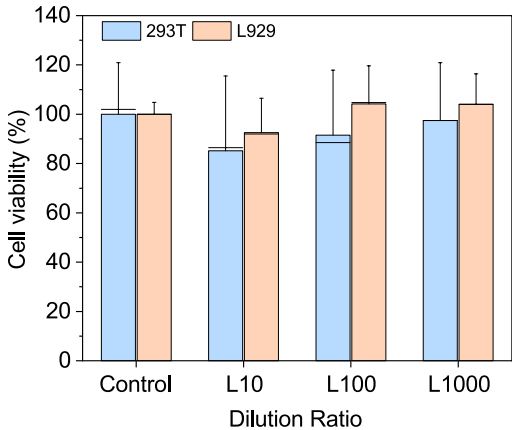

Supplement: Supplementary 1 — Figs. S1 to S31 Movies S1 to S3 Tables S1 to S6 [file research.0366.f1.zip › SI Figure/Fig. S13.pdf]

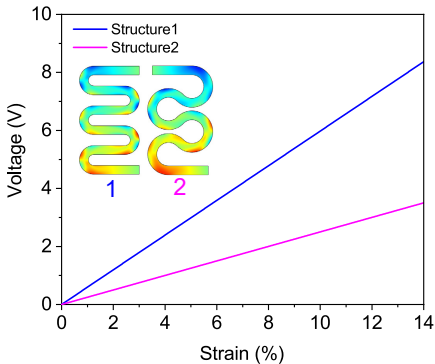

Supplement: Supplementary 1 — Figs. S1 to S31 Movies S1 to S3 Tables S1 to S6 [file research.0366.f1.zip › SI Figure/Fig. S14.pdf]

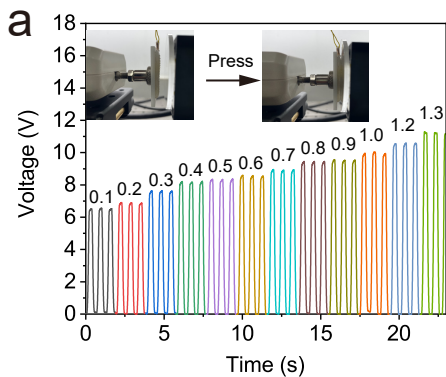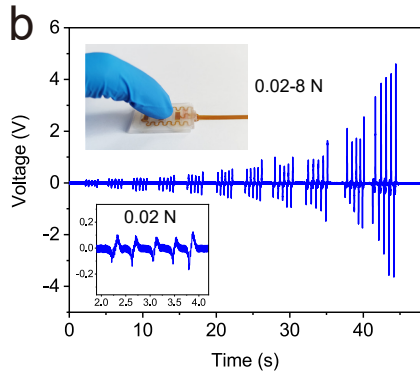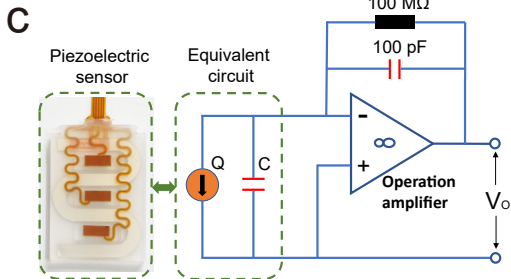

Supplement: Supplementary 1 — Figs. S1 to S31 Movies S1 to S3 Tables S1 to S6 [file research.0366.f1.zip › SI Figure/Fig. S15.pdf]

**a**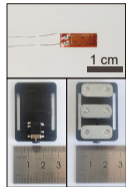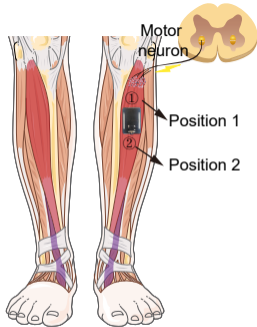**b**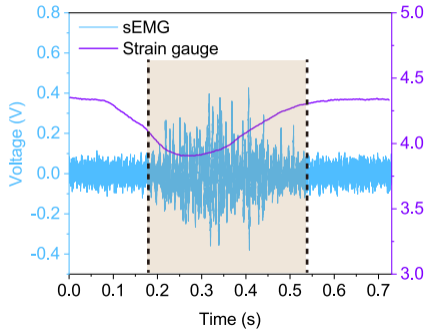**c**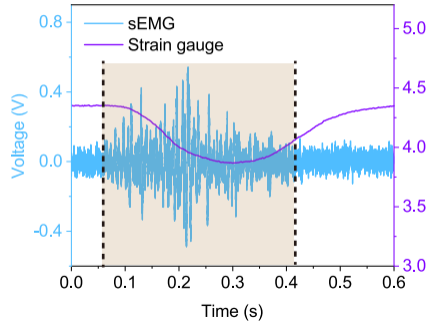

Supplement: Supplementary 1 — Figs. S1 to S31 Movies S1 to S3 Tables S1 to S6 [file research.0366.f1.zip › SI Figure/Fig. S17.pdf]

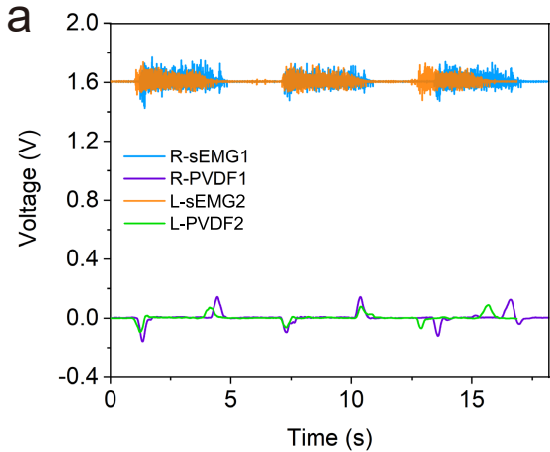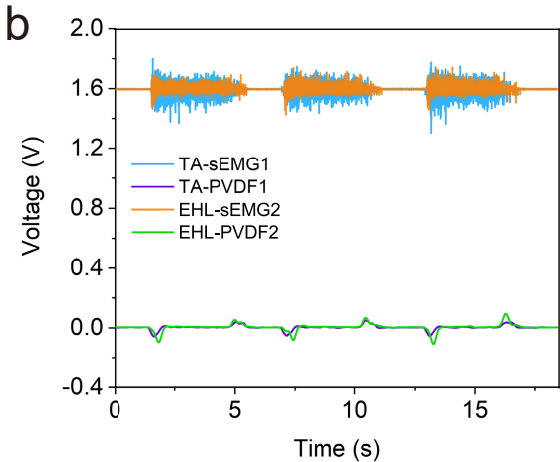

Supplement: Supplementary 1 — Figs. S1 to S31 Movies S1 to S3 Tables S1 to S6 [file research.0366.f1.zip › SI Figure/Fig. S18.pdf]

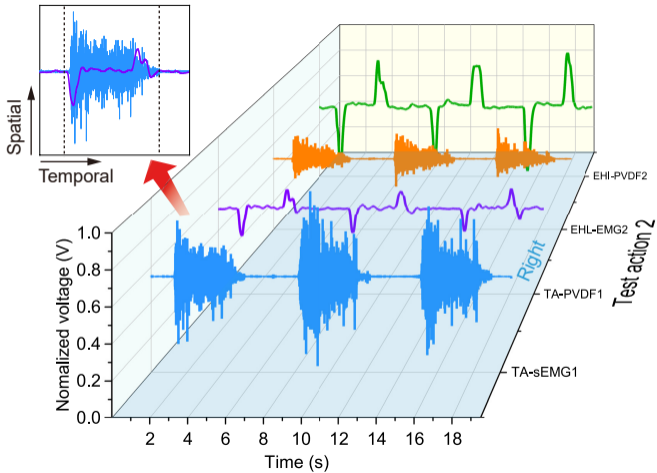

Supplement: Supplementary 1 — Figs. S1 to S31 Movies S1 to S3 Tables S1 to S6 [file research.0366.f1.zip › SI Figure/Fig. S19.pdf]

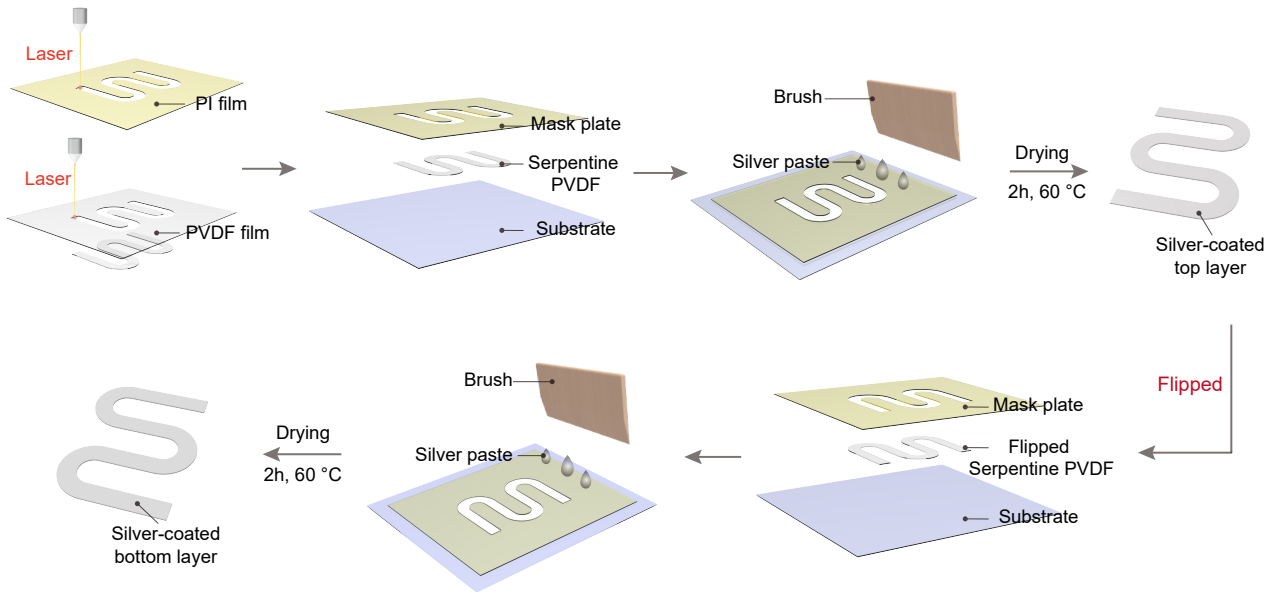

Supplement: Supplementary 1 — Figs. S1 to S31 Movies S1 to S3 Tables S1 to S6 [file research.0366.f1.zip › SI Figure/Fig. S2.pdf]

**a**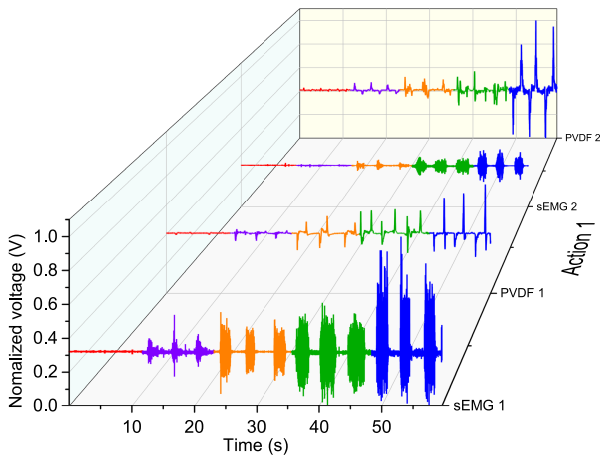**b**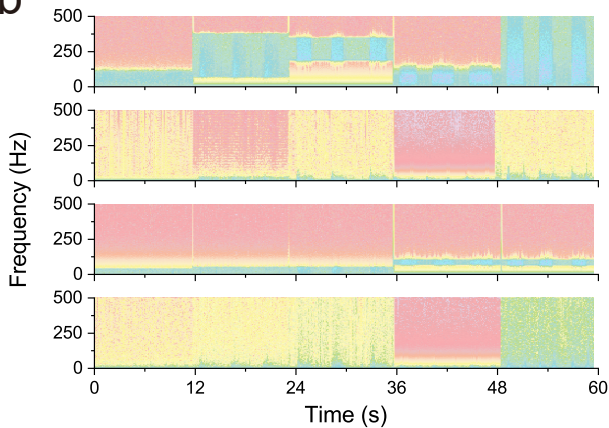

Supplement: Supplementary 1 — Figs. S1 to S31 Movies S1 to S3 Tables S1 to S6 [file research.0366.f1.zip › SI Figure/Fig. S20.pdf]

**a**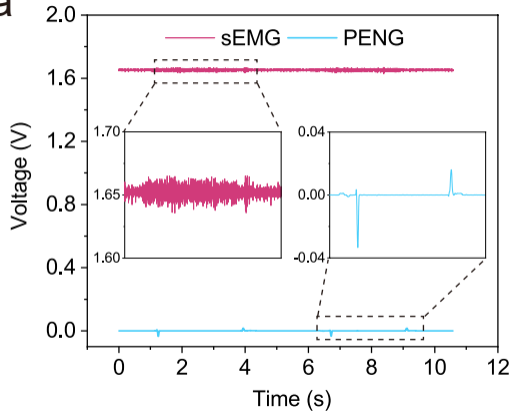**b**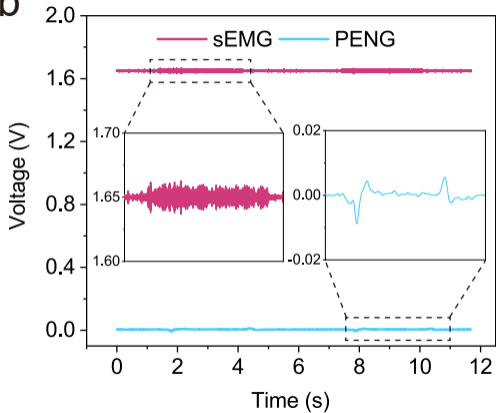

Supplement: Supplementary 1 — Figs. S1 to S31 Movies S1 to S3 Tables S1 to S6 [file research.0366.f1.zip › SI Figure/Fig. S21.pdf]

a

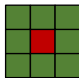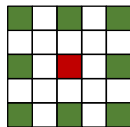

Convolution kernel

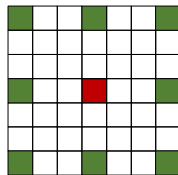

b

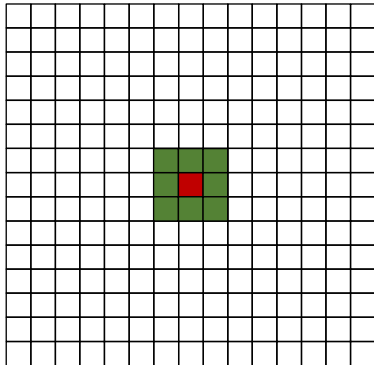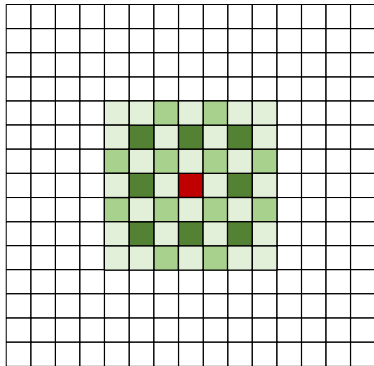

Receptive field

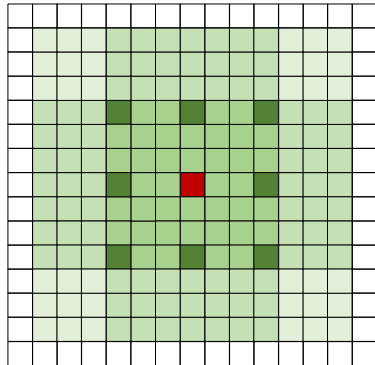

Supplement: Supplementary 1 — Figs. S1 to S31 Movies S1 to S3 Tables S1 to S6 [file research.0366.f1.zip › SI Figure/Fig. S23.pdf]

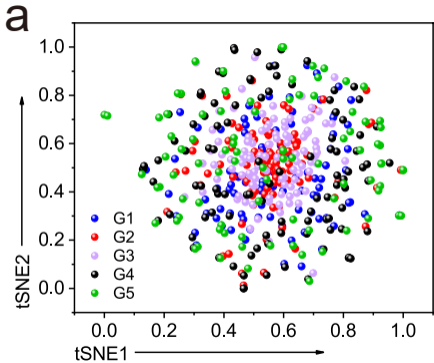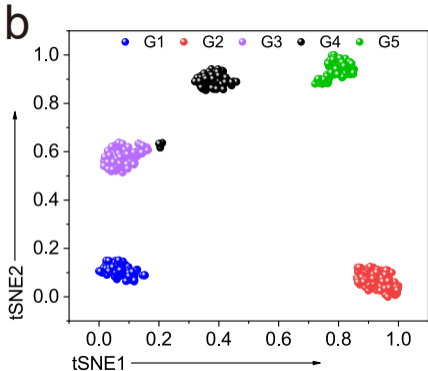

Supplement: Supplementary 1 — Figs. S1 to S31 Movies S1 to S3 Tables S1 to S6 [file research.0366.f1.zip › SI Figure/Fig. S25.pdf]

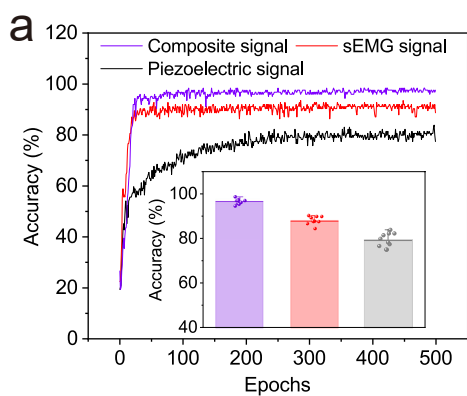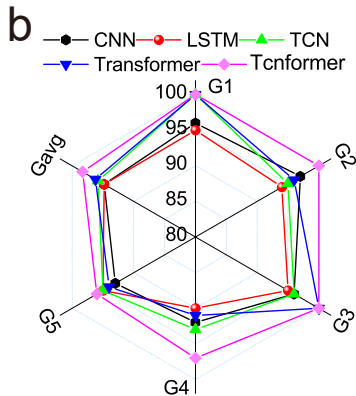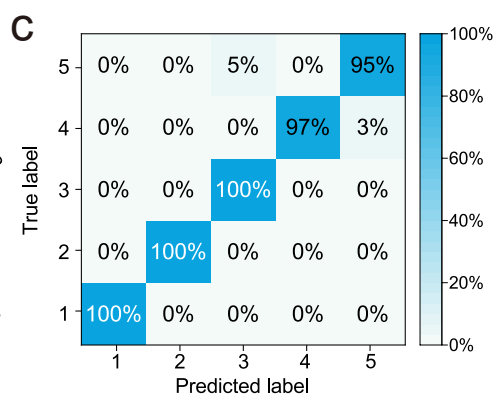

Supplement: Supplementary 1 — Figs. S1 to S31 Movies S1 to S3 Tables S1 to S6 [file research.0366.f1.zip › SI Figure/Fig. S26.pdf]

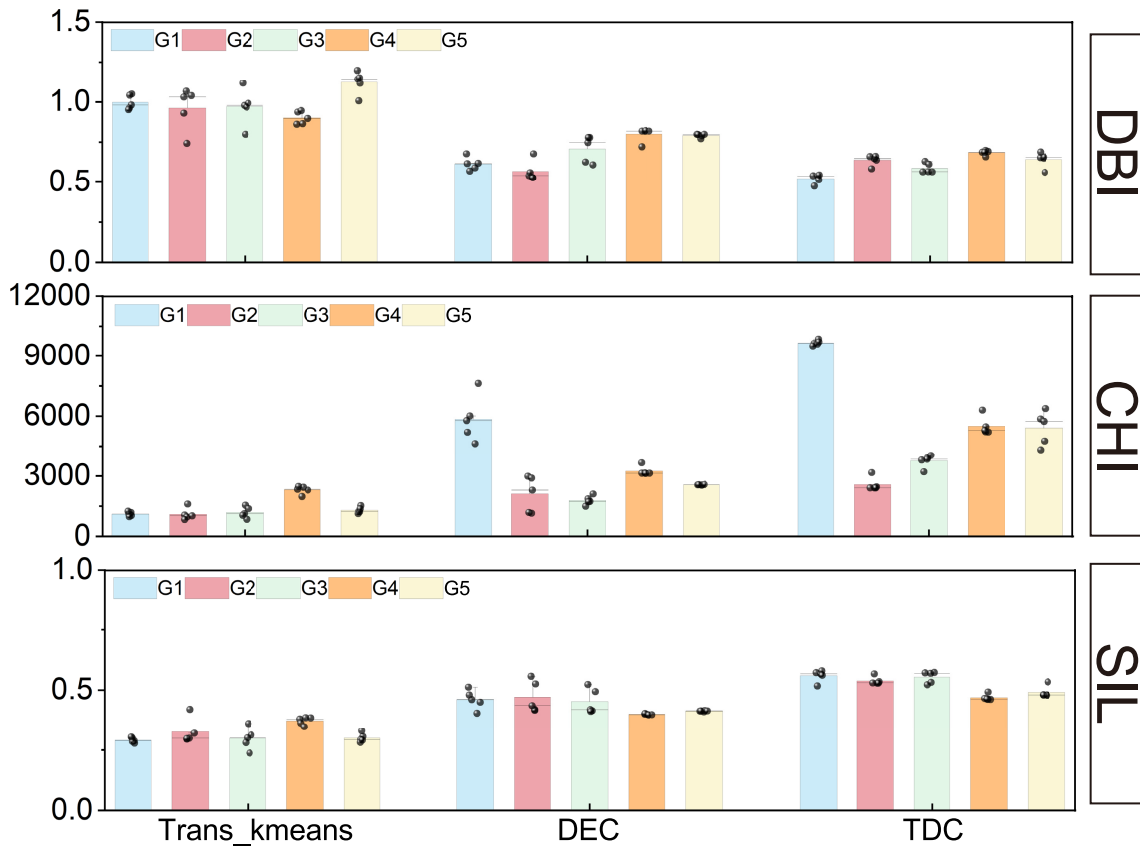

Supplement: Supplementary 1 — Figs. S1 to S31 Movies S1 to S3 Tables S1 to S6 [file research.0366.f1.zip › SI Figure/Fig. S27.pdf]

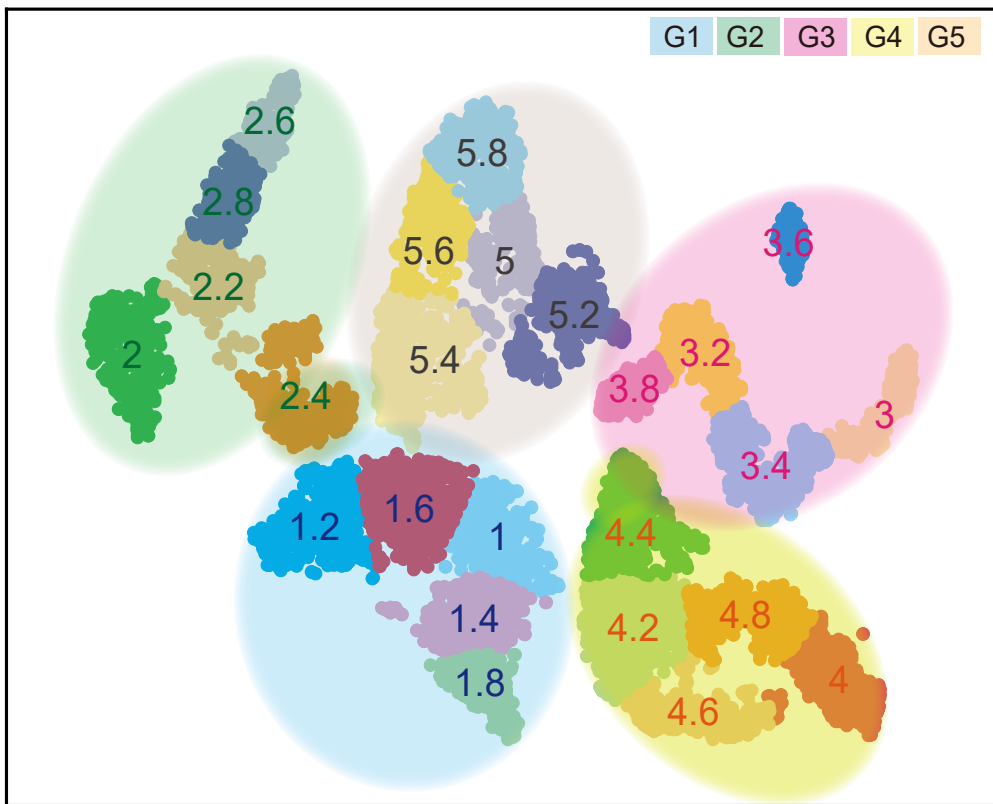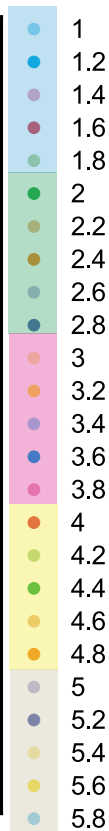

Supplement: Supplementary 1 — Figs. S1 to S31 Movies S1 to S3 Tables S1 to S6 [file research.0366.f1.zip › SI Figure/Fig. S28.pdf]

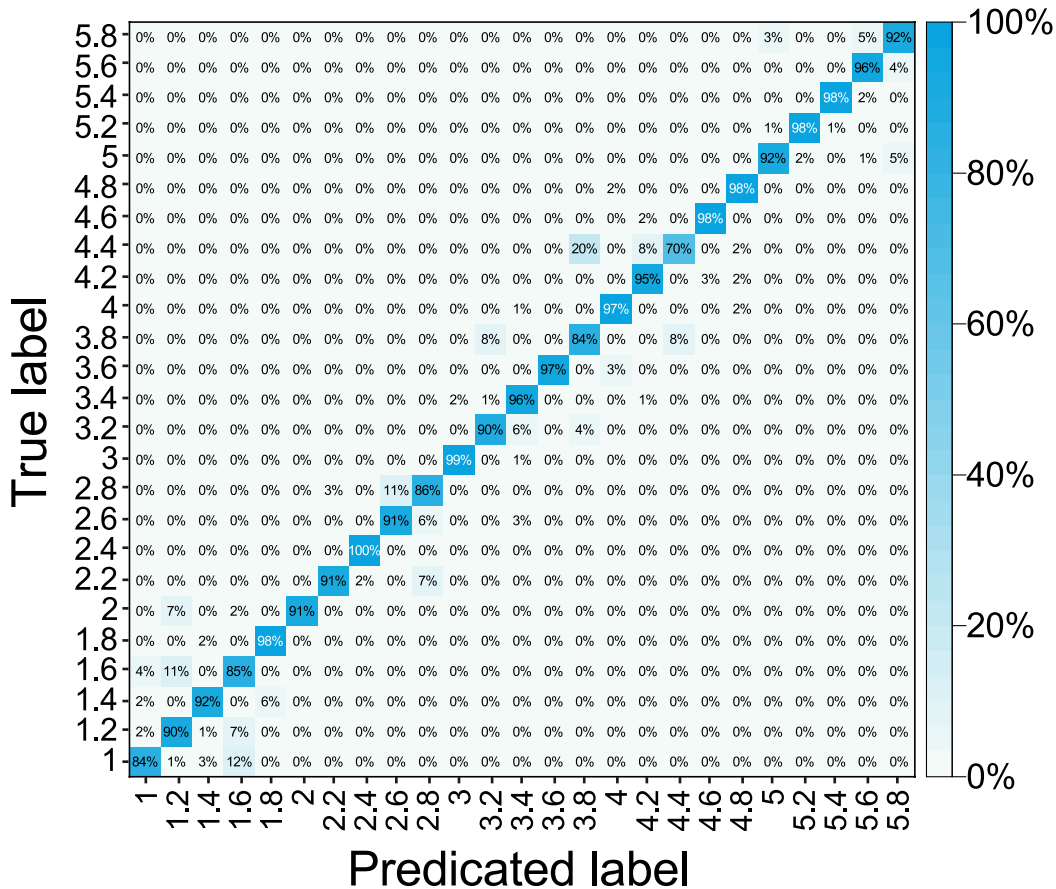

Supplement: Supplementary 1 — Figs. S1 to S31 Movies S1 to S3 Tables S1 to S6 [file research.0366.f1.zip › SI Figure/Fig. S29.pdf]

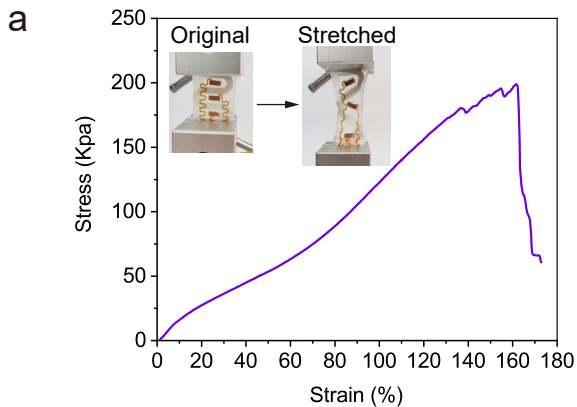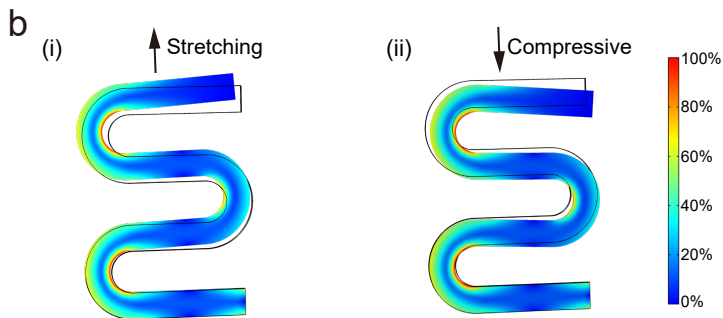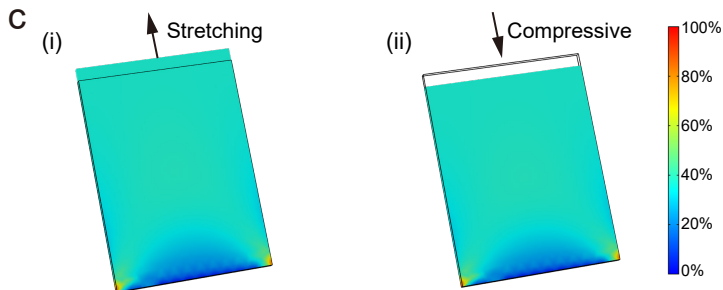

Supplement: Supplementary 1 — Figs. S1 to S31 Movies S1 to S3 Tables S1 to S6 [file research.0366.f1.zip › SI Figure/Fig. S3.pdf]

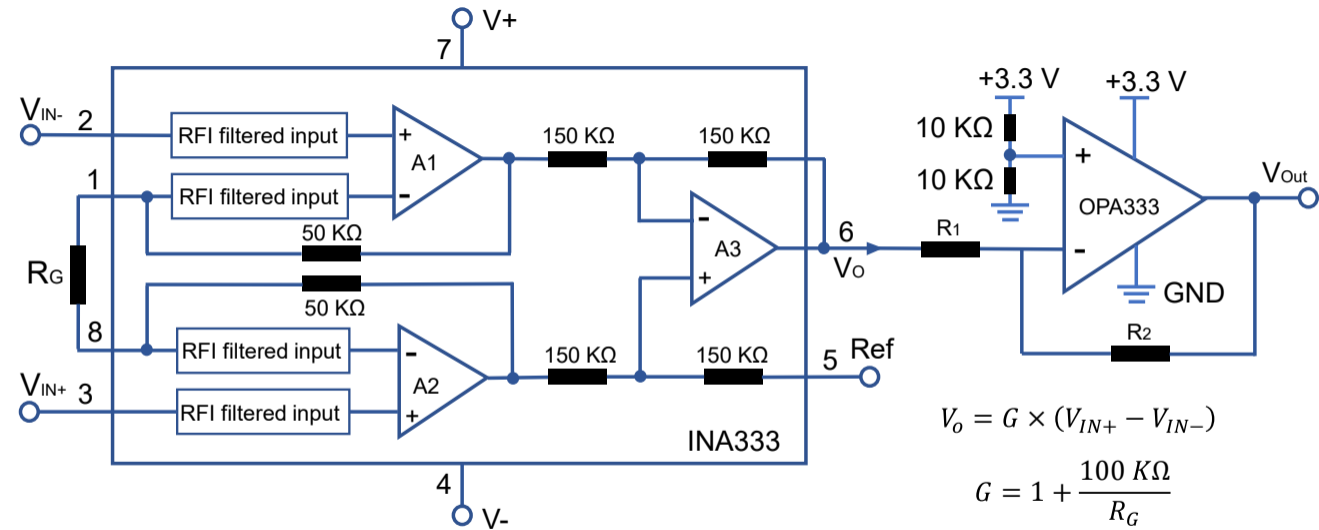

$$V_o = G \times (V_{IN+} - V_{IN-})$$

$$G = 1 + \frac{100 \text{ K}\Omega}{R_G}$$

$$V_{out} = \left(-\frac{R_2}{R_1}\right) \times V_o$$

Supplement: Supplementary 1 — Figs. S1 to S31 Movies S1 to S3 Tables S1 to S6 [file research.0366.f1.zip › SI Figure/Fig. S31.pdf]

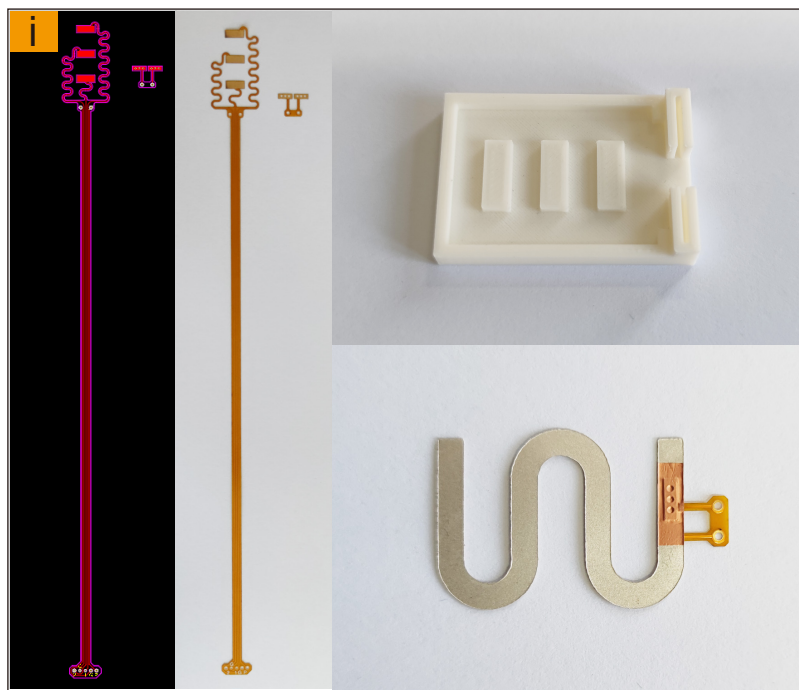

Component parts

Ecoflex

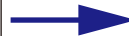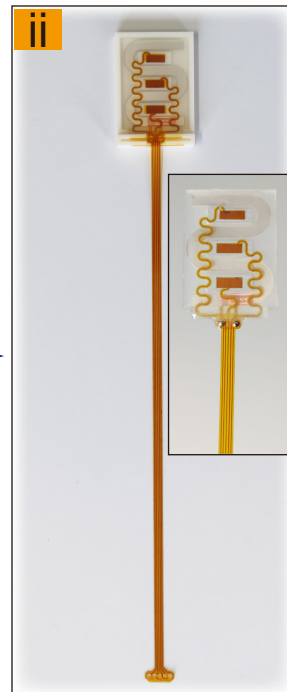

Integrated Circuit

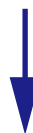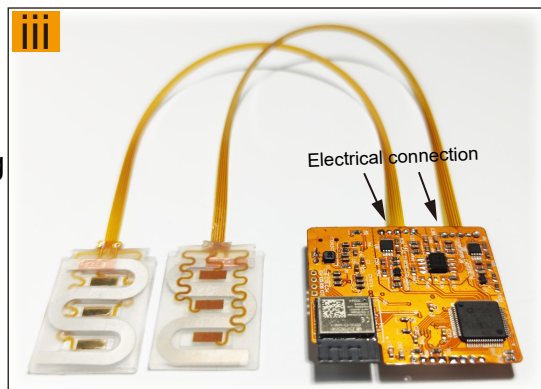

Packaging

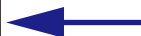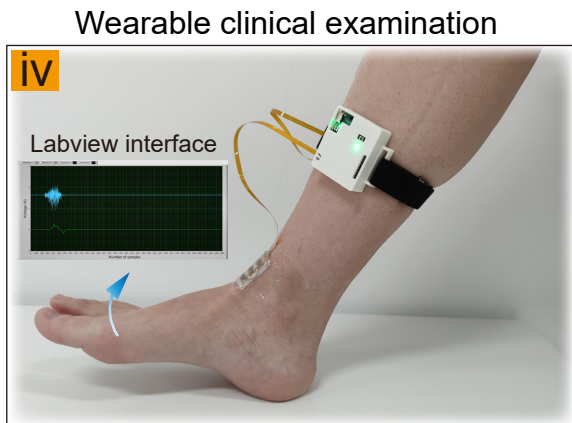

Wearable clinical examination

Supplement: Supplementary 1 — Figs. S1 to S31 Movies S1 to S3 Tables S1 to S6 [file research.0366.f1.zip › SI Figure/Fig. S4.pdf]

a

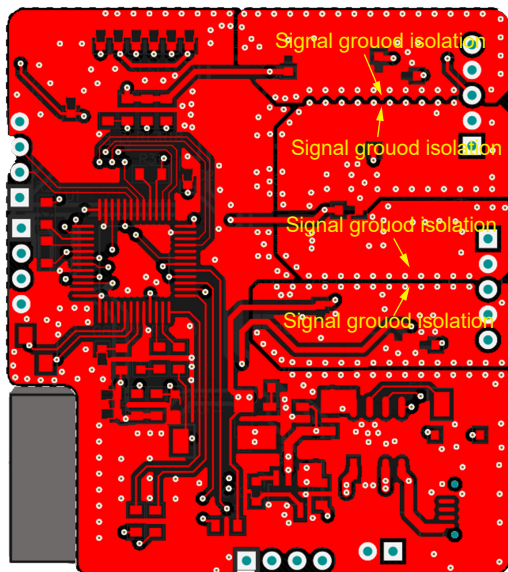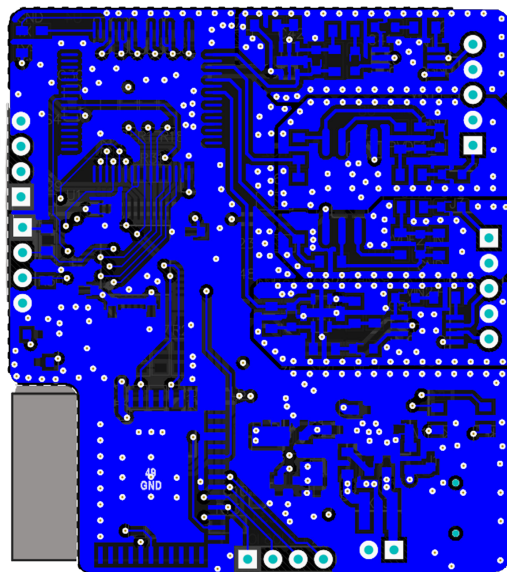

b

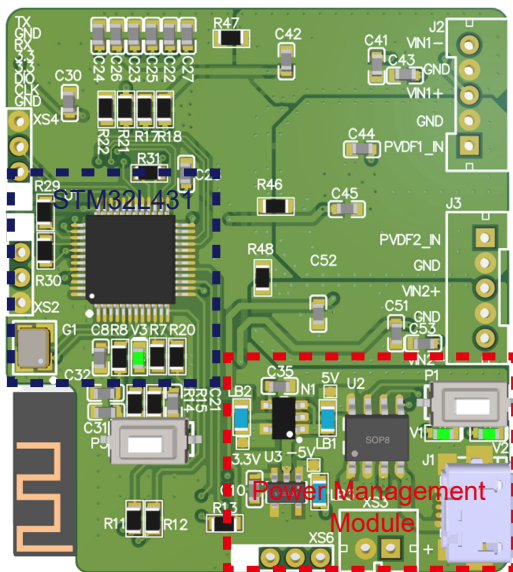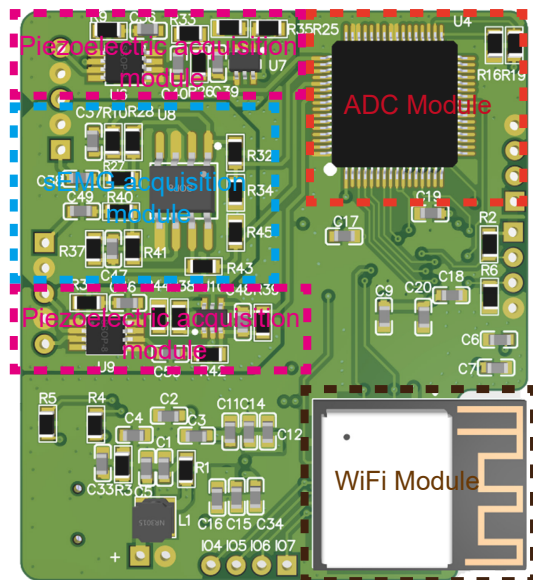

Supplement: Supplementary 1 — Figs. S1 to S31 Movies S1 to S3 Tables S1 to S6 [file research.0366.f1.zip › SI Figure/Fig. S6.pdf]

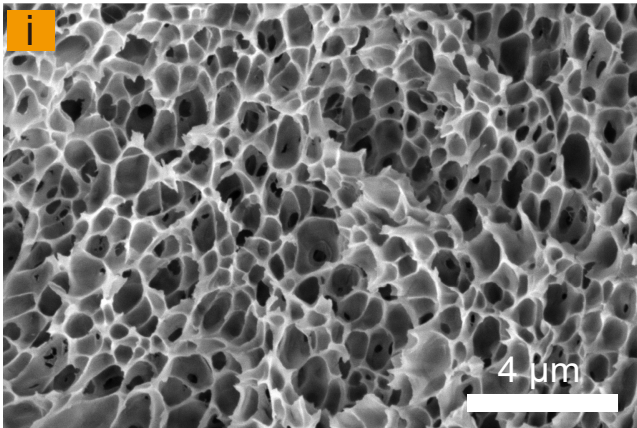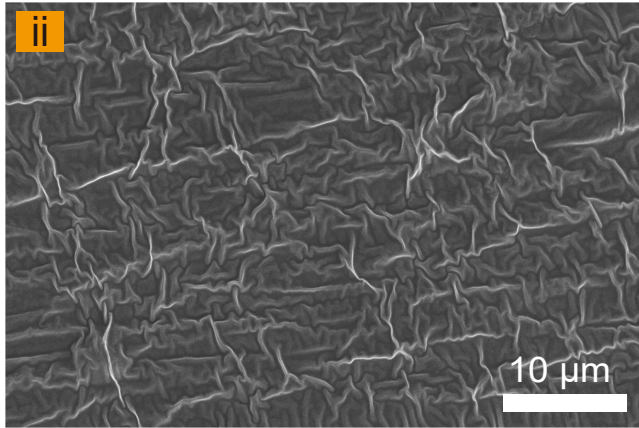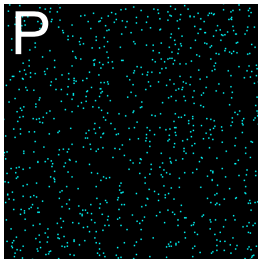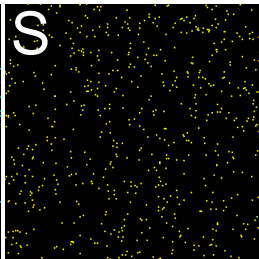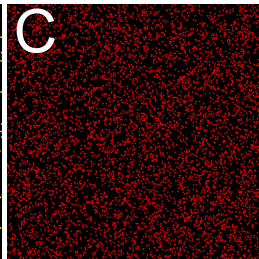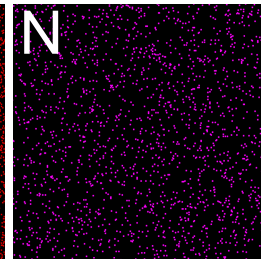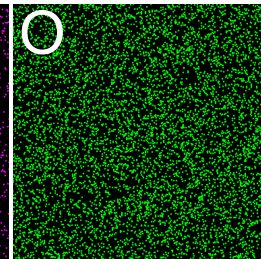

Supplement: Supplementary 1 — Figs. S1 to S31 Movies S1 to S3 Tables S1 to S6 [file research.0366.f1.zip › SI Figure/Fig. S7.pdf]

a

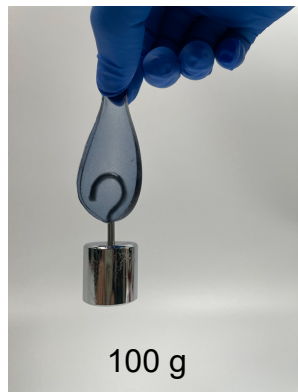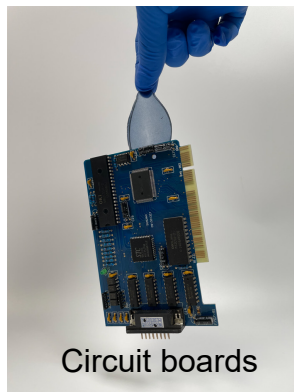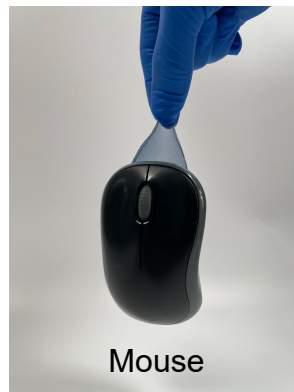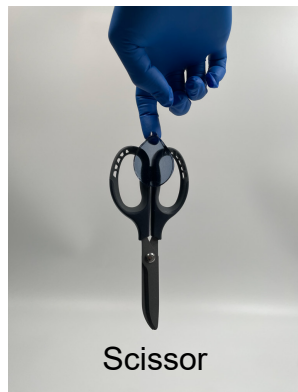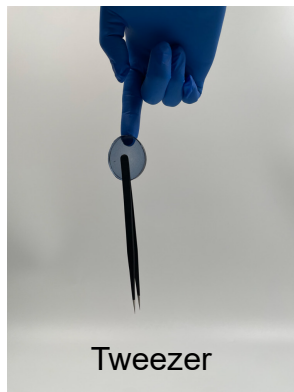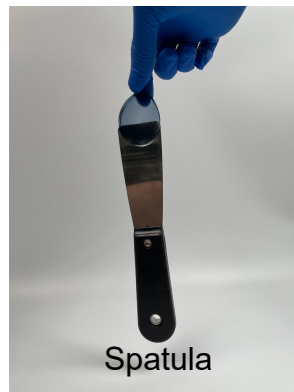

b

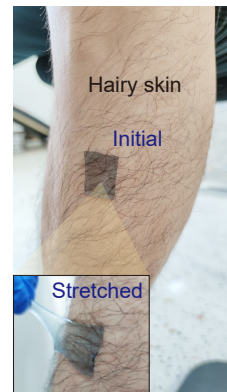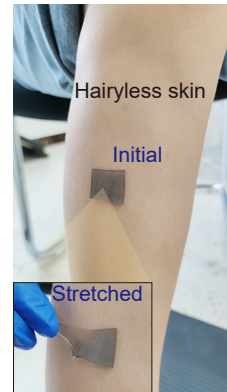

Supplement: Supplementary 1 — Figs. S1 to S31 Movies S1 to S3 Tables S1 to S6 [file research.0366.f1.zip › SI Figure/Fig. S8.pdf]

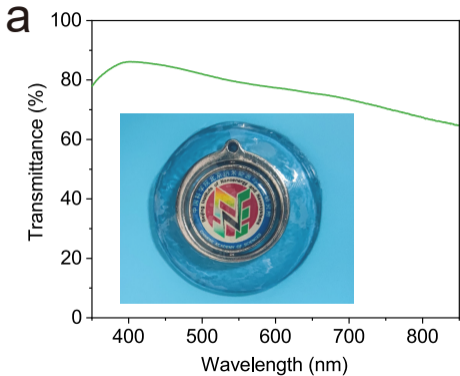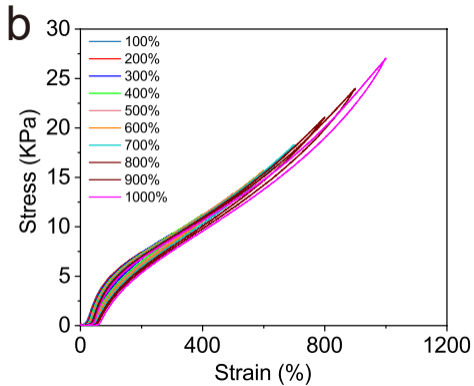

Supplement: Supplementary 1 — Figs. S1 to S31 Movies S1 to S3 Tables S1 to S6 [file research.0366.f1.zip › SI Figure/Fig. S9.pdf]
